# Rice bran supplementation modulates growth, microbiota and metabolome in weaning infants: a clinical trial in Nicaragua and Mali

**DOI:** 10.1038/s41598-019-50344-4

**Published:** 2019-09-26

**Authors:** Luis E. Zambrana, Starin McKeen, Hend Ibrahim, Iman Zarei, Erica C. Borresen, Lassina Doumbia, Abdoulaye Boré, Alima Cissoko, Seydou Douyon, Karim Koné, Johann Perez, Claudia Perez, Ann Hess, Zaid Abdo, Lansana Sangaré, Ababacar Maiga, Sylvia Becker-Dreps, Lijuan Yuan, Ousmane Koita, Samuel Vilchez, Elizabeth P. Ryan

**Affiliations:** 1 Department of Environmental and Radiological Health Sciences, Colorado State University, Fort Collins, CO 80523 USA; 2 Center of Infectious Diseases, Department of Microbiology and Parasitology, Faculty of Medical Sciences, National Autonomous University of Nicaragua, León (UNAN-León), León, Nicaragua; 3 Department of Medical Biochemistry, Faculty of Medicine, Zagazig University, Zagazig, Egypt; 4 Laboratoire de Biologie Moléculaire Appliquée, Campus de Badalabougou, Université des Sciences, des Techniques et des Technologies de Bamako, BP: 1805, Bamako, Mali; 5 Department of Statistics, Colorado State University, Fort Collins, CO 80523 USA; 6 Department of Microbiology, Immunology and Pathology, Colorado State University, Fort Collins, CO 80521 USA; 7 Departments of Family Medicine and Epidemiology, University of North Carolina at Chapel Hill, Chapel Hill, NC 27599-7595 USA; 8 Department of Biomedical Sciences and Pathobiology, Virginia-Maryland College of Veterinary Medicine, Virginia Polytechnic Institute and State University, Blacksburg, VA 24061 USA

**Keywords:** Microbiome, Randomized controlled trials, Paediatric research

## Abstract

Rice bran supplementation provides nutrients, prebiotics and phytochemicals that enhance gut immunity, reduce enteric pathogens and diarrhea, and warrants attention for improvement of environmental enteric dysfunction (EED) in children. EED is a subclinical condition associated with stunting due to impaired nutrient absorption. This study investigated the effects of rice bran supplementation on weight for age and length for age z-scores (WAZ, LAZ), EED stool biomarkers, as well as microbiota and metabolome signatures in weaning infants from 6 to 12 months old that reside in Nicaragua and Mali. Healthy infants were randomized to a control (no intervention) or a rice bran group that received daily supplementation with increasing doses at each month (1–5 g/day). Stool microbiota were characterized using 16S rDNA amplicon sequencing. Stool metabolomes were analyzed using ultra-high-performance liquid-chromatography tandem mass-spectrometry. Statistical comparisons were completed at 6, 8, and 12 months of age. Daily consumption of rice bran was safe and feasible to support changes in LAZ from 6–8 and 8–12 months of age in Nicaragua and Mali infants when compared to control. WAZ was significantly improved only for Mali infants at 8 and 12 months. Mali and Nicaraguan infants showed major differences in the overall gut microbiota and metabolome composition and structure at baseline, and thus each country cohort demonstrated distinct microbial and metabolite profile responses to rice bran supplementation when compared to control. Rice bran is a practical dietary intervention strategy that merits development in rice-growing regions that have a high prevalence of growth stunting due to malnutrition and diarrheal diseases. Rice is grown as a staple food, and the bran is used as animal feed or wasted in many low- and middle-income countries where EED and stunting is prevalent.

## Introduction

The prevalence of malnutrition in low and middle-income countries (LMIC) has negative consequences on growth of children during the first five years of life and has lifelong health consequences^1,2^. There is an increased risk of death among children under 5 years of age due to being underweight and stunted^2,3^. Risk factors for undernutrition may include, but are not limited to: low birth weight, inadequate breastfeeding, improper complementary feeding, and recurrent infections^3,4^. Diarrheal diseases are some of the primary causes of undernutrition in children under five years of age^1,3,4^.

Environmental enteric dysfunction (EED) is an acquired subclinical condition of the small intestine among LMIC children^5–10^. Chronic exposure to enteric pathogens early in life is one likely contributor to EED^11^. The altered gastrointestinal functions in EED include intestinal nutrient malabsorption and increased intestinal permeability that leads to protein loss^6,7^. Infant weaning has been identified as a critical window for intervention^12^. Previous intervention efforts in young children have targeted micronutrient deficiencies, such as Vitamin A, Zn and Fe^13–16^, oral rehydration salts for treating diarrhea^17^, antimicrobial use^18,19^, and community hygiene improvements^20^. Given the worldwide trends and variable timing of growth faltering that has been reported, assessing interventions in diverse populations merits further attention^21^.

Rice bran is a nutrient dense food with a unique profile and ratio of bioactive phytochemicals such as gamma oryzanol, tocotrienols, ferulic acid, vitamin B, beta-sitosterol and many others. The metabolome of rice bran from 17 countries was analyzed and revealed a varied suite of bioactive molecules that span multiple chemical classes^22^. Rice bran has shown chronic disease fighting properties in both animal and human studies, and the promising results from feeding neonatal pigs provided translational support for investigation of dietary feasibility in weaning infants. Many rice bran components, such as phenolics, fatty acids and soluble fibers work together to prevent enteric pathogens and diarrheal disease in mice and pigs^23–26^, and to favorably promote gut health in adults^27,28^. The effect of rice bran supplementation on host resistance to enteric infections and enhanced gut mucosal immunity was demonstrated for *Salmonella enterica* Typhimurium^25,26^, rotavirus^29–31^, and norovirus^23^. Rice bran merits attention because it has widespread scalability for consumption globally^32^, and particularly in LMIC regions where EED is prevalent^33^.

Stool EED biomarkers, gut microbiota^34^ and metabolite profiling analysis^15,35^ are important surrogate markers for analysis as intestinal tissue from infants is not easily accessible to evaluate. Stool myeloperoxidase (MPO)^36^, calprotectin (CAL)^37^, and neopterin (NEO)^38^ are indicators of inflammation; and alpha-1-antitrypsin (AAT)^36^ is an indicator of barrier lumen disruption. Chronic, elevated concentrations of all four biomarkers have been associated with poor linear growth in infants up to 24 months old^10,36–38^, and as the gut microbiota is maturing over the first 3 years of life^21,39^. Gut microbiota composition and metabolism is influenced by delivery mode, environment, and nutrition^40,41^. Recent studies have demonstrated that malnutrition and immature microbiota of infants are only partially, and temporarily improved by some nutritional interventions^15,42^. The primary objective of this study was to investigate safety and tolerability of dietary rice bran supplementation during infant weaning, including effects on growth, EED biomarkers, gut microbiota and metabolome from six to twelve months of age in Nicaragua and Mali. Nicaragua and Mali have agricultural rice production systems, yet they currently do not use the rice bran for human consumption after it is polished from the whole grain. Our study hypothesis was that daily consumption of rice bran for six months is tolerable, safe and feasible for children during weaning and that daily intake will be associated with improved growth and decreased gut permeability alongside favorable modulation of the gut microbiota and metabolome.

## Results

### Safety and feasibility

Daily rice bran consumption was completed in a randomized controlled trial with infants from 6 to 12 months of age. While the types of weaning foods differ between Mali and Nicaragua, there were no differences detected in the types of weaning foods consumed between rice bran and control group infants within each country site. Rice bran supplementation was palatable and safe for weaning infants based on a high-level of compliance and no adverse events related to rice bran intake at the increasing doses over time. Dietary compliance to rice bran was averaged per month during the 6-month intervention and was 90% in Nicaragua and 99% in Mali. The feasibility and tolerability for infants to consume 1–5 g of rice bran/day over the six-month study period was determined by how mothers fed the rice bran powder directly or as reported by consumption with drinking water, staple grain porridges (i.e. millet, sorghum, and white rice), soups, milk, fruits, juices, eggs, and fish when available. The nutritional profile of rice bran provided in this study is shown in Table 1.

**Table 1:** Nutrient composition of Rice Bran (as provided by USDA National Nutrient Database).

Nutrient	Unit	Value per 1 g	Value per 5 g	Value per 540 g
		Minimum daily consumption	Maximum daily consumption	Total consumption over 6 months
Water	g	0.0613	0.3065	33.102
Energy	kcal	3.16	15.8	1706.4
Protein	g	0.1335	0.6675	72.09
Total lipid (fat)	g	0.2085	1.0425	112.59
Carbohydrate, by difference	g	0.4969	2.4845	268.326
Fiber, total dietary	g	0.21	1.05	113.4
Sugars, total	g	0.009	0.045	4.86
**Minerals**				
Calcium, Ca	mg	0.57	2.85	307.8
Iron, Fe	mg	0.1854	0.927	100.116
Magnesium, Mg	mg	7.81	39.05	4217.4
Phosphorus, P	mg	16.77	83.85	9055.8
Potassium, K	mg	14.85	74.25	8019
Sodium, Na	mg	0.05	0.25	27
Zinc, Zn	mg	0.0604	0.302	32.616
**Vitamins**				
Vitamin C, ascorbic acid	mg	0	0	0
Thiamin	mg	0.02753	0.13765	14.8662
Riboflavin	mg	0.00284	0.0142	1.5336
Niacin	mg	0.33995	1.69975	183.573
Vitamin B-6	mg	0.0407	0.2035	21.978
Folate, DFE	µg	0.63	3.15	340.2
Vitamin B-12	µg	0	0	0
Vitamin A, RAE	µg	0	0	0
Vitamin A, IU	IU	0	0	0
Vitamin E (alpha-tocopherol)	mg	0.0492	0.246	26.568
Vitamin D (D2 + D3)	µg	0	0	0
Vitamin D	IU	0	0	0
Vitamin K (phylloquinone)	µg	0.019	0.095	10.26
**Lipids**				
Fatty acids, saturated	g	0.04171	0.20855	22.5234
Fatty acids, monounsaturated	g	0.07549	0.37745	40.7646
Fatty acids, polyunsaturated	g	0.07459	0.37295	40.2786

The minimum daily dose, maximum daily dose, and total dose of rice bran provided to infants in the Nicaragua and Mali randomized controlled trials.

### Study participant characteristics

**Figure 1:**
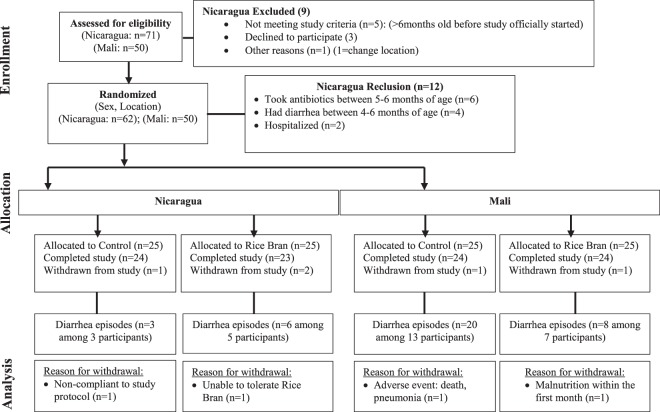
Study recruitment and participation based on CONSORT statement guidelines for clinical trials conducted in Nicaragua and Mali (NCT02615886, NCT0255737315). 95 infants from León, Nicaragua and Dioro, Mali enrolled after meeting eligibility criteria, randomized by sex and location to one of two study arms. The number of diarrhea episodes and reasons for withdrawal were reported for each child.

To study the effect of daily rice bran supplementation, monthly stool samples from 47 Nicaraguan and 48 Malian children were collected (average of 7 samples per child, total of 567 samples). The flow and number of infants from study recruitment to study completion is shown in Fig. 1. Baseline participant characteristics for Nicaragua and Mali are shown in Table 2. We collected information on demographic factors and infants’ household characteristics. In Nicaragua, 54.2% of infants were born via caesarean section in the control group and 30.4% in the rice bran group. All participants from Mali were delivered vaginally. For breastfeeding status, 96% of the control group and 83% in the rice bran group were consuming breast milk at six months old in Nicaragua and all children in the Mali group were consuming breast milk at the beginning and throughout the study. Of the 95 infants enrolled, 52 received antibiotics with 87 total antibiotic courses in the six-month period. Most courses consisted of systemic antibiotics given orally, with some delivered by injection for respiratory, skin, ear or diarrheal infections.

**Table 2:** Baseline infant participant characteristics from Nicaragua and Mali.

Variable	Nicaragua			Mali		
	Control (n = 24)	Rice bran (n = 23)	p-value^c^	Control (n = 24)	Rice bran (n = 24)	p-value^c^
**Sex (%)**						
Male	14 (58.0)	12 (52.0)	0.6711	12 (50)	12 (50)	1
Female	10 (42.0)	11 (48.0)		12 (50)	12 (50)	
**Water source (%)**						
Indoor municipal	24 (100)	23 (100)	—	0 (0)	0 (0)	—
Untreated ground water	0 (0)	0 (0)	—	24 (100)	24 (100)	—
**Delivery type (%)**						
Vagina	11 (45.8)	16 (69.6)	0.099	100 (100)	100 (100)	—
Caesarean	13 (54.2)	7 (30.4)		0 (0)	0 (0)	
**Sanitation System**						
None	0 (0)	1 (4.3)	—	0 (0)	0 (0)	—
Community latrine	0 (0)	0 (0)	—	21 (87.5)	19 (79.2)	—
Latrine	4 (16.7)	9 (39.1)	—	3 (12.5)	5 (20.8)	—
Indoor toilet	20 (83.3)	13 (56.5)	—	0 (0)	0 (0)	—
**Mother education (%)**						
None	1 (4.2)	0 (0)	—	12 (50)	11 (46)	—
Some primary	3 (12.5)	7 (30.4)	—	4 (17)	7 (29)	—
Completed primary	3 (12.5)	2 (8.7)	—	6 (25)	1 (4)	—
Some secondary	8 (33.3)	5 (21.7)	—	1 (4)	2 (8)	—
Completed secondary	4 (16.7)	5 (21.7)	—	1 (4)	3 (13)	—
University	5 (20.8)	4 (17.4)	—	0 (0)	0 (0)	—
**Breastfeeding Status (%)**						
6 months	23 (95.8)	19 (82.6)	0.1415	24 (100)	24 (100)	—
**Antibiotic Use (6–12 months)**						
# Infants antibiotic use	14 (58.3)	11 (47.8)	—	14 (58.3)	13 (54.2)	—
Antibiotic courses	21 (58.3)	15 (41.6)	—	26 (51.0)	25 (49.0)	—
**Household Animals** ^**a**^						
Poultry	3 (12.5)	9 (37.5)	0.3583	21 (88)	21 (88)	0.4091
Livestock	2 (8.3)	2 (8.7)		21 (88)	17 (71)	
Domesticated pets	17 (70.8)	16 (69.6)		5 (21)	1 (4)	
None	7 (29.2)	5 (21.7)		1 (4)	2 (8)	
**Anthropometry** ^**b**, **d**^						
Weight at Birth (kg)	3.17 ± 0.39	2.94 ± 0.38	0.0505	3.07 ± 0.45	3.29 ± 0.49	0.1146
Weight 6 months (kg)	8.09 ± 1.10	7.93 ± 0.89	0.5833	7.02 ± 0.88	7.14 ± 0.99	0.4832
Length Birth (cm)	50.67 ± 1.93	49.55 ± 3.03	0.1472	49.77 ± 1.56	50.50 ± 2.04	0.1789
Length 6 months (cm)	66.38 ± 2.10	66.26 ± 2.90	0.8787	65.57 ± 2.56	66.56 ± 3.12	0.2960
LAZ 0 months (cm)	0.87 ± 0.93	0.37 ± 1.60	0.2045	0.12 ± 0.88	0.45 ± 1.18	0.3289
LAZ 6 months (cm)	−0.03 ± 0.82	0.07 ± 1.29	0.7325	−0.30 ± 1.70	−0.15 ± 1.46	0.7427
WAZ 0 months (cm)	−0.30 ± 0.85	−0.82 ± 0.89	**0.0497**	−0.48 ± 1.04	0.02 ± 1.06	0.1389
WAZ 6 months (cm)	0.33 ± 1.09	0.27 ± 0.98	0.8412	−0.65 ± 1.29	−0.64 ± 1.09	0.9827
WLZ 0 months (cm)	−1.54 ± 1.41	−2.07 ± 1.63	0.2585	−0.92 ± 1.31	−0.39 ± 1.49	0.2571
WLZ 6 months (cm)	0.53 ± 1.25	0.41 ± 0.96	0.7107	−0.51 ± 0.95	−0.58 ± 1.05	0.7963

^a^More than one category may be represented per household. ^b^Mean ± standard deviation. ^c^p-value:Chi-squared test. ^d^Anthropometric p-values calculated by two-sample t-test.

### Anthropometric growth measurements

**Figure 2:**
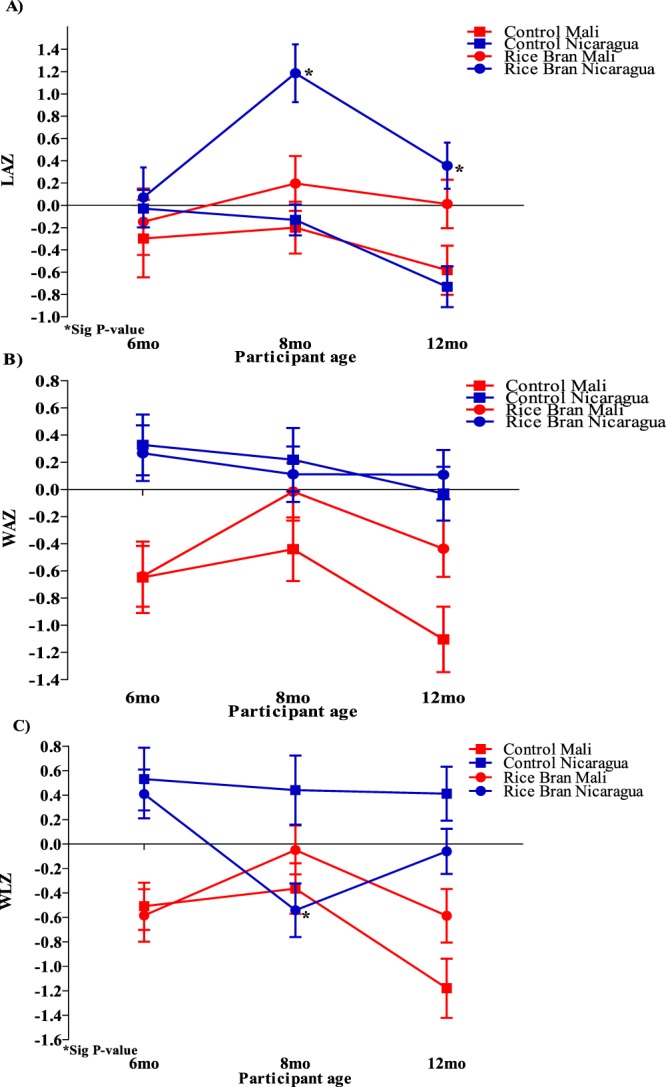
Anthropometric Z-scores for Nicaraguan and Malian infants in rice bran and control groups at 6, 8 and 12 months. (**A**) Significant LAZ (p < 0.05) at 8 and 12 months in the rice bran group compared to control for Nicaraguan infants. (**B**) No WAZ significant changes between rice bran and control group in Nicaraguan and Malian infants. (**C**) WLZ at 8 months was significantly lower for the rice bran group compared to control in Nicaragua.

Anthropometric data were collected using standardized procedures across study sites at 6, 8, and 12 months of age for each child and included length-for-age Z-score (LAZ), weight-for-age Z-score (WAZ) and weight-for-length Z-score (WLZ). Table 3 reports the z-scores analyzed for each country by repeated measures and adjusted by treatment (rice bran) and the rate of growth was significantly changed by age groups 6–8 months and 8–12 months. Significant differences were observed for anthropometric measures between treatment and ages in both cohorts. In Nicaraguan infants who consumed rice bran, LAZ was significantly changed over time (p-value = 0.0002) and for WLZ at 6–8 months (p-value < 0.0001). The Malian infants who consumed rice bran had significant growth results for WAZ (6–8 months, p-value = 0.0001, 8–12 months, p-value = 0.0175) and WLZ (6–8 months, p-value = 0.0141, 8–12 months, p-value = 0.0134). Figure 2 displays LAZ, WAZ and WLZ for each age group and by country. The significant changes observed in LAZ at 8 months and 12 months in Nicaraguan infants who consumed rice bran was compared to control group (Fig. 2A, p < 0.01). No significant differences were observed for WAZ and WLZ with this control group comparison (Fig. 2B,C).

**Table 3:** Anthropometric measures adjusted by treatment and age in Nicaraguan and Malian Infants.

Indicator	Nicaragua				Mali			
	Control		Rice Bran		Control		Rice Bran	
	n = 24^a^	p-value^b^	n = 23^a^	p-value^b^	n = 24^a^	p-value^b^	n = 24^a^	p-value^b^
**Length-for-age Z-score**								
**Months**								
6	−0.03 (0.17)	0.8689	0.07 (0.27)	**<0.0001**	−0.30 (0.35)	0.9165	−0.15 (0.30)	0.1111
8	−0.13 (0.14)		1.18 (0.26)		−0.20 (0.23)		0.19 (0.25)	
		**0.0098**		**0.0002**		0.1609		0.6229
12	−0.73 (0.18)		0.35 (0.21)		−0.58 (0.22)		0.01 (0.22)	
**Weight-for-age Z-score**								
**Months**								
6	0.33 (0.22)	0.5648	0.27 (0.20)	0.3335	−0.65 (0.26)	0.4575	−0.64 (0.22)	**<0.0001**
8	0.22 (0.23)		0.11 (0.20)		−0.44 (0.23)		−0.02 (0.21)	
		0.0558		0.9919		**<0.0001**		**0.0175**
12	−0.03 (0.20)		0.11 (0.18)		−1.10 (0.24)		−0.44 (0.21)	
**Weight-for-length Z-score**								
**Months**								
6	0.53 (0.26)	0.8997	0.41 (0.20)	**<0.0001**	−0.51 (0.19)	0.8419	−0.58 (0.21)	**0.0141**
8	0.44 (0.28)		−0.54 (0.22)		−0.36 (0.21)		−0.05 (0.20)	
		0.9892		0.0569		**<0.0001**		**0.0134**
12	0.41 (0.22)		−0.06 (0.18)		−1.18 (0.24)		−0.59 (0.22)	

^a^Mean (SEM). ^b^Ajusted p-value by repeated measures for each treatment and time point (6–8 and 8–12 months) LAZ: Length for Age Z-score, WAZ: Weight for Age Z-score, WLZ: Weight for Length Z-score.

### Diarrheal episodes and stool environmental enteric dysfunction (EED) biomarkers

For the Mali cohort, the diarrheal disease incidence was 33% in the rice bran intervention group compared to 79% in the control group over the 6-month study period. There was one rice bran intervention participant who experienced a single repeat episode of diarrhea, and 5 control group participants experienced between 2 and 4 repeat episodes of diarrhea during the 6-month trial. There were a higher number of diarrheal episodes in the Mali control group infants when compared to rice bran intervention group. No differences were detected for diarrheal episodes for the Nicaragua rice bran and control groups. We next evaluated four EED stool biomarkers at 6, 8 and 12 months of age using ELISA (see materials and methods). A significant decrease in AAT was observed at 12 months of age (p = 0.0368) in Nicaragua infants who consumed rice bran compared to control (Table 4). No significant differences were detected for the three other surrogate markers NEO, MPO and CAL between treatment groups. There was, however, a slight decrease observed in the median concentration of all stool EED biomarkers in the rice bran group compared to control for both countries.

**Table 4:** Environmental enteric dysfunction (EED) biomarkers in stool at 6, 8, and 12 months of age for Nicaraguan and Malian infants.

EED Biomarker	Nicaragua			Mali		
	Control^a^ (n = 24)	Rice Bran^a^ (n = 23)	p-value^b^	Control^a^ (n = 24)	Rice Bran^a^ (n = 24)	p-value^b^
**Neopterin (nmol/L)**						
6	150.8 (182.2)	208.6 (131.7)	0.7220	20.2 (31.0)	34.6 (28.0)	0.5929
8	222.3 (241.1)	144.8 (196.9)	0.5771	36.2 (27.6)	28.9 (28.5)	0.4314
12	137.5 (285.2)	182.4 (230.4)	0.8727	12.0 (33.0)	36.0 (32.6)	0.9880
**Myeloperoxidase (ng/ml)**						
6	277.0 (374.5)	237.5 (376.5)	0.0847	3970.6 (17776.6)	5400.9 (20794.8)	0.6139
8	331.1 (312.3)	266.3 (236.4)	0.8454	15838.7 (20511.9)	7451.0 (12972.7)	0.3763
12	182.0 (324.8)	158.5 (376.7)	0.3345	4846.4 (11266.9)	3153.9 (14095.2)	0.5437
**Calprotectin (µg/g)**						
6	32.2 (108.7)	88.0 (281.1)	0.2394	51.7 (101.2)	53.3 (106.4)	0.7136
8	24.7 (87.5)	58.2 (213.2)	0.8023	130.0 (769.7)	68.7 (126.8)	0.0639
12	24.0 (74.4)	50.5 (133.9)	0.2629	35.0 (70.2)	20.9 (56.0)	0.2103
**Alpha-1 Antitrypsin (ng/ml)**						
6	130.1 (177.7)	109.5 (217.4)	0.7199	247.5 (499.6)	463.1 (891.8)	0.0999
8	152.0 (115.4)	73.5 (122.4)	0.1221	619.2 (759.0)	579.9 (899.9)	0.2237
12	130.9 (129.8)	70.8 (87.8)	**0.0368**	663.7 (580.5)	453.2 (807.5)	0.4796

^a^Median (IQR).

^b^p-value by repeated measures comparing treatments at each time point.

### Infant gut microbiota

**Figure 3:**
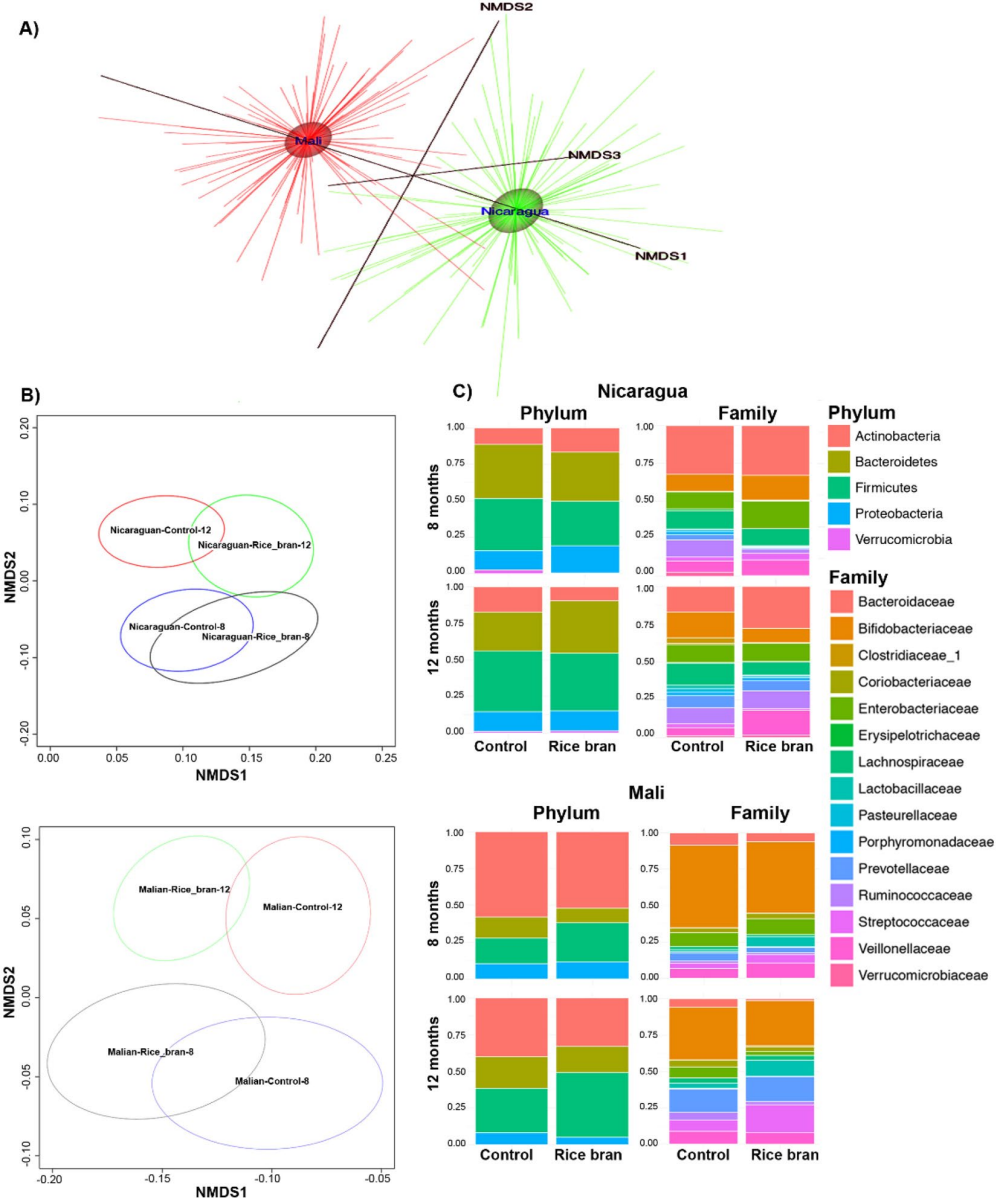
Rice bran and control infant stool microbiota at 8 and 12 months of age in Nicaragua and Mali. Nonmetric Multidimensional Scaling (NMDS) for (**A**). Nicaragua and Mali all samples (**B**). Control groups and rice bran groups at 8 and 12 months. NMDS was used on the OTU level to assess possible trends and clustering in the microbial community structure per treatment and time point. (**C**) Bacterial taxa at phylum and family level in Nicaragua (top) and Mali (bottom). Bar-graphs show phylum and family relative abundance based on the resulting OTU table generated using the ggplot2 package in R. These plots were generated for the data at the phylum and the family levels and meant to describe the microbial community structure per sampled group and per time point (8 months and 12 months) under each of the treatment levels (control and rice bran).

The microbiota were characterized and compared for 48 Malian and 47 Nicaraguan infants at 8 and 12 months of age in the rice bran and control study groups. DNA was isolated from stool samples and the V4 hypervariable region of the 16S rRNA gene was sequenced utilizing the Earth Microbiome Project protocol^43–47^. Sequences were preprocessed for quality assurance and classified into operational taxonomic units (OTUs) (see materials and methods), and the results were integrated to construct family and genus-level composition profiles for all 192 samples from both countries. No major differences were detected in alpha diversity indices (Observed, Shannon, InvSimpson and Richness) calculated for rice bran group and control group at 8 and 12 months (Table S1). A complete country-level separation in the overall gut microbial community composition was depicted for beta diversity using Nonmetric Multidimensional Scaling (NMDS) plot based on the Bray-Curtis distance measure (Fig. 3A). The magnitude of age and country-level differences in infant gut microbiota composition was expected, as differences were previously reported that compared these geographical regions^21^. The differences in microbiota composition at baseline provided compelling rationale for separately evaluating the control and rice bran responsive microbial communities in infants at each age and for each country.

**Figure 4:**
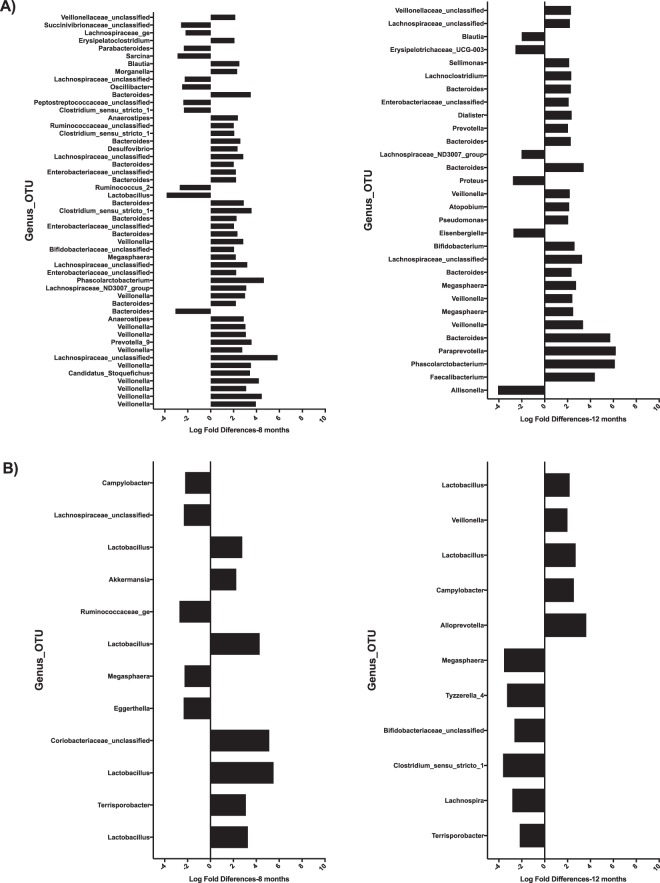
Microbiota differences between Nicaragua and Mali at 8 and 12 months between rice bran and control groups. Fold differences in relative percentage of OTUs different between control and rice bran groups at 8 months and 12 months. (**A**) Nicaragua, and (**B**) Mali. OTUs with fold difference more than 2 are shown for infants at 8 months (left) and 12 months (right). Fold difference for OTUs with FDR less than 0.05 is shown with the most significant OTUs on the bottom of each graph.

Figure 3B shows the NMDS plot separated by country. This figure highlights differences in the microbiota between two time periods, 8 and 12 months, which is more pronounced in the Malian samples. There is overlap of microbiota within each time period that indicates putative similarity between microbial community structures during growth. The overlap was observed to a greater level at 8 months of age as compared to 12 months that may illustrate microbial adaptation to new exposures^33^. Figure 4 illustrates the OTU with at least 2 log-fold change between the rice bran and control groups per country and per age group. These OTU were ordered based on statistical significance (FDR-adjusted p-value, from bottom (lowest p-value) to top). See additional details provided in Table S2 for Nicaragua and Table S3 for Mali. The effects of daily dietary rice bran supplementation on the infant microbiota were evaluated at 8 months of age for each country because this was the time point in which measurable growth differences (LAZ) were already observed for the rice bran group compared to control.

In Nicaragua, differential abundance testing led to the identification of 145 OTUs that were significantly different between control and rice bran (Table S2). There were 74 OTUs that showed greater than or equal to 2 log fold differences between rice bran and control groups at 8 or 12 months (Fig. 4A, adjusted p-value < 0.05). Seven of these 74 OTUs overlapped between the ages 8 and 12 months. In Mali, 42 bacterial OTUs were identified to significantly differ between control and rice bran across samples, and 19 showed more than 2 log fold changes between rice bran and control at 8 or 12 months (Fig. 4B, adjusted p-value < 0.05) with only three overlapping between the two age groups. Next, we explored the genus level assignments that were responsive to rice bran intake for each country. For Nicaraguan infants (Table S2), the notable rice bran responsive taxa which changed at 8 months of age compared to control group were Lachnospiraceae-unclassified-Otu0280 (log-FC 5.84, adjusted p-value 3.95E-08) Bifidobacterium-unclassified-Otu0314 (log-FC 2.04, adjusted p-value 1.38E-6), Ruminococcaceae-unclassified-Otu0238 (log-FC 2.01, adjusted p-value 0.00097), Veillonella (11different OTUs, log-FC > 2.0, adjusted p-value < 0.05), and Bacteroides (log-FC > 2.0, adjusted p-value < 0.05). The fold difference for genus level taxa that were lower in relative percent abundance for rice bran group were Bacteroides-Otu0192 (log-FC −3.08, adjusted p-value 2.34E-07), Parabacteroides-Otu0086 (log-FC −2.34, adjusted p-value 0.0074), Lachnospiraceae-unclassified-Otu0174 (log-FC −2.27, adjusted p-value 0.0033), Lactobacillus-Out0053 (log-FC −3.85, adjusted p-value 1.09E-05), Oscillibacter (log-FC −2.49, adjusted p-value 0.0029) and Ruminococcaceae_2 (log-FC −2.69, adjusted p-value 1.98E-05).

In Mali infants, (Table S3), there were 2-fold increased differences observed for rice bran fed infants in Lactobacillus-Out0356 (log-FC 3.2, adjusted p-value 1.35E-09) and decreased for Lachnospiraceae-unclassified-Otu0010 (log-FC −2.3, adjusted p-value 0.016). The infant gut microbiota showed changes that were observed by age and for each country with respect to rice bran intake. At 12 months of age, the Nicaragua rice bran group had increased Paraprevotella (log-FC 6.2, adjusted p-value 4.27E-08), Phascolarctobacterium (log-FC 6.12, adjusted p-value 1.60E-08) Veillonella (log-FC 3.35, adjusted p-value 3.30E-07) and Bifidobacterium (log-FC 2.6, adjusted p-value 1.44E-05). Lower abundant taxa in rice bran infants at 12 months of age from Nicaragua were Lachnospiraceae_ND3007_group (log-FC −2.0, adjusted p-value 0.00029) and Alisonella (log-FC −4.0, adjusted p-value 1.60E-08). Malian rice bran fed infants at 12 months of age showed increased Lactobacillus_Otu0053 (log-FC 2.7, adjusted p-value 0.0098) and Alloprevotella (log-FC 3.6, adjusted p-value 0.00034). The significant changes observed for Malian infants between rice bran and control groups at 12 months were Bifidobacteriaceae_unclassified_Otu0265 (log-FC −2.6, adjusted p-value 3.95E-05), Clostridium_sensu_stricto_1_Otu0076, and Terrisoporobacter (log-FC −2.1, adjusted p-value 7.03E-06).

Gut microbiota changes that were reflective of a dietary response to rice bran in both the Mali and Nicaragua cohorts involved 12 OTUs at either 8 or 12 months of age when compared to control. The highest area of overlap occurred for both Lactobacillaceae_Lactobacillus_Otu0024 and the Lactobacillus_Otu0053 (Table S4). There was additional overlap in both countries with respect to microbiota changes following rice bran intake that included Bifidobacterium, Faecalibacterium, and Lachnospiriaceae.

### Infant stool metabolomes

A total of 309 stool samples were analyzed for metabolomics from this 6-month prospective study to evaluate the effects of rice bran supplementation compared to control. Stool metabolite analysis at 8 months of age in Nicaraguan and Malian infants resulted in the detection of 1449 biochemicals, of which 1016 metabolites had confirmed names and 433 compounds were of unknown structural identity (see Table S5). ANOVA contrasts and Welch’s two-sample t-test were used to identify biochemicals that significantly differed between experimental groups at 8-months of age. Table 5 lists the fold differences in the stool metabolite relative abundance between study diet groups at 8 months of age. There are 39 (Nicaragua) and 44 (Mali) stool metabolites with significant fold differences between children consuming rice bran for two months when compared to control. There were also 33 unknown metabolites for Nicaragua and 31 unknown metabolites for Mali that showed significant differences between groups (data shown in Table S5). Significant fold differences occurred for 15 amino acids, 2 peptides, 3 carbohydrates, 9 lipids, 1 cofactor and vitamin, and 9 xenobiotics (six of these metabolites were considered to be food components/plant-derived) in Nicaraguan children who consumed rice bran compared to control. At 8 months of age, the stool from Mali infants had 6 amino acids, 1 energy metabolite, 14 lipids, 6 cofactor and vitamins, 5 nucleotides and 12 xenobiotics (7 classified as food components/ plant-derived) that were significantly different between rice bran and control (Table 5).

**Table 5:** Stool metabolites significantly modulated by rice bran supplementation compared to control for Nicaragua & Mali infants at 8 months of age.

Metabolic Pathway	Metabolite^a^	HMDB^b^	Nicaragua		Mali	
			Fold Diff^c^	p-value	Fold Diff^c^	p-value
**Cofactors and Vitamins**						
Nicotinate and Nicotinamide Metabolism	Quinolinate	HMDB00232	0.88	0.7272	0.44	0.0313
	**Nicotinate**	HMDB01488	1.15	0.3958	1.6	0.0053
Tocopherol Metabolism	**alpha-tocotrienol**	HMDB06327	0.69	0.3922	3.01	0.0168
	**gamma-tocotrienol**	HMDB12958	0.67	0.1909	2.53	0.0044
	gamma-CEHC glucuronide*		0.41	0.0035	0.96	0.8862
Vitamin B6 Metabolism	**pyridoxine (Vitamin B6)**	HMDB02075	1.58	0.3051	4.65	0.0011
	**Pyridoxate**	HMDB00017	0.86	0.5441	2.34	0.0014
**Xenobiotics**						
Benzoate Metabolism	**4-hydroxybenzoate**	HMDB00500	0.88	0.6260	1.8	0.0272
	methyl-4-hydroxybenzoate	HMDB32572	0.57	0.0299	0.9	0.7061
	**3-(4-hydroxyphenyl)propionate**	HMDB02199	1.08	0.8898	6.94	0.0007
Xanthine Metabolism	**Theophylline**	HMDB01889	0.86	0.6266	0.44	0.0091
Food Component/Plant	**Indoleacetylaspartate**	HMDB38666	1.1	0.7543	2.14	0.0134
	**Vanillate**	HMDB00484	0.82	0.5775	2.27	0.0246
	deoxymugineic acid		0.6	0.2733	4.5	0.0021
	**dihydroferulic acid**		0.63	0.4522	6.99	0.0032
	**Ferulate**	HMDB00954	1.24	0.6403	3.5	0.0100
	**ferulic acid 4-sulfate**	HMDB29200	1.14	0.8494	4.88	0.0274
	ferulylglycine (1)		0.4	0.0256	2.16	0.0665
	Rosmarinate	HMDB03572	0.57	0.0206	1.28	0.3008
	**Tyrosol**	HMDB04284	1.01	0.9832	1.98	0.0412
	**Diosmetin**	HMDB29676	0.29	0.0192	1.25	0.6872
	daidzein sulfate (2)		0.39	0.0123	1.38	0.4129
	daidzein sulfate (1)		0.35	0.0023	1.04	0.9083
	**Salicylate**	HMDB01895	1.78	0.0970	4.67	0.0000
	**N-propionylmethionine**		1.09	0.8631	3.6	0.0111
	malonylgenistin		0.51	0.0023	0.99	0.9670
Drug - Analgesics, Anesthetics	4-acetamidophenylglucuronide	HMDB10316	0.99	0.0484	1	1.0000
	2-methoxyacetaminophen glucuronide*		0.77	0.0049	1	1.0000
**Amino Acid**						
Glycine, Serine and Threonine Metabolism	**Glycine**	HMDB00123	0.66	0.0062	1.2	0.2384
	dimethylglycine	HMDB00092	0.69	0.2880	0.42	0.0153
Lysine Metabolism	N6-formyllysine		0.6	0.1944	2.37	0.0385
Phenylalanine Metabolism	**phenylpyruvate**	HMDB00205	0.59	0.0332	1.18	0.5305
	**phenyllactate (PLA)**	HMDB00779	0.44	0.0223	1.49	0.2855
Tyrosine Metabolism	**4-hydroxyphenylpyruvate**	HMDB00707	0.55	0.0210	0.92	0.7455
	vanillic alcohol sulfate		0.7	0.2572	2.01	0.0378
Tryptophan Metabolism	**kynurenate**	HMDB00715	0.48	0.0079	1.13	0.6565
	N-formylanthranilic acid	HMDB04089	1.2	0.5416	0.51	0.0321
	**indolepropionate**	HMDB02302	4.67	0.0189	1.33	0.6727
Leucine, Isoleucine and Valine Metabolism	alpha-hydroxyisocaproate	HMDB00746	0.45	0.0325	1.05	0.8904
	**alpha-hydroxyisovalerate**	HMDB00407	0.47	0.0335	1.08	0.8323
	**3-methyl-2-oxobutyrate**	HMDB00019	0.52	0.0299	1.4	0.2751
	**2-hydroxy-3-methylvalerate**	HMDB00317	0.46	0.0224	1.14	0.7078
Methionine, Cysteine, SAM and Taurine Metabolism	**N-acetylmethionine**	HMDB11745	0.89	0.8154	3.24	0.0214
	**N-formylmethionine**	HMDB01015	0.56	0.2035	2.84	0.0294
	**cysteine**	HMDB00574	0.66	0.0498	1.29	0.2445
	hypotaurine	HMDB00965	0.52	0.0439	0.9	0.7512
Urea cycle; Arginine and Proline Metabolism	**dimethylarginine (SDMA** + **ADMA)**	HMDB01539	0.53	0.0064	0.92	0.7445
Glutathione Metabolism	**5-oxoproline**	HMDB00267	0.5	0.0085	1.27	0.3819
	2-hydroxybutyrate/2-hydroxyisobutyrate		0.45	0.0153	0.73	0.3554
**Peptide**						
Gamma-glutamyl Amino Acid	**gamma-glutamylglutamine**	HMDB11738	0.49	0.0278	1.05	0.8844
	gamma-glutamyl-epsilon-lysine	HMDB03869	0.49	0.0137	0.84	0.5553
**Carbohydrate**						
Glycolysis, Gluconeogenesis, and Pyruvate Metabolism	**pyruvate**	HMDB00243	0.46	0.0237	1.26	0.5202
Disaccharides and Oligosaccharides	3-sialyllactose	HMDB00825	0.8	0.0484	1	1.0000
	Lewis a trisaccharide		1.36	0.0443	0.99	0.9520
**Energy**						
TCA Cycle	**alpha-ketoglutarate**	HMDB00208	0.65	0.1785	1.96	0.0484
**Lipid**						
Fatty Acid, Dicarboxylate	pimelate (C7-DC)	HMDB00857	0.93	0.7811	2.13	0.0078
Fatty Acid Metabolism (Acyl Choline)	palmitoloelycholine		1.17	0.1301	0.74	0.0070
	linoleoylcholine*		1.85	0.0459	1.5	0.2118
Fatty Acid, Monohydroxy	**8-hydroxyoctanoate**	HMDB61914	0.75	0.1576	1.61	0.0224
Fatty Acid, Dihydroxy	**12,13-DiHOME**	HMDB04705	0.89	0.7839	2.78	0.0246
	**9,10-DiHOME**	HMDB04704	0.9	0.7816	3.75	0.0012
Monoacylglycerol	1-linolenoylglycerol (18:3)	HMDB11569	2.36	0.0457	1.01	0.9871
Diacylglycerol	linoleoyl-linolenoyl-glycerol (18:2/18:3) [1]*	HMDB07249	2.04	0.0285	1.18	0.6201
	linolenoyl-linolenoyl-glycerol (18:3/18:3) [2]*	HMDB07278	2.29	0.0404	0.76	0.5121
	linoleoyl-docosahexaenoyl-glycerol (18:2/22:6) [1]*		1.03	0.8045	0.66	0.0006
	linoleoyl-docosahexaenoyl-glycerol (18:2/22:6) [2]*	HMDB07266	0.98	0.9291	0.59	0.0165
Sphingolipid Metabolism	**N-acetylsphingosine**	HMDB04950	1.11	0.7987	2.44	0.0387
Mevalonate Metabolism	**3-hydroxy-3-methylglutarate**	HMDB00355	0.74	0.4206	2.77	0.0098
Sterol	**beta-sitosterol**	HMDB00852	0.77	0.3699	2.89	0.0006
	**stigmasterol**	HMDB00937	1.14	0.6191	2.01	0.0159
	**campesterol**	HMDB02869	0.86	0.6689	2.1	0.0415
Androgenic Steroids	**5alpha-androstan-3alpha,17alpha-diol disulfate**		0.56	0.0199	0.63	0.0625
	androstenediol (3beta,17beta) disulfate (2)	HMDB03818	0.71	0.2185	0.54	0.0324
Primary Bile Acid Metabolism	**glycocholate**	HMDB00138	0.39	0.0483	1.37	0.5237
	**glycochenodeoxycholate**	HMDB00637	0.43	0.0434	0.64	0.3052
	glycochenodeoxycholate glucuronide (2)		0.37	0.0195	0.78	0.5717
	glycochenodeoxycholate sulfate		0.57	0.2283	0.35	0.0284
Secondary Bile Acid Metabolism	7alpha-hydroxycholestenone	HMDB01993	0.64	0.0411	0.88	0.5716
**Nucleotide**						
Purine Metabolism, Adenine containing	N6-dimethylallyladenine		0.94	0.8249	0.28	0.0000
Purine Metabolism, Guanine containing	**Guanine**	HMDB00132	0.47	0.0876	3.22	0.0112
Pyrimidine Metabolism, Orotate containing	**N-carbamoylaspartate**	HMDB00828	1.14	0.5814	0.59	0.0326
Pyrimidine Metabolism, Uracil containing	**uridine-2′,3′-cyclic monophosphate**	HMDB11640	1.27	0.3081	1.75	0.0243
Pyrimidine Metabolism, Thymine containing	**3-aminoisobutyrate**	HMDB03911	1.23	0.6961	0.27	0.0175

^a^Table displays metabolites with statistically significant differences between rice bran and control group in stool. Bold metabolites are present in the rice bran (Calrose) that the children consumed.

^b^HMDB refers to the Human Metabolome Database.

^c^Fold differences (Fold Diff) between study groups was calculated by dividing the scaled relative abundance of rice bran vs control.

There were 62 stool metabolites from the Nicaraguan cohort at 8 months that had lower relative abundances, and there were 10 stool metabolites with significantly increased fold differences in abundance between groups. The stool metabolites of food and nutritional relevance that were associated with increasing intake of rice bran were classified to the tryptophan metabolism pathway (e.g. indolepropionate), as well as monoacylglycerol (1-linolenoylglycerol) and diacylglycerol metabolic (linoleoyl-linolenoyl-glycerol) pathways.

In contrast to Nicaragua, there were 54 distinct stool metabolites from the Mali cohort that increased with significant fold differences at 8 months between rice bran and control infants. Other stool metabolites with gut health and nutritional importance that originated from rice bran food intake, included alpha-tocotrienol (vitamin E component), pyridoxine (vitamin B6), ferulic acid 4-sulfate, tyrosol, and N-acetyl sphingosine (Table 5). An estimated false discovery rate (q-value) was calculated to account for the multiple comparisons across metabolites that are typical of metabolomic-based studies.

## Discussion

This study demonstrated that rice bran supplementation was feasible and safe for weaning infants with strong compliance in two distinct LMIC countries. Rice bran was well tolerated in a dose-escalation, supplementation trial regimen during the first 6 months of weaning, and was without side effects or adverse interactions. The distinct agro-ecological climates, dietary patterns, and birth methods between infants in West Africa and Latin America were considered highly relevant sources of variation that added scientific rigor to the generalizability of these clinical trial results with respect to safety, feasibility, and tolerability. However, when evaluating the infant gut microbiome and stool metabolite responses to increasing rice bran intake, this study showed distinct country and age specific variability in weaning infants. Rice bran supplementation in the diet supported the healthy growth of Nicaraguan and Malian infants with changes detected in LAZ between 6–8 months and 8–12 months of age. Nicaraguan infants had reduced gut permeability as measured by AAT at 12 months. Malian infants also showed changes over time for WAZ. The 95 healthy infants enrolled in this study had slightly higher WAZ, LAZ and WLZ scores than the documented country-wide averages that use the WHO scoring index^12^.

There is a growing body of scientific evidence for a strong relationship between EED and growth deficits in children^7,10,48^. EED biomarkers were selected in this study because high concentrations of stool AAT and MPO were associated with decreased growth in children^48^, Naylor *et al*. found that elevated AAT levels were associated with decreased oral rotavirus vaccine response^49^, and were associated with seroconversion to rotavirus vaccination in Nicaraguan infants^37^. Rice bran was shown to reduce AAT in neonatal pigs challenged with human Rotavirus infection^24^ and was a finding of translational importance to this clinical trial because rice bran intake improved AAT levels in Nicaraguan infants (Table 4). The lack of any statistically significant differences in EED stool biomarkers for Mali infants may be due to the higher number of overall diarrheal episodes that this cohort experienced, the level of variability within individuals, and breastfeeding status for all infants throughout the study period. Our findings did concur with EED biomarker concentrations reported in the MAL-ED cohort^36^. EED biomarkers merit continuous review for relevance with anthropometric measures due to extensive global variability in concentrations reported across studies^6,50–52^.

A study limitation and possible confounder of rice bran effects to modulate the infant gut microbiota was the percentage of exclusively breastfed infants and the number of vaginal deliveries being lower in the Nicaragua cohort. This was in contrast to the Mali cohort that had 100% of children who were breastfed and had vaginal delivery. These are key considerations to evaluating microbiota that has been previously characterized as less mature^53^, and with varied structure by geographical location^54^, diet deficiencies^55–59^, environmental exposures^54,60,61^ and host factors^60,62^. Infants from each country showed favorable changes in both the stool microbiota and metabolome over time (with increasing age and growth), and between rice bran and control groups at 8 months of age.

Given that dietary rice bran intake has been previously shown to promote beneficial stool microbial communities, such as native gut probiotics in mice^25,63^, pigs^23,24,31^ and adults^27,64,65^, differential abundance testing at the genus level assignment between rice bran and control fed infants was conducted at 8 months and 12 months of age. In Nicaragua, the Lactobacillus, Lachnospiraceae, Bifidobacterium, Ruminococcaceae and Veillonella were identified as responsive following rice bran consumption compared to an age and control matched group. These taxa have recognized saccharolytic mechanisms of action^66–68^, are known to produce and promote crossfeeding of short chain fatty acids^69,70^, as well as provide competitive inhibition of pathogen colonization^71,72^. The microbial enrichment of these communities has important implications for assessing how gut microbes metabolize rice bran that can support changes to infant LAZ growth outcomes. In Mali, the observed increase in the relative abundance of Lactobacillus in rice bran fed infants was consistent with prior studies of rice bran feeding to young animals. This finding was considered alongside evidence for human milk oligosaccharides that also promote Lactobacillus in breastfed infants^73^. Rice bran metabolism by Lactobacillus spp. was shown to produce a suite of small molecules that may be absorbed by the host or detected in stool. Stool metabolites from infants fed rice bran showed significant fold differences amongst several essential amino acids, cofactors and vitamins, lipids, phytochemicals, and in energy metabolism pathways when compared to the control. The stool metabolites that were highly likely to have originated from the rice bran consumed by both Nicaragua and Malian infants provided additional confirmation of compliance to the dietary intervention^74^. As predicted, we observed and reported exceptionally distinct profiles for both the stool microbiota and metabolome composition of infants between Mali and Nicaragua at all ages^41,54,75^ and therefore separately discussed the response to rice bran supplementation by each region. For example, the nearly 5-fold increase of stool indolepropionate in rice bran infants compared to control from Nicaragua represents a tryptophan metabolite produced by the gut microbiota that may influence the developing immune system and intestinal homeostasis^76,77^. Increased levels of N-acetylmethionine (nutritionally and metabolically equal to L-methionine) and N-formylmethionine in Malian infants also represented rice bran derived amino acids required for normal growth and development^78^. There are several cofactors and vitamins from rice bran supplementation, such as alpha and gamma-tocotrienol and pyridoxine (vitamin B6) that merit attention for demonstrating multiple health benefits such as synthesis of amino acids and neurotransmitter precursors, as well as preventing anemia and skin problems^74,79,80^. Additional microbial digested food components in the stool metabolome that come from rice bran were ferulic acid 4-sulfate, indoleacetylaspartate, and Tyrosol. This trial had only supplemented the rice bran for a 6 month window and according to WHO, growth assessment should be standardized and compared globally over the first two years of life^81^. We put forth that rice bran metabolism by host and gut microbes between 12–36 months of age should be captured in future assessments of early influences on growth velocity^81,82^. The focus on changes between 8 and 12 months in this study showed modifications in gut microbial communities and metabolites by rice bran intake, and suggests there will be long-term impact on the overall microbiota composition as it continues to develop and mature^83^.

This was the first randomized controlled trial of rice bran supplementation in LMIC infants and provides compelling rationale for continued follow-up investigation of rice bran supplementation for reducing risk of malnutrition, as well as for eliciting changes during child growth that protect against enteric pathogens and diarrheal diseases. The dose and feasibility outcomes from this study support development of rice bran based complementary weaning foods. Incorporating rice bran from local rice production and processing facilities should be a priority in subsequent trial designs. The development of sustainable, local and affordable food products for consumption by weaning infants and throughout childhood is particularly critical in regions where political instability continuously threaten the regions food and nutritional security.

## Materials and Methods

### Study design

A 6-month, prospective, randomized-controlled, phase one dose escalation dietary intervention was conducted in a cohort of weaning infants residing in León, Nicaragua and in the community of Dioro, Mali, West Africa. Nicaraguan infants were recruited from public health rosters provided by the local Health Ministry from Perla Maria and Sutiava health sectors, and Malian infants were recruited from the Dioro Community Health Center. To be eligible, male and female infants were screened between 4–5 months of age for health status, and then followed weekly for diarrhea episodes. Participants were excluded if they had experienced diarrhea or received antibiotic treatment within the previous month; had known allergies, or immune-compromising conditions (e.g. parasitic or malarial infections); had previously been hospitalized; and/or enrolled in a malnutrition treatment program. In Nicaragua, all eligible participants received 3 doses of the rotavirus vaccine per regular administration through the Immunization Program^82^. Rotavirus vaccination was not yet administrated to the Mali cohort. All Malian participants received vitamin A supplementation upon enrollment. Dietary intervention with rice bran started when infants were 6 months of age because WHO guidelines promote exclusive breastfeeding for the first six months of life^84,85^.

The required ethical board reviews and approvals were completed for Mali and Nicaragua as provided by the Internal Review Board (IRB) of the Colorado State University Research Integrity and the Compliance Review office (protocol ID# 14-5233 H Nicaragua, 15-5744 H Mali). In Mali, the Institut National de Recherche en Santé Publique (National Institute of Research in Public Health, FWA 00000892) approved the intervention, which occurred between October 2015 and May of 2016 and registered at clinicaltrial.gov as (NCT02557373) on 23 September 2015. Ethical review and approvals for the Nicaraguan intervention that occurred between March 2015 and October 2015 were provided by the IRBs of the Universidad Nacional Autónoma de Nicaragua – León, University of North Carolina at Chapel Hill, and Virginia Polytechnic Institute and State University and registered at clinicaltrial.gov on 26 November 2105 as (NCT02615886). Written informed consent was obtained from the infant’s parent or responsible guardian prior to any data collection. Infant participants that met the eligibility criteria were randomized within each health sector, and sex (Nicaragua) and geographic location of household and sex (Mali) to either rice bran or control group (see Fig. S1 for enrollment details). Randomization was completed using sequential enrollment for each site independently. Participants were randomized by CSU, enrolled and assigned to groups by study coordinators in each country. The participants were not blinded. Complete study protocol is available online (http://csu-cvmbs.colostate.edu/academics/erhs/Pages/elizabeth-ryan-lab-global-health.aspx).

### Rice bran packaging for consumption

The United States Department of Agriculture-Agricultural Research Service (USDA-ARS) Dale Bumpers National Rice Research Center provided rice bran that was polished from the U.S. variety, Calrose. Rice bran is prone to fat oxidation and heat-stabilization was performed to increase shelf-life by heating the bran at 100 degrees Celsius for five minutes to inactivate the lipase/lipoxygenase enzymes that cause rancidity^86^. The rice bran was sifted to remove any debris (rice husk, rice grain). Packaging of the rice bran was completed by Western Innovations, Inc. (Denver, CO) where 22 kg of rice bran was weighed into 1 g increments, separated into water-proof sachets, and heat-sealed to ensure the rice bran would be administered with accurate doses to infants.

Fourteen sachets (1 g/sachet) were filled into a 4″ × 3″ × 2″ box that was labeled for study participants and included nutrient information. These boxes were stored in a cool, dark, dry place until they were provided to study participants.

### Nicaragua and Mali intervention

The study team (doctor, nurse and study coordinator) in Nicaragua and the community health workers (CHWs) in Mali provided a 2-week supply of rice bran at each routine home visit and instructed the participant’s parent or guardian to add the daily amount of rice bran to the participant’s food. At 6–7 months of age, participants in the rice bran group consumed 1 g of rice bran/day (1 sachet). Between the ages of 7–8 months, participants consumed 2 g of rice bran/day (2 sachets). At 8–10 months of age, participants consumed 3 g of rice bran/day (3 sachets). The amount increased to 4 g of rice bran/day (4 sachets) from 10–11 months, and 5 g of rice bran/day (5 sachets) from 11–12 months of age, respectively. The rice bran was added to appropriate weaning foods, such as rice cereal, yogurt, fruit and natural juices, vegetables, and soups. At the beginning of the intervention (six months of age), infant’s parents or guardians were instructed and monitored daily for one week by study personnel to ensure that guardians knew how to administer and record the amount of rice bran consumed. Compliance to the rice bran intervention was calculated from records that had the dose/amount of rice bran consumed circled in daily increments of none (0%), half (50%), or all (100%). The study team also collected any unused boxes or sachets during these visits. Participants in the control group did not receive any rice bran and there were no reports of brown rice intake during the 6-month study duration.

In Nicaragua, study personnel visited all infants weekly. In Mali, the CHWs visited each participant’s household daily for the duration of the 6-month study to assess compliance and diarrhea episodes. If a participant had a diarrhea episode, the study team would collect a stool sample, and collect information that included the diarrhea onset date, how long the episode lasted, numbers of bowel movements within 24 hours, any associated signs and symptoms (e.g. nausea, vomiting, fever), if any other family members had diarrhea, and if any treatment was provided (e.g. antibiotics, rehydration).

The study team in Nicaragua collected data for control group participants at 6, 8, and 12 months old, and rice bran group every month. The anthropometric measures (weight and length) were collected via a portable stadiometer and weighing balance. Mali participants visited the Community Health Clinic every month. Length was measured in supine position using a reclining length-board. Length was collected to the nearest centimeter and weight to the nearest 0.1 kg. Anthropometric measures were calculated for LAZ, WAZ, and WLZ scores following the World Health Organizations (WHO) child growth standards using the WHO Anthro software (version 3.2.2)^87^.

Diapers were provided to all study participants. Stool was collected directly from soiled diapers. Freshly collected stool was diluted 20-fold and homogenized in a sterile pre-reduced anaerobic saline −0.1 M potassium phosphate buffer (pH 7.2) containing 20% glycerol (vol/vol). Four aliquot suspensions were prepared in 15 mL falcon tubes, transported on dry ice to the UNAN-León-Center of Infectious Diseases Laboratories (and liquid nitrogen in Bamako,Mali), immediately transferred to a −80 °C freezer, shipped in a liquid nitrogen chilled dry shipping dewar to Colorado State University, where they were relocated into a −80 °C freezer prior to analysis.

A study questionnaire was completed by the participant’s caretaker (e.g. mother, father, or grandparent) to assess for duration of breastfeeding, types of and timing of introductions to complementary foods, as well as antibiotic use. The breastfeeding questions included whether or not the child was receiving breast milk, and/or had the child been receiving received formula. The complementary feeding history included a list of common Nicaraguan and Malian foods that are normally introduced to infants during weaning. Infant’s parents or guardians recorded how often the infant consumed each of the eleven foods. The questionnaire also recorded if a participant had received treatment with antibiotics since the last visit, the reason for taking the antibiotic, the name of the antibiotic, as well as the length of time the participant had been taking the antibiotic. A household survey was also completed at the beginning of the trial to collect mother’s education level, drinking water source, household flooring type, and animals present in the household. Analysis of breastfeeding and formula feeding patterns, complementary feeding practices, and associations with nutritional status at 6-months old (i.e. baseline) were previously reported for Nicaragua^88^. Monthly visits to the Mali community health clinic provided monitoring for malnutrition and severe adverse events; no adverse events were reported in the rice bran intervention group. There was one participant death reported in the control group (respiratory infection) and another withdrew to receive malnutrition treatment in the second month of the study. Diarrheal episodes were recorded, and a sample was collected in both countries using the same protocol.

### Stool analysis for EED markers

Stool biomarkers were selected to report gut inflammation and epithelial integrity as indicators of EED. These included neopterin (NEO), myeloperoxidase (MPO), calprotectin, (CAL) and alpha-1 antitrypsin (AAT)^89^. Suspended stool samples from 6, 8, and 12-month collections were centrifuged at 3,000 RPM to remove debris, following agitation, and the remaining supernatant was used for Enzyme-Linked-Immunosorbant-Assay (ELISA) determination of EED biomarker concentrations. Laboratory analysis protocols included in commercial kits were followed. Concentrations of CAL were determined at a 1:360 final dilution factor (Eagle Biosciences- Nashua, NH. Ref: CAL35-K01). Samples were diluted to 1:100 for determination of NEO concentrations (GenWay Biotech Inc- San Diego, CA, USA). MPO concentrations were determined at a 1:500 dilution factor (Immundiagnostik AG- Bensheim, Germany). Samples were diluted to 1:12,500 for determination of AAT concentrations (Immuchrom GMBH- Heppenheim, Germany), and dilution factors accounted for stool suspension ratios (20-fold). Final concentrations were determined from averages of three replicate assays and duplicate optical density readings, and interpolated using Graphpad 6.0 according to standards measured on each 96-well plate.

### Stool microbiota analysis for Nicaragua and Mali

The infant stool was collected at 6, 8 and 12 months of age from diapers and placed in a 1:19 ratio with Phosphate Buffered Saline + Glycerol solution. Diarrhea samples were collected using the same protocol. Suspended stool samples were vortexed before centrifuging at 3000 RPM to separate the stool debris. The remaining supernatant was used for Enzyme-Linked-Immunosorbant-Assay (ELISA) determination of EED biomarkers whereas; DNA was extracted for 16S microbial analysis from the stool pellet. DNA extraction was conducted using MoBio PowerSoil Kit (Reference number 12888, MoBio Laboratories Inc., Solana Beach, CA). PCR amplification of 390 bp amplicons was done in 50 µl reaction using Fischer Hot Start Master Mix and EMP standard protocols^43–47^. SPRI magnetic beads were used to purify DNA, and flourimetric quantification of Sybr Green tags was used to confirm adequate concentration of DNA. The pooled library was created with 50 ng DNA per sample and quantified using Kapa Kit (Kapa Biosystems). The pooled library was run on Illumina-MiSeq with 15% PhiX mock library to reduce discrepancies in read clustering, using the Illumina V2 500 cycle kit (2 × 250/250 paired-end reads).

### Microbiota data processing and analysis

Sequence data were processed using mothur^90^ version 1.39.5 and using a custom pipeline that provides an adjustment on the developers’ standard operating procedure (SOP) for OTU calling and taxonomic classification of MiSeq data first presented in Kozich, *et al*.^91^. For alignment and classification within this SOP we used the SILVA database^92^ version 128. Clustering, for OTU identification, was performed using VSEARCH using the distance based greedy clustering (DGC) option as implanted in mothur and utilizing 0.97 sequence similarity cutoff. We also used a cutoff of one read that was subtracted from all OTU read counts to guard against overestimation of sample richness. Rarefaction curves were generated using the package vegan^93^ as implemented in R version 3.4.4^94^ to assess diversity and suitability of depth of coverage per sample. The resulting OTU table was utilized in further data analyses as follows.

Exploring the data: Bar-graphs for relative abundance data based on the resulting OTU table were generated using the ggplot2^95^ package in R. These plots were generated for the data at the genus and the family levels and meant to describe the microbial community structure per sampled infant and per time point under each of the treatment levels.

Data were normalized using cumulative sum scaling (CSS)^96^ prior to beta diversity and log-fold change analyses. Nonmetric Multidimensional Scaling (NMDS)^97^ was used on the OTU level to assess possible trends and clustering in the microbial community structure comparing the two countries, the treatment conditions and the two time points, using the vegan package and utilizing Bray-Curtis dissimilarity^97^. Data were separated per country and the metagenomeSeq.^98^ package in R^94^ was used to fit a zero inflated normal (ZIN) model to test for log-fold change differences between the rice bran treatment and control per age group. Benjamini and Hochberg’s^99^ false discovery rate (FDR) method was used to correct for multiple testing and compute the adjusted p-values used to determine significance of differences in the log-fold change of OTU abundance.

### Stool metabolomics analysis

Stool samples were sent to Metabolon Inc. (Durham, NC, USA) for non-targeted metabolite profiling via ultrahigh-performance liquid chromatography tandem mass spectrometry (UPLC-MS/MS). All samples were accessioned into the Metabolon Library Information Management Systems (LIMS) and prepared using the automated MicroLab Star® system (Hamilton Company, Switzerland). Eight to ten recovery standards were added prior to the first step in the extraction process for quality control purposes. Extraction was performed using 80% ice-cold methanol under vigorous shaking for 2 min (Glen Mills GenoGrinder 2000) followed by centrifugation to remove protein, dissociate small molecules bound to protein or trapped in the precipitated protein matrix. Each stool extract was divided into five fractions: two for analysis by two separate reverse phase UPLC-MS/MS methods with positive ion mode electrospray ionization, 1 for analysis by reverse phase UPLC-MS/MS methods with negative ion mode electrospray ionization, 1 for hydrophilic interaction liquid chromatography UPLC-MS/MS with negative ion mode electrospray ionization, and 1 sample for backup. All samples were placed briefly on Concentration Evaporator (TurboVap® Zymark) to remove organic solvent. UPLC-MS/MS methods utilized a Waters ACQUITY ultra-performance liquid chromatography and a Thermo Scientific Q-Exactive high resolution/accurate mass spectrometer interfaced with a heated electrospray ionization (HESI-II) source and Orbitrap mass analyzer operated at 35,000 mass resolution. Raw data was extracted, peak-identified and processed for quality control using Metabolon’s hardware and software.

### Statistical analysis

Statistical analyses for anthropometric measures (length, weight, LAZ, WAZ, and WLZ) and stool EED biomarkers were completed using SAS 9.4 (Cary, NC, USA). The sample size was calculated for achieving greater than 85% power and based on expected changes in selected stool metabolites following dietary rice bran consumption for one month^27^. Normality was evaluated by visual inspection. For anthropometric variables, two-sample t-tests were used to compare means for the 2 treatment groups (rice bran and control) separately at birth and 6 months (prior to start of treatment). A repeated measures analysis was performed for each response variable separately using SAS Proc Mixed. Specifically, treatment (rice bran or control) and age (6, 8 or 12 months), and treatment-age interaction were included in the model as fixed effects. The participant was included as a random effect to account for repeated measures. At each age, treatment groups were compared using contrasts of the model. A similar repeated measures analysis was used EED biomarkers, but log transformation was used to satisfy model assumptions. For stool metabolites, Welch’s two-sample t-test was used to analyze statistical significance between groups’ stool metabolites, after participating in the 6-month dietary trial. A p-value of ≤ 0.05 was used for statistical significance. An estimated false discovery rate (q-value) was calculated to account for the multiple comparisons across metabolites that are typical of metabolomic-based studies.

### One sentence summary

Dietary rice bran supplementation during infant weaning from 6–12 months of age improved growth outcomes and supported metabolism by the gut microbiota.

## Supplementary information

Supplementary Figure 1 and Tables 1-5

## Data Availability

*16S* sequence data were submitted to the National Center for Biotechnology Information SRA under accession no. (SRP159269) and Bio-project (PRJNA488807).
