# Supplementary material for: Rice bran supplementation modulates growth, microbiota and metabolome in weaning infants: a clinical trial in Nicaragua and Mali
Source: Sci Rep. 2019 Sep 26;9:13919. doi: 10.1038/s41598-019-50344-4 (PMC6763478; doi:10.1038/s41598-019-50344-4)
Supplement: Supplementary file 1 — Supplementary Figure 1 and Tables 1-5 [file 41598_2019_50344_MOESM1_ESM.pdf]

Title:

Rice bran supplementation modulates growth, microbiota and metabolome in weaning infants: a clinical trial in Nicaragua and Mali

Authors and Affiliations:

Luis E. Zambrana[1,2], Starin McKeen[1], Hend Ibrahim[1,3], Iman Zarei[1], Erica C. Borresen[1], Lassina Doumbia[4], Abdoulaye Bore[4], Alima Cissoko[4], Seydou Douyon[4], Karim Kone[4], Johann Perez[2], Claudia Perez[2], Ann Hess[5], Zaid Abdo[6], Lansana Sangare[4], Ababacar Maiga[4], Sylvia Becker-Dreps[7], Lijuan Yuan[8], Ousmane Koita[4*], Samuel Vilchez[2*], & Elizabeth P. Ryan[1]*

Fig. S1. Overview of study design for both Nicaragua & Mali cohorts. A diagram of the eligibility criteria, intervention, participant information, and analysis of samples.

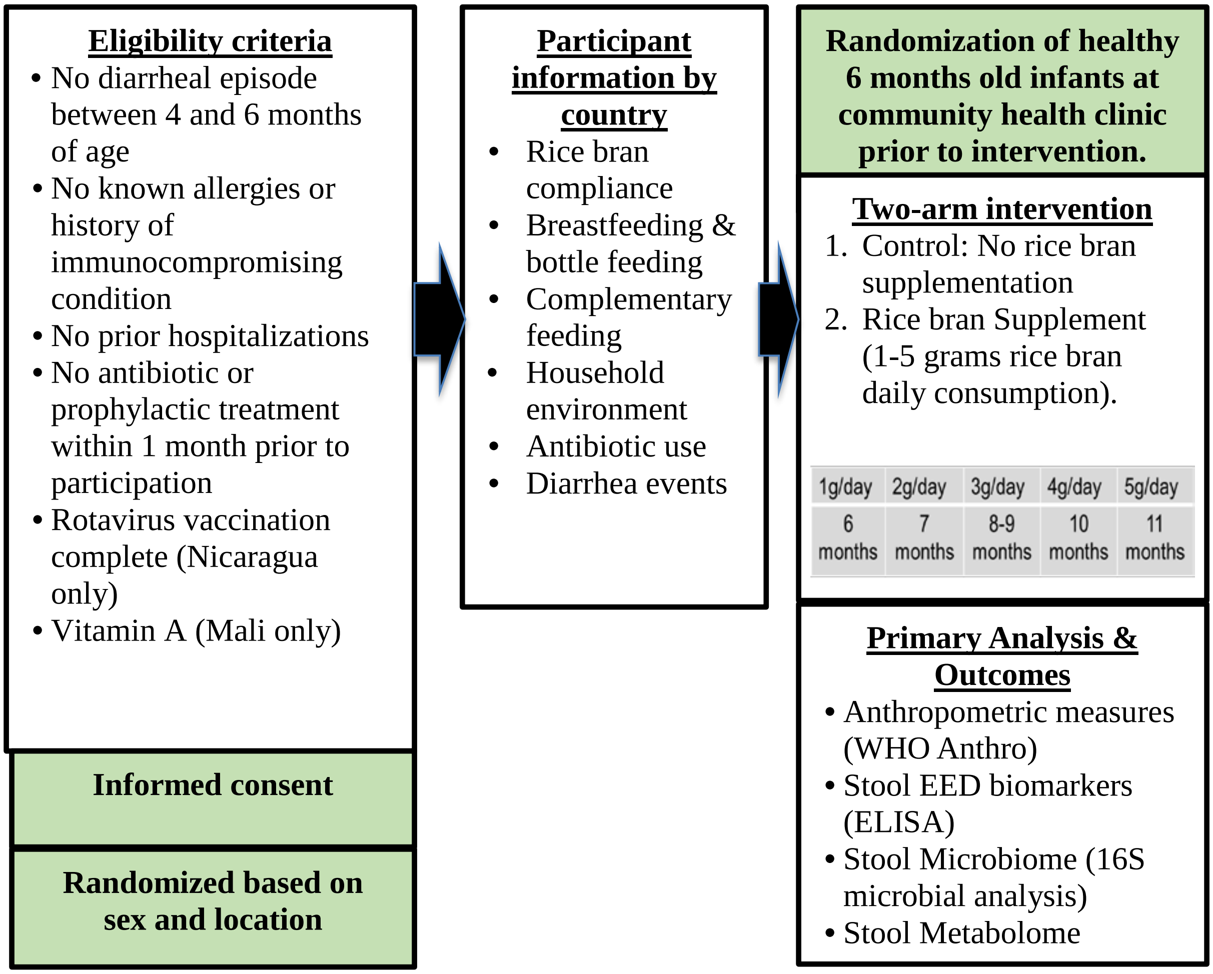

Title:

Rice bran supplementation modulates growth, microbiota and metabolome in weaning infants: a clinical trial in Nicaragua and Mali

Authors and Affiliations:

Luis E. Zambrana[1,2], Starin McKeen[1], Hend Ibrahim[1,3], Iman Zarei[1], Erica C. Borresen[1], Lassina Doumbia[4], Abdoulaye Bore[4], Alima Cissoko[4], Seydou Douyon[4], Karim Kone[4], Johann Perez[2], Claudia Perez[2], Ann Hess[5], Zaid Abdo[6], Lansana Sangare[4], Ababacar Maiga[4], Sylvia Becker-Dreps[7], Lijuan Yuan[8], Ousmane Koita[4*], Samuel Vilchez[2*], & Elizabeth P. Ryan[1]*

Table S1. The alpha diversity indices for gut microbial communities analyzed from Nicaraguan and Malian Infants at 8 and 12 months of age (data shown for all control and rice bran infants).

**Nicaragua_Control**								
**SampleID**	**country**	**group**	**sex**	**month**	**Observed**	**Shannon**	**InvSimpson**	**Richness**
COSU00591	Nicaraguan	Control	F	8	118	2.35	6.09	66.75
COSU00592	Nicaraguan	Control	F	8	134	2.39	7.23	78.37
COSU00594	Nicaraguan	Control	F	8	293	1.95	3.83	86.18
COSU00596	Nicaraguan	Control	F	8	173	2.40	4.75	110.96
COSU00603	Nicaraguan	Control	F	8	116	2.73	8.18	86.90
COSU00606	Nicaraguan	Control	F	8	95	2.22	6.27	53.84
COSU00608	Nicaraguan	Control	F	8	105	2.67	10.41	73.89
COSU00610	Nicaraguan	Control	F	8	114	1.88	3.72	81.72
COSU00611	Nicaraguan	Control	F	8	72	1.85	4.22	51.73
COSU00593	Nicaraguan	Control	M	8	96	2.16	4.90	56.81
COSU00595	Nicaraguan	Control	M	8	132	2.21	4.02	78.70
COSU00597	Nicaraguan	Control	M	8	140	3.11	13.28	95.60
COSU00598	Nicaraguan	Control	M	8	89	1.92	4.22	57.26
COSU00599	Nicaraguan	Control	M	8	94	1.93	4.48	52.51
COSU00600	Nicaraguan	Control	M	8	77	1.95	5.09	52.17
COSU00601	Nicaraguan	Control	M	8	100	2.51	7.08	83.89
COSU00602	Nicaraguan	Control	M	8	92	2.21	5.32	68.75
COSU00604	Nicaraguan	Control	M	8	98	2.26	5.20	83.26
COSU00605	Nicaraguan	Control	M	8	132	2.49	6.41	103.44
COSU00607	Nicaraguan	Control	M	8	178	2.64	6.48	115.70
COSU00609	Nicaraguan	Control	M	8	105	1.97	3.97	58.00
COSU00612	Nicaraguan	Control	M	8	96	1.86	3.57	58.24
COSU00635	Nicaraguan	Control	F	12	156	2.52	7.02	88.29

COSU00636	Nicaraguan	Control	F	12	99	1.79	2.81	72.23
COSU00638	Nicaraguan	Control	F	12	139	2.22	5.89	80.13
COSU00640	Nicaraguan	Control	F	12	165	2.69	7.34	114.71
COSU00646	Nicaraguan	Control	F	12	194	2.53	5.04	117.05
COSU00649	Nicaraguan	Control	F	12	115	1.88	3.15	70.62
COSU00651	Nicaraguan	Control	F	12	141	2.88	9.77	93.71
COSU00653	Nicaraguan	Control	F	12	116	2.91	10.95	88.02
COSU00654	Nicaraguan	Control	F	12	75	1.73	3.20	54.28
COSU00639	Nicaraguan	Control	M	12	157	2.89	9.89	107.53
COSU00641	Nicaraguan	Control	M	12	160	2.70	6.19	115.84
COSU00643	Nicaraguan	Control	M	12	96	2.34	5.54	61.45
COSU00644	Nicaraguan	Control	M	12	103	2.39	6.27	79.01
COSU00645	Nicaraguan	Control	M	12	103	2.41	6.58	78.79
COSU00647	Nicaraguan	Control	M	12	231	3.76	26.09	168.33
COSU00648	Nicaraguan	Control	M	12	136	2.59	9.13	86.78
COSU00650	Nicaraguan	Control	M	12	181	2.88	5.64	124.09
COSU00652	Nicaraguan	Control	M	12	151	3.03	10.07	91.66
COSU00655	Nicaraguan	Control	M	12	104	2.14	3.88	65.58
**Nicaragua_Rice bran**								
**SampleID**	**country**	**group**	**sex**	**month**	**Observed**	**Shannon**	**InvSimpson**	**Richness**
COSU00613	Nicaraguan	Rice_bran	F	8	204	2.70	7.91	115.86
COSU00614	Nicaraguan	Rice_bran	F	8	150	2.54	8.62	94.77
COSU00616	Nicaraguan	Rice_bran	F	8	78	1.40	2.44	50.26
COSU00620	Nicaraguan	Rice_bran	F	8	192	2.59	7.26	99.33
COSU00621	Nicaraguan	Rice_bran	F	8	62	1.93	4.98	50.53
COSU00622	Nicaraguan	Rice_bran	F	8	140	1.56	2.92	69.77
COSU00623	Nicaraguan	Rice_bran	F	8	152	2.22	4.02	91.98
COSU00629	Nicaraguan	Rice_bran	F	8	162	2.76	8.76	110.76
COSU00631	Nicaraguan	Rice_bran	F	8	206	2.08	3.71	103.54
COSU00634	Nicaraguan	Rice_bran	F	8	123	2.37	7.71	69.00
COSU00615	Nicaraguan	Rice_bran	M	8	102	1.99	3.51	64.13
COSU00617	Nicaraguan	Rice_bran	M	8	115	2.55	7.11	80.05
COSU00618	Nicaraguan	Rice_bran	M	8	119	2.13	5.82	75.76
COSU00619	Nicaraguan	Rice_bran	M	8	167	2.11	3.98	83.64
COSU00624	Nicaraguan	Rice_bran	M	8	177	2.56	8.08	92.85
COSU00625	Nicaraguan	Rice_bran	M	8	54	1.59	3.41	39.47
COSU00626	Nicaraguan	Rice_bran	M	8	351	3.20	12.16	187.50
COSU00627	Nicaraguan	Rice_bran	M	8	104	0.83	1.50	48.07
COSU00628	Nicaraguan	Rice_bran	M	8	710	2.11	4.92	97.68

COSU00630	Nicaraguan	Rice_bran	M	8	213	2.64	7.07	102.74
COSU00632	Nicaraguan	Rice_bran	M	8	72	1.36	2.64	45.41
COSU00633	Nicaraguan	Rice_bran	M	8	210	2.66	8.89	109.11
COSU00656	Nicaraguan	Rice_bran	F	12	154	2.35	6.15	91.10
COSU00657	Nicaraguan	Rice_bran	F	12	225	3.01	9.53	134.07
COSU00659	Nicaraguan	Rice_bran	F	12	138	2.62	8.69	90.78
COSU00663	Nicaraguan	Rice_bran	F	12	203	3.13	11.36	126.47
COSU00664	Nicaraguan	Rice_bran	F	12	137	2.02	5.32	83.94
COSU00665	Nicaraguan	Rice_bran	F	12	174	2.42	5.25	91.34
COSU00666	Nicaraguan	Rice_bran	F	12	118	2.09	4.46	69.68
COSU00672	Nicaraguan	Rice_bran	F	12	247	3.21	15.51	132.12
COSU00674	Nicaraguan	Rice_bran	F	12	96	1.04	1.80	47.66
COSU00677	Nicaraguan	Rice_bran	F	12	163	2.39	5.78	95.68
COSU00678	Nicaraguan	Rice_bran	F	12	210	2.32	5.87	95.95
COSU00658	Nicaraguan	Rice_bran	M	12	100	1.72	3.01	61.75
COSU00660	Nicaraguan	Rice_bran	M	12	185	2.94	9.95	127.45
COSU00661	Nicaraguan	Rice_bran	M	12	163	2.47	7.35	99.53
COSU00662	Nicaraguan	Rice_bran	M	12	121	2.24	6.16	79.40
COSU00667	Nicaraguan	Rice_bran	M	12	256	2.81	6.65	147.79
COSU00668	Nicaraguan	Rice_bran	M	12	117	2.14	4.10	81.01
COSU00669	Nicaraguan	Rice_bran	M	12	248	2.89	9.92	133.17
COSU00670	Nicaraguan	Rice_bran	M	12	281	2.52	5.57	128.36
COSU00671	Nicaraguan	Rice_bran	M	12	207	2.49	7.50	109.91
COSU00673	Nicaraguan	Rice_bran	M	12	207	2.55	7.16	101.09
COSU00675	Nicaraguan	Rice_bran	M	12	108	1.78	3.81	64.76
COSU00676	Nicaraguan	Rice_bran	M	12	198	2.77	10.24	103.28
**Mali_Control**								
**SampleID**	**country**	**group**	**sex**	**month**	**Observed**	**Shannon**	**InvSimpson**	**Richness**
COSU00720	Malian	Control	F	8	76	1.66	3.28	49.63
COSU00722	Malian	Control	F	8	53	1.64	2.60	46.01
COSU00723	Malian	Control	F	8	71	0.49	1.18	32.87
COSU00724	Malian	Control	F	8	44	0.97	1.57	35.66
COSU00728	Malian	Control	F	8	67	1.73	2.71	59.74
COSU00730	Malian	Control	F	8	78	1.50	2.20	52.32
COSU00734	Malian	Control	F	8	52	1.67	4.22	42.21
COSU00739	Malian	Control	F	8	39	0.75	1.41	28.54
COSU00740	Malian	Control	F	8	103	1.93	3.01	63.90
COSU00721	Malian	Control	M	8	54	1.69	3.47	49.76
COSU00725	Malian	Control	M	8	51	1.81	3.50	45.99

COSU00726	Malian	Control	M	8	137	3.09	14.13	107.16
COSU00727	Malian	Control	M	8	80	1.38	1.94	54.51
COSU00729	Malian	Control	M	8	53	1.14	1.69	44.37
COSU00731	Malian	Control	M	8	66	1.59	2.39	55.52
COSU00732	Malian	Control	M	8	96	1.60	2.31	58.47
COSU00733	Malian	Control	M	8	104	1.76	3.28	66.50
COSU00735	Malian	Control	M	8	97	1.93	3.56	56.55
COSU00736	Malian	Control	M	8	145	2.13	3.63	84.18
COSU00737	Malian	Control	M	8	41	1.09	2.01	28.40
COSU00738	Malian	Control	M	8	122	2.14	3.72	78.20
COSU00763	Malian	Control	F	12	47	1.89	3.92	44.70
COSU00765	Malian	Control	F	12	102	2.11	4.23	71.04
COSU00766	Malian	Control	F	12	109	2.29	4.14	78.86
COSU00767	Malian	Control	F	12	75	1.94	3.99	57.87
COSU00772	Malian	Control	F	12	81	1.98	3.33	56.28
COSU00774	Malian	Control	F	12	74	1.88	4.09	58.95
COSU00778	Malian	Control	F	12	115	2.52	8.52	84.21
COSU00782	Malian	Control	F	12	71	1.62	2.78	66.42
COSU00783	Malian	Control	F	12	195	2.35	5.48	117.78
COSU00784	Malian	Control	F	12	108	1.89	3.16	78.65
COSU00785	Malian	Control	F	12	187	2.65	6.77	118.63
COSU00764	Malian	Control	M	12	80	2.08	4.64	60.53
COSU00768	Malian	Control	M	12	104	2.49	6.19	83.79
COSU00770	Malian	Control	M	12	132	2.15	4.42	88.75
COSU00771	Malian	Control	M	12	100	2.35	6.71	70.71
COSU00773	Malian	Control	M	12	90	1.20	1.73	60.06
COSU00775	Malian	Control	M	12	64	1.66	3.28	49.08
COSU00776	Malian	Control	M	12	112	2.29	5.06	78.40
COSU00777	Malian	Control	M	12	131	2.49	8.19	84.32
COSU00779	Malian	Control	M	12	149	2.61	6.23	106.79
COSU00780	Malian	Control	M	12	131	1.92	3.12	92.67
COSU00781	Malian	Control	M	12	141	2.20	5.19	77.19
**Mali_Rice bran**								
**SampleID**	**country**	**group**	**sex**	**month**	**Observed**	**Shannon**	**InvSimpson**	**Richness**
COSU00744	Malian	Rice_bran	F	8	44	1.03	1.68	32.95
COSU00745	Malian	Rice_bran	F	8	93	1.87	3.79	77.37
COSU00746	Malian	Rice_bran	F	8	115	1.64	2.29	84.78
COSU00747	Malian	Rice_bran	F	8	48	1.36	2.02	37.41
COSU00749	Malian	Rice_bran	F	8	78	1.91	4.20	56.14

COSU00750	Malian	Rice_bran	F	8	92	2.25	3.93	72.36
COSU00755	Malian	Rice_bran	F	8	62	2.16	5.32	53.12
COSU00756	Malian	Rice_bran	F	8	111	2.40	7.05	74.61
COSU00758	Malian	Rice_bran	F	8	44	0.92	1.49	32.33
COSU00759	Malian	Rice_bran	F	8	108	1.66	2.29	65.16
COSU00741	Malian	Rice_bran	M	8	70	1.90	4.07	48.68
COSU00742	Malian	Rice_bran	M	8	51	1.55	2.72	38.98
COSU00748	Malian	Rice_bran	M	8	85	1.96	3.17	67.81
COSU00751	Malian	Rice_bran	M	8	60	1.23	1.98	49.58
COSU00752	Malian	Rice_bran	M	8	60	1.60	2.56	48.05
COSU00753	Malian	Rice_bran	M	8	62	1.49	2.07	49.66
COSU00754	Malian	Rice_bran	M	8	107	2.22	5.14	75.16
COSU00757	Malian	Rice_bran	M	8	156	2.07	5.11	73.08
COSU00760	Malian	Rice_bran	M	8	47	2.12	5.93	41.35
COSU00761	Malian	Rice_bran	M	8	96	1.99	3.09	72.28
COSU00789	Malian	Rice_bran	F	12	95	2.09	4.29	69.27
COSU00790	Malian	Rice_bran	F	12	99	2.08	4.51	74.15
COSU00791	Malian	Rice_bran	F	12	384	2.21	4.85	96.42
COSU00792	Malian	Rice_bran	F	12	110	2.03	2.98	88.57
COSU00793	Malian	Rice_bran	F	12	39	1.23	1.81	38.25
COSU00795	Malian	Rice_bran	F	12	68	1.46	2.83	53.32
COSU00796	Malian	Rice_bran	F	12	91	2.13	4.37	70.60
COSU00801	Malian	Rice_bran	F	12	193	2.18	3.90	82.71
COSU00802	Malian	Rice_bran	F	12	146	2.74	8.18	102.02
COSU00804	Malian	Rice_bran	F	12	95	2.02	4.57	77.93
COSU00805	Malian	Rice_bran	F	12	122	1.91	3.52	69.79
COSU00806	Malian	Rice_bran	F	12	69	2.27	5.25	51.84
COSU00786	Malian	Rice_bran	M	12	77	2.27	5.81	53.66
COSU00787	Malian	Rice_bran	M	12	61	1.42	2.09	49.42
COSU00788	Malian	Rice_bran	M	12	80	2.25	6.47	56.76
COSU00794	Malian	Rice_bran	M	12	51	1.77	3.33	39.14
COSU00797	Malian	Rice_bran	M	12	118	2.17	3.87	93.88
COSU00798	Malian	Rice_bran	M	12	77	2.56	7.74	77.00
COSU00799	Malian	Rice_bran	M	12	103	2.56	6.19	80.41
COSU00800	Malian	Rice_bran	M	12	112	1.97	2.78	79.47
COSU00803	Malian	Rice_bran	M	12	67	1.70	3.46	50.94
COSU00807	Malian	Rice_bran	M	12	69	2.24	5.28	55.48
COSU00808	Malian	Rice_bran	M	12	134	2.34	4.93	97.35

26

Title:

Rice bran supplementation modulates growth, microbiota and metabolome in weaning infants: a clinical trial in Nicaragua and Mali

Authors and Affiliations:

Luis E. Zambrana[1,2], Starin McKeen[1], Hend Ibrahim[1,3], Iman Zarei[1], Erica C. Borresen[1], Lassina Doumbia[4], Abdoulaye Bore[4], Alima Cissoko[4], Seydou Douyon[4], Karim Kone[4], Johann Perez[2], Claudia Perez[2], Ann Hess[5], Zaid Abdo[6], Lansana Sangare[4], Ababacar Maiga[4], Sylvia Becker-Dreps[7], Lijuan Yuan[8], Ousmane Koita[4*], Samuel Vilchez[2*], & Elizabeth P. Ryan[1]*

Table S2. The differences in gut microbial communities from infants fed rice bran and control. Data presented as log-fold changes in the Nicaraguan microbiome at 8 and 12 months of age for rice bran and control groups.

**Nicaragua Rice bran vs Control at 8 months**					
**Phylum**	**Family**	**Genus**	**OTUs**	**logFC**	**adj.P.Val**
Actinobacteria	Bifidobacteriaceae	Bifidobacteriaceae_unclassified	Otu0314	2.04	1.4E-06
		Bifidobacteriaceae_unclassified	Otu0294	1.84	9.8E-06
		Bifidobacterium	Otu0208	1.61	2.2E-03
		Bifidobacterium	Otu0275	1.19	4.5E-03
		Bifidobacteriaceae_unclassified	Otu0183	1.29	5.8E-03
		Bifidobacteriaceae_unclassified	Otu0265	1.09	9.5E-03
		Bifidobacteriaceae_unclassified	Otu0272	1.23	1.2E-02
		Bifidobacteriaceae_unclassified	Otu0390	0.81	4.1E-02
Bacteroidetes	Prevotellaceae	Prevotella_9	Otu0121	3.57	1.4E-07
	Bacteroidaceae	Bacteroides	Otu0192	-3.08	2.3E-07
		Bacteroides	Otu0407	2.20	2.3E-07
		Bacteroides	Otu0321	2.34	3.2E-06
		Bacteroides	Otu0379	2.26	5.7E-06
		Bacteroides	Otu0266	2.89	9.8E-06
		Bacteroides	Otu0312	2.22	2.9E-05

	Bacteroides	Otu0433	2.01	5.0E-05
Prevotellaceae	Prevotella_9	Otu0396	1.82	8.6E-05
Bacteroidaceae	Bacteroides	Otu0517	1.50	9.7E-05
	Bacteroides	Otu0442	1.60	2.4E-04
	Bacteroides	Otu0254	1.28	2.7E-04
	Bacteroides	Otu0414	1.55	3.5E-04
	Bacteroides	Otu0292	1.89	3.8E-04
	Bacteroides	Otu0456	1.43	4.3E-04
	Bacteroides	Otu0129	2.59	4.3E-04
	Bacteroides	Otu0319	1.78	8.2E-04
	Bacteroides	Otu0358	1.90	9.0E-04
	Bacteroides	Otu0334	1.66	1.0E-03
	Bacteroides	Otu0437	1.35	1.3E-03
	Bacteroides	Otu0311	1.72	2.2E-03
	Bacteroides	Otu0051	3.50	2.8E-03
	Bacteroides	Otu0502	1.25	3.1E-03
	Bacteroides	Otu0270	1.83	3.3E-03
	Bacteroides	Otu0368	1.51	4.2E-03
	Bacteroides	Otu0428	1.18	4.6E-03
	Bacteroides	Otu0410	-1.24	5.1E-03
	Bacteroides	Otu0224	1.52	5.1E-03
Rikenellaceae	Alistipes	Otu0148	1.34	5.6E-03
Bacteroidaceae	Bacteroides	Otu0287	1.50	5.8E-03
	Bacteroides	Otu0169	1.90	6.1E-03
Porphyromonadaceae	Parabacteroides	Otu0086	-2.34	7.4E-03
Bacteroidaceae	Bacteroides	Otu0249	1.16	7.4E-03
	Bacteroides	Otu0487	1.06	7.6E-03
Prevotellaceae	Prevotella	Otu0426	-1.45	7.6E-03
Bacteroidaceae	Bacteroides	Otu0114	1.70	7.6E-03
	Bacteroides	Otu0530	-1.06	9.2E-03
	Bacteroides	Otu0274	1.51	1.1E-02
Prevotellaceae	Paraprevotella	Otu0189	1.41	1.2E-02
Bacteroidaceae	Bacteroides	Otu0295	1.31	1.2E-02

		Bacteroides	Otu0405	1.23	1.4E-02
		Bacteroides	Otu0322	1.44	1.4E-02
		Bacteroides	Otu0088	1.82	1.8E-02
		Bacteroides	Otu0170	1.67	2.1E-02
		Bacteroides	Otu0279	1.35	2.3E-02
		Bacteroides	Otu0283	1.29	2.7E-02
		Bacteroides	Otu0205	1.32	4.9E-02
Firmicutes	Veillonellaceae	Veillonella	Otu0245	3.94	4.8E-10
		Veillonella	Otu0289	4.47	7.6E-10
		Veillonella	Otu0241	3.10	1.4E-08
		Veillonella	Otu0253	4.21	1.4E-08
	Erysipelotrichaceae	Candidatus_Stoquefichus	Otu0341	3.42	1.4E-08
	Veillonellaceae	Veillonella	Otu0316	3.51	2.2E-08
	Lachnospiraceae	Lachnospiraceae_unclassified	Otu0280	5.84	4.0E-08
	Veillonellaceae	Veillonella	Otu0347	2.76	6.1E-08
		Veillonella	Otu0403	3.07	1.9E-07
		Veillonella	Otu0397	3.04	1.9E-07
	Lachnospiraceae	Anaerostipes	Otu0284	2.89	1.9E-07
	Veillonellaceae	Veillonella	Otu0165	3.00	2.7E-07
	Lachnospiraceae	Lachnospiraceae_ND3007_group	Otu0242	3.10	2.9E-07
	Acidaminococcaceae	Phascolarctobacterium	Otu0066	4.64	3.3E-07
	Lachnospiraceae	Lachnospiraceae_unclassified	Otu0157	3.19	1.2E-06
	Veillonellaceae	Megasphaera	Otu0258	2.18	1.4E-06
	Ruminococcaceae	Faecalibacterium	Otu0514	1.80	1.8E-06
	Veillonellaceae	Veillonella	Otu0298	2.84	3.2E-06
	Clostridiaceae_1	Clostridium_sensu_stricto_1	Otu0099	3.57	8.5E-06
	Lactobacillaceae	Lactobacillus	Otu0053	-3.85	1.1E-05
	Veillonellaceae	Megasphaera	Otu0350	1.59	2.0E-05
	Ruminococcaceae	Ruminococcus_2	Otu0175	-2.70	2.0E-05
		Flavonifractor	Otu0553	1.55	4.0E-05
		Ruminococcaceae_unclassified	Otu0374	1.49	5.2E-05
	Streptococcaceae	Streptococcus	Otu0201	1.57	6.1E-05
	Lachnospiraceae	Lachnospiraceae_unclassified	Otu0101	2.84	1.3E-04

	Veillonellaceae	Megasphaera	Otu0330	1.12	4.5E-04
		Allisonella	Otu0209	1.48	5.3E-04
	Lachnospiraceae	Lachnospiraceae_unclassified	Otu0297	-1.92	5.7E-04
	Clostridiaceae_1	Clostridium_sensu_stricto_1	Otu0211	2.06	8.6E-04
	Ruminococcaceae	Ruminococcaceae_unclassified	Otu0238	2.01	9.7E-04
	Erysipelotrichaceae	Holdemania	Otu0419	1.52	1.3E-03
	Lachnospiraceae	Anaerostipes	Otu0204	2.37	1.6E-03
	Clostridiaceae_1	Clostridium_sensu_stricto_1	Otu0191	-2.33	1.6E-03
	Peptostreptococcaceae	Peptostreptococcaceae_unclassified	Otu0124	-2.38	1.9E-03
	Lactobacillaceae	Lactobacillus	Otu0024	1.78	2.4E-03
	Ruminococcaceae	Oscillibacter	Otu0163	-2.49	2.9E-03
	Veillonellaceae	Megasphaera	Otu0421	1.03	2.9E-03
	Lachnospiraceae	Lachnospiraceae_unclassified	Otu0174	-2.27	3.3E-03
	Ruminococcaceae	Faecalibacterium	Otu0206	1.48	3.3E-03
	Lachnospiraceae	Roseburia	Otu0216	1.95	3.6E-03
		Tyzzerella_4	Otu0435	1.06	5.1E-03
		Blautia	Otu0070	2.51	5.6E-03
	Clostridiaceae_1	Sarcina	Otu0031	-2.89	6.1E-03
	Erysipelotrichaceae	Erysipelatoclostridium	Otu0038	2.07	8.0E-03
	Ruminococcaceae	Faecalibacterium	Otu0389	1.29	9.4E-03
	Clostridiaceae_1	Clostridium_sensu_stricto_1	Otu0118	1.92	1.2E-02
	Lachnospiraceae	Lachnospiraceae_ge	Otu0090	-2.20	1.4E-02
	Ruminococcaceae	Faecalibacterium	Otu0173	1.40	1.4E-02
	Veillonellaceae	Veillonellaceae_unclassified	Otu0056	2.15	2.9E-02
	Ruminococcaceae	Faecalibacterium	Otu0257	1.20	3.4E-02
	Veillonellaceae	Megasphaera	Otu0454	0.96	4.2E-02
	Family_XI	Gemella	Otu0255	0.76	4.2E-02
	Ruminococcaceae	Faecalibacterium	Otu0440	0.69	4.8E-02
Fusobacteria	Fusobacteriaceae	Fusobacterium	Otu0158	1.19	4.2E-02
Proteobacteria	Enterobacteriaceae	Enterobacteriaceae_unclassified	Otu0357	2.22	6.8E-07
		Enterobacteriaceae_unclassified	Otu0365	2.03	3.2E-06
		Enterobacteriaceae_unclassified	Otu0348	1.93	9.8E-06
		Enterobacteriaceae_unclassified	Otu0128	2.20	3.8E-05

	Desulfovibrionaceae	Desulfovibrio	Otu0164	2.34	1.7E-04
	Moraxellaceae	Acinetobacter	Otu0375	-1.20	2.0E-03
	Enterobacteriaceae	Morganella	Otu0097	2.31	4.5E-03
	Succinivibrionaceae	Succinivibrionaceae_unclassified	Otu0042	-2.59	1.4E-02
	Enterobacteriaceae	Proteus	Otu0113	1.10	1.4E-02
		Enterobacteriaceae_unclassified	Otu0268	1.37	2.1E-02
**Nicaragua Rice bran vs Control at 12 months**					
**Phylum**	**Family**	**Genus**	**OTUs**	**logFC**	**adj.P.Val**
Actinobacteria	Bifidobacteriaceae	Bifidobacterium	Otu0332	2.61	1.4E-05
	Coriobacteriaceae	Atopobium	Otu0234	2.14	3.0E-05
	Bifidobacteriaceae	Bifidobacteriaceae_unclassified	Otu0314	1.35	6.5E-04
		Bifidobacterium	Otu0394	1.30	9.6E-03
		Bifidobacterium	Otu0275	1.08	2.5E-02
Bacteroidetes	Prevotellaceae	Paraprevotella	Otu0189	6.21	4.3E-08
	Bacteroidaceae	Bacteroides	Otu0146	5.73	1.4E-07
		Bacteroides	Otu0487	2.34	7.5E-06
		Bacteroides	Otu0517	1.90	2.8E-05
		Bacteroides	Otu0088	3.41	1.4E-04
		Bacteroides	Otu0287	2.28	5.0E-04
	Rikenellaceae	Alistipes	Otu0148	1.92	5.3E-04
	Prevotellaceae	Prevotella	Otu0426	2.04	9.3E-04
	Bacteroidaceae	Bacteroides	Otu0433	1.77	1.1E-03
	Rikenellaceae	Alistipes	Otu0271	1.74	1.1E-03
	Prevotellaceae	Prevotella_9	Otu0396	1.34	4.0E-03
	Bacteroidaceae	Bacteroides	Otu0205	2.29	7.8E-03
		Bacteroides	Otu0456	1.26	8.0E-03
		Bacteroides	Otu0334	1.51	8.7E-03
		Bacteroides	Otu0321	1.30	1.0E-02
		Bacteroides	Otu0410	1.21	1.2E-02
		Bacteroides	Otu0292	1.48	1.9E-02
		Bacteroides	Otu0530	1.19	1.9E-02
		Bacteroides	Otu0169	1.96	2.0E-02
		Bacteroides	Otu0442	1.02	3.3E-02

		Bacteroides	Otu0224	1.40	3.8E-02
Firmicutes	Veillonellaceae	Allisonella	Otu0209	-4.07	1.6E-08
	Ruminococcaceae	Faecalibacterium	Otu0206	4.38	1.6E-08
	Acidaminococcaceae	Phascolarctobacterium	Otu0066	6.13	1.6E-08
	Veillonellaceae	Veillonella	Otu0253	3.35	3.3E-07
		Megasphaera	Otu0421	2.49	3.3E-07
		Veillonella	Otu0241	2.42	1.6E-06
		Megasphaera	Otu0330	1.79	6.1E-06
		Megasphaera	Otu0438	2.74	7.1E-06
		Megasphaera	Otu0350	1.97	7.8E-06
	Lachnospiraceae	Lachnospiraceae_unclassified	Otu0280	3.26	1.4E-05
		Eisenbergiella	Otu0156	-2.72	2.4E-05
	Veillonellaceae	Megasphaera	Otu0258	1.76	8.0E-05
		Veillonella	Otu0165	2.19	8.0E-05
	Ruminococcaceae	Ruminococcaceae_unclassified	Otu0374	-1.54	2.8E-04
	Lachnospiraceae	Lachnospiraceae_ND3007_group	Otu0242	-2.02	2.9E-04
		Lachnospiraceae_unclassified	Otu0353	-1.91	5.6E-04
	Family_XI	Gemella	Otu0255	1.95	6.5E-04
	Lactobacillaceae	Lactobacillus	Otu0293	1.87	1.0E-03
	Streptococcaceae	Streptococcus	Otu0201	1.26	1.5E-03
	Lachnospiraceae	Tyzzerella_4	Otu0435	-1.39	3.0E-03
	Veillonellaceae	Megasphaera	Otu0340	1.57	4.2E-03
		Dialister	Otu0151	2.35	4.6E-03
	Ruminococcaceae	Faecalibacterium	Otu0514	1.06	4.8E-03
		Faecalibacterium	Otu0257	1.79	5.8E-03
	Carnobacteriaceae	Granulicatella	Otu0364	1.49	8.7E-03
	Lachnospiraceae	Lachnoclostridium	Otu0162	2.33	1.2E-02
		Sellimonas	Otu0098	2.13	1.4E-02
	Lactobacillaceae	Lactobacillus	Otu0110	0.87	1.6E-02
	Ruminococcaceae	Butyricicoccus	Otu0250	1.50	1.9E-02
	Veillonellaceae	Megasphaera	Otu0178	1.25	1.9E-02
	Clostridiaceae_1	Clostridium_sensu_stricto_1	Otu0259	1.13	2.2E-02
	Erysipelotrichaceae	Erysipelotrichaceae_UCG-003	Otu0116	-2.55	2.2E-02

	Lachnospiraceae	Dorea	Otu0085	1.82	2.5E-02
		Blautia	Otu0168	-2.00	3.6E-02
	Ruminococcaceae	Flavonifractor	Otu0328	-1.33	3.8E-02
	Lachnospiraceae	Lachnospiraceae_unclassified	Otu0108	2.20	3.8E-02
	Lactobacillaceae	Lactobacillus	Otu0053	1.29	3.9E-02
	Lachnospiraceae	Lachnospiraceae_unclassified	Otu0157	-1.48	4.1E-02
	Veillonellaceae	Megasphaera	Otu0454	1.17	4.2E-02
		Veillonellaceae_unclassified	Otu0056	2.29	4.9E-02
	Lachnospiraceae	Anaerostipes	Otu0284	-0.94	4.9E-02
		Lachnospiraceae_unclassified	Otu0297	1.07	4.9E-02
Proteobacteria	Pseudomonadaceae	Pseudomonas	Otu0306	2.05	2.4E-05
	Enterobacteriaceae	Proteus	Otu0113	-2.77	9.1E-05
		Enterobacteriaceae_unclassified	Otu0268	2.09	4.8E-03
	Campylobacteraceae	Campylobacter	Otu0064	1.87	9.7E-03

Title:

Rice bran supplementation modulates growth, microbiota and metabolome in weaning infants: a clinical trial in Nicaragua and Mali

Authors and Affiliations:

Luis E. Zambrana[1,2], Starin McKeen[1], Hend Ibrahim[1,3], Iman Zarei[1], Erica C. Borresen[1], Lassina Doumbia[4], Abdoulaye Bore[4], Alima Cissoko[4], Seydou Douyon[4], Karim Kone[4], Johann Perez[2], Claudia Perez[2], Ann Hess[5], Zaid Abdo[6], Lansana Sangare[4], Ababacar Maiga[4], Sylvia Becker-Dreps[7], Lijuan Yuan[8], Ousmane Koita[4*], Samuel Vilchez[2*], & Elizabeth P. Ryan[1]*

Table S3. The differences in gut microbial communities from infants fed rice bran and control. Data presented as log-fold changes in the Mali microbiome at 8 and 12 months of age for rice bran and control groups.

**Mali Rice bran vs Control at 8 months**					
**Phylum**	**Family**	**Genus**	**OTUs**	**logFC**	**adj.P.Val**
Actinobacteria	Coriobacteriaceae	Coriobacteriaceae_unclassified	Otu0080	5.13	4.1E-07
		Eggerthella	Otu0142	-2.35	1.1E-06
	Bifidobacteriaceae	Bifidobacterium	Otu0275	-1.83	3.9E-06
	Corynebacteriaceae	Corynebacterium_1	Otu0581	-1.48	5.0E-03
	Bifidobacteriaceae	Bifidobacteriaceae_unclassified	Otu0265	-1.09	1.0E-02
	Actinomycetaceae	Actinomyces	Otu0460	-1.05	2.7E-02
	Coriobacteriaceae	Senegalimassilia	Otu0115	-1.55	2.7E-02
Firmicutes	Lactobacillaceae	Lactobacillus	Otu0356	3.26	1.4E-09
	Peptostreptococcaceae	Terrisporobacter	Otu0247	3.08	9.1E-08
	Lactobacillaceae	Lactobacillus	Otu0120	5.51	2.0E-07
	Veillonellaceae	Megasphaera	Otu0178	-2.27	1.2E-03
	Lactobacillaceae	Lactobacillus	Otu0016	4.29	1.2E-03
	Ruminococcaceae	Ruminococcaceae_ge	Otu0075	-2.72	6.1E-03

	Lactobacillaceae	Lactobacillus	Otu0024	2.78	1.3E-02
	Lachnospiraceae	Lachnospiraceae_unclassified	Otu0010	-2.33	1.6E-02
	Clostridiaceae_1	Clostridium_sensu_stricto_1	Otu0099	-1.15	4.7E-02
	Family_XI	Gemella	Otu0255	-0.91	4.7E-02
Proteobacteria	Campylobacteraceae	Campylobacter	Otu0123	-2.22	3.1E-02
Verrucomicrobia	Verrucomicrobiaceae	Akkermansia	Otu0018	2.25	6.1E-03
**Mali Rice bran vs Control at 12 months**					
**Phylum**	**Family**	**Genus**	**OTUs**	**logFC**	**adj.P.Val**
Actinobacteria	Dietziaceae	Dietzia	Otu0550	-1.73	4.0E-05
	Bifidobacteriaceae	Bifidobacteriaceae_unclassified	Otu0265	-2.64	4.0E-05
		Bifidobacteriaceae_unclassified	Otu0278	1.54	3.2E-04
	Micrococcaceae	Kocuria	Otu0325	1.61	2.3E-03
	Bifidobacteriaceae	Bifidobacterium	Otu0275	-0.88	2.2E-02
		Bifidobacteriaceae_unclassified	Otu0390	-0.57	4.6E-02
Bacteroidetes	Prevotellaceae	Alloprevotella	Otu0068	3.63	3.5E-04
Firmicutes	Peptostreptococcaceae	Terrisporobacter	Otu0247	-2.18	7.0E-06
	Lachnospiraceae	Lachnospira	Otu0058	-2.83	2.9E-05
	Clostridiaceae_1	Clostridium_sensu_stricto_1	Otu0076	-3.64	4.0E-05
	Lachnospiraceae	Tyzzerella_4	Otu0026	-3.29	6.5E-05
	Veillonellaceae	Megasphaera	Otu0361	-3.55	3.2E-04
	Ruminococcaceae	Faecalibacterium	Otu0173	-1.63	2.1E-03
	Lachnospiraceae	Lachnospiraceae_unclassified	Otu0101	-1.66	7.0E-03
	Lactobacillaceae	Lactobacillus	Otu0053	2.70	9.8E-03
	Lachnospiraceae	Roseburia	Otu0047	-1.48	1.6E-02
	Veillonellaceae	Veillonella	Otu0152	1.99	1.6E-02
	Peptostreptococcaceae	Peptostreptococcaceae_unclassified	Otu0078	-1.51	1.6E-02
	Streptococcaceae	Streptococcus	Otu0479	1.05	1.9E-02
	Lactobacillaceae	Lactobacillus	Otu0024	2.18	2.0E-02
	Peptococcaceae	Peptococcus	Otu0351	-1.63	2.3E-02
	Ruminococcaceae	Faecalibacterium	Otu0257	-0.77	2.7E-02
Fusobacteria	Fusobacteriaceae	Fusobacterium	Otu0158	-1.81	8.4E-03
Proteobacteria	Enterobacteriaceae	Enterobacteriaceae_unclassified	Otu0304	-1.61	4.0E-05

		Enterobacteriaceae_unclassified	Otu0128	-1.62	1.2E-03
	Campylobacteraceae	Campylobacter	Otu0123	2.55	9.1E-03
	Neisseriaceae	Neisseria	Otu0318	1.42	3.1E-02
	Pasteurellaceae	Haemophilus	Otu0180	1.46	3.2E-02
Verrucomicrobia	Verrucomicrobiaceae	Akkermansia	Otu0018	-1.80	1.3E-02

Title:

Rice bran supplementation modulates growth, microbiota and metabolome in weaning infants: a clinical trial in Nicaragua and Mali

Authors and Affiliations:

Luis E. Zambrana[1,2], Starin McKeen[1], Hend Ibrahim[1,3], Iman Zarei[1], Erica C. Borresen[1], Lassina Doumbia[4], Abdoulaye Bore[4], Alima Cissoko[4], Seydou Douyon[4], Karim Kone[4], Johann Perez[2], Claudia Perez[2], Ann Hess[5], Zaid Abdo[6], Lansana Sangare[4], Ababacar Maiga[4], Sylvia Becker-Dreps[7], Lijuan Yuan[8], Ousmane Koita[4*], Samuel Vilchez[2*], & Elizabeth P. Ryan[1]*

Table S4. The OTUs with overlapping significance for response to rice bran when compared to control in both Mali and Nicaragua**.**

				mali8m		mali12m		nic8m		nic12m	
**Phylum**	**Family**	**Genus**	**OTUs**	**logFC**	**adj.P.Val**	**logFC**	**adj.P.Val**	**logFC**	**adj.P.Val**	**logFC**	**adj.P.Val**
Actinobacteria	Bifidobacteriaceae	Bifidobacterium	Otu0275	-1.83	3.9E-06	-0.88	0.02	1.19	0.004	1.08	0.025
Firmicutes	Veillonellaceae	Megasphaera	Otu0178	-2.27	1.2E-03					1.25	0.019
Actinobacteria	Bifidobacteriaceae	Bifidobacteriaceae_unclassified	Otu0265	-1.09	1.0E-02	-1.09	0.01	1.09	0.009		
Firmicutes	Family_XI	Gemella	Otu0255	-0.91	4.7E-02			0.76	0.042	1.95	0.001
Firmicutes	Lactobacillaceae	Lactobacillus	Otu0024	2.78	1.3E-02	2.18	0.02	1.78	0.002		
Proteobacteria	Enterobacteriaceae	Enterobacteriaceae_unclassified	Otu0128			-1.62	0.00	2.20	0.000		
Firmicutes	Ruminococcaceae	Faecalibacterium	Otu0173			-1.63	0.00	1.40	0.014		
Firmicutes	Lachnospiraceae	Lachnospiraceae_unclassified	Otu0101			-1.66	0.01	2.84	0.000		
Fusobacteria	Fusobacteriaceae	Fusobacterium	Otu0158			-1.81	0.01	1.19	0.042		
Firmicutes	Lactobacillaceae	Lactobacillus	Otu0053			2.70	0.01	-3.85	0.000	1.29	0.039
Firmicutes	Ruminococcaceae	Faecalibacterium	Otu0257			-0.77	0.03	1.20	0.034		
Actinobacteria	Bifidobacteriaceae	Bifidobacteriaceae_unclassified	Otu0390			-0.57	0.05	0.81	0.041		

Title:

Rice bran supplementation modulates growth, microbiota and metabolome in weaning infants: a clinical trial in Nicaragua and Mali

Authors and Affiliations:

Luis E. Zambrana[1,2], Starin McKeen[1], Hend Ibrahim[1,3], Iman Zarei[1], Erica C. Borresen[1], Lassina Doumbia[4], Abdoulaye Bore[4], Alima Cissoko[4], Seydou Douyon[4], Karim Kone[4], Johann Perez[2], Claudia Perez[2], Ann Hess[5], Zaid Abdo[6], Lansana Sangare[4], Ababacar Maiga[4], Sylvia Becker-Dreps[7], Lijuan Yuan[8], Ousmane Koita[4*], Samuel Vilchez[2*], & Elizabeth P. Ryan[1]*

Table S5. All of the stool metabolites identified from the stool metabolome, including those with unknown identity that had fold differences in the relative abundances between rice bran supplementation and control infants. Data are presented for both Nicaraguan and Malian infants at 8 months of age.

Chemical Class	Metabolic Pathways	Metabolites	HMDB	Nicaragua		Mali	
				Fold Differences	p-value	Fold Differences	p-value
Amino Acid	Glycine, Serine and Threonine Metabolism	glycine	HMDB00123	**0.66**	0.01	1.2	0.24
Amino Acid	Glycine, Serine and Threonine Metabolism	N-acetylglycine	HMDB00532	0.73	0.18	1.13	0.60
Amino Acid	Glycine, Serine and Threonine Metabolism	sarcosine	HMDB00271	0.99	0.98	0.69	0.45
Amino Acid	Glycine, Serine and Threonine Metabolism	dimethylglycine	HMDB00092	0.69	0.29	**0.42**	0.02
Amino Acid	Glycine, Serine and Threonine Metabolism	betaine	HMDB00043	0.67	0.38	0.74	0.53
Amino Acid	Glycine, Serine and Threonine Metabolism	serine	HMDB00187	0.87	0.48	1.1	0.63
Amino Acid	Glycine, Serine and Threonine Metabolism	N-acetylserine	HMDB02931	0.89	0.59	0.95	0.83
Amino Acid	Glycine, Serine and Threonine Metabolism	O-acetylserine	HMDB03011	1.05	0.34	**0.89**	0.06
Amino Acid	Glycine, Serine and Threonine Metabolism	2-methylserine		0.67	0.32	1.24	0.61

Amino Acid	Glycine, Serine and Threonine Metabolism	threonine	HMDB00167	0.8	0.32	1.11	0.64
Amino Acid	Glycine, Serine and Threonine Metabolism	N-acetylthreonine		0.9	0.80	0.72	0.46
Amino Acid	Glycine, Serine and Threonine Metabolism	allo-threonine	HMDB04041	1.13	0.77	0.86	0.73
Amino Acid	Glycine, Serine and Threonine Metabolism	homoserine	HMDB00719	0.77	0.55	0.64	0.34
Amino Acid	Glycine, Serine and Threonine Metabolism	O-acetylhomoserine		0.65	0.46	1.1	0.88
Amino Acid	Alanine and Aspartate Metabolism	alanine	HMDB00161	0.84	0.12	1.08	0.49
Amino Acid	Alanine and Aspartate Metabolism	N-acetylalanine	HMDB00766	0.88	0.49	1.07	0.75
Amino Acid	Alanine and Aspartate Metabolism	N-methylalanine	HMDB01906	1.13	0.77	0.66	0.34
Amino Acid	Alanine and Aspartate Metabolism	N-propionylalanine		1.25	0.62	0.6	0.28
Amino Acid	Alanine and Aspartate Metabolism	aspartate	HMDB00191	1.18	0.53	**1.66**	0.07
Amino Acid	Alanine and Aspartate Metabolism	N-acetylaspartate (NAA)	HMDB00812	0.95	0.90	0.96	0.92
Amino Acid	Alanine and Aspartate Metabolism	asparagine	HMDB00168	1.13	0.78	**2.16**	0.10
Amino Acid	Alanine and Aspartate Metabolism	N-acetylasparagine	HMDB06028	0.92	0.72	1.05	0.84
Amino Acid	Glutamate Metabolism	glutamate	HMDB00148	0.88	0.60	1.46	0.15
Amino Acid	Glutamate Metabolism	glutamine	HMDB00641	0.72	0.52	2.19	0.13
Amino Acid	Glutamate Metabolism	N-acetylglutamate	HMDB01138	0.95	0.85	1.44	0.21
Amino Acid	Glutamate Metabolism	N-acetylglutamine	HMDB06029	0.85	0.48	0.72	0.17
Amino Acid	Glutamate Metabolism	4-hydroxyglutamate	HMDB01344	0.8	0.57	1.04	0.91
Amino Acid	Glutamate Metabolism	gamma-carboxyglutamate	HMDB41900	**1.97**	0.08	0.81	0.61
Amino Acid	Glutamate Metabolism	glutamate, gamma-methyl ester	HMDB61715	0.99	0.98	1.3	0.29
Amino Acid	Glutamate Metabolism	pyroglutamine*		0.78	0.46	0.92	0.82
Amino Acid	Glutamate Metabolism	beta-citrylglutamate		0.95	0.92	0.49	0.18
Amino Acid	Glutamate Metabolism	gamma-aminobutyrate (GABA)	HMDB00112	0.78	0.61	1.61	0.35
Amino Acid	Glutamate Metabolism	carboxyethyl-GABA	HMDB02201	0.8	0.47	1.3	0.40
Amino Acid	Glutamate Metabolism	S-1-pyrroline-5-carboxylate	HMDB01301	0.84	0.31	0.98	0.92
Amino Acid	Glutamate Metabolism	propionylglutamine		1.18	0.58	0.75	0.35
Amino Acid	Glutamate Metabolism	butyrylglutamine/isobutyrylglutamine		1.12	0.79	0.9	0.81
Amino Acid	Glutamate Metabolism	succinylglutamine		0.86	0.65	0.82	0.59

Amino Acid	Glutamate Metabolism	valerylglutamine		0.75	0.54	0.96	0.93
Amino Acid	Histidine Metabolism	histidine	HMDB00177	0.8	0.45	0.68	0.20
Amino Acid	Histidine Metabolism	1-methylhistidine	HMDB00001	1.19	0.60	0.79	0.49
Amino Acid	Histidine Metabolism	3-methylhistidine	HMDB00479	0.64	0.25	**0.48**	0.07
Amino Acid	Histidine Metabolism	N-acetylhistidine	HMDB32055	1.28	0.40	1.27	0.45
Amino Acid	Histidine Metabolism	N-acetyl-1-methylhistidine*		0.87	0.74	0.89	0.79
Amino Acid	Histidine Metabolism	hydantoin-5-propionic acid	HMDB01212	0.98	0.94	1.19	0.62
Amino Acid	Histidine Metabolism	trans-urocanate	HMDB00301	0.86	0.77	1.79	0.30
Amino Acid	Histidine Metabolism	cis-urocanate	HMDB34174	0.73	0.28	0.91	0.75
Amino Acid	Histidine Metabolism	imidazole propionate	HMDB02271	0.72	0.47	1.07	0.89
Amino Acid	Histidine Metabolism	formiminoglutamate	HMDB00854	0.83	0.74	1.74	0.32
Amino Acid	Histidine Metabolism	imidazole lactate	HMDB02320	1.07	0.89	1.18	0.74
Amino Acid	Histidine Metabolism	carnosine	HMDB00033	**0.4**	0.08	0.72	0.55
Amino Acid	Histidine Metabolism	homocarnosine	HMDB00745	0.82	0.63	0.78	0.55
Amino Acid	Histidine Metabolism	N-acetylcarnosine	HMDB12881	0.75	0.44	0.87	0.73
Amino Acid	Histidine Metabolism	anserine	HMDB00194	0.57	0.23	0.83	0.71
Amino Acid	Histidine Metabolism	histamine	HMDB00870	1.05	0.94	2.16	0.25
Amino Acid	Histidine Metabolism	1-methylhistamine	HMDB00898	0.94	0.89	1.13	0.77
Amino Acid	Histidine Metabolism	1-methyl-4-imidazoleacetate	HMDB02820	0.69	0.23	0.79	0.47
Amino Acid	Histidine Metabolism	4-imidazoleacetate	HMDB02024	1.04	0.92	1.4	0.40
Amino Acid	Histidine Metabolism	N-acetylhistamine	HMDB13253	0.86	0.73	1.61	0.30
Amino Acid	Lysine Metabolism	lysine	HMDB00182	0.8	0.21	1.29	0.17
Amino Acid	Lysine Metabolism	N2-acetyllysine	HMDB00446	0.92	0.79	1.08	0.81
Amino Acid	Lysine Metabolism	N6-acetyllysine	HMDB00206	0.73	0.10	0.93	0.71
Amino Acid	Lysine Metabolism	N2,N6-diacetyllysine		1.09	0.68	0.8	0.33
Amino Acid	Lysine Metabolism	N6-formyllysine		0.6	0.19	**2.37**	0.04
Amino Acid	Lysine Metabolism	N6-carboxyethyllysine		0.6	0.18	1.71	0.17
Amino Acid	Lysine Metabolism	N6,N6,N6-trimethyllysine	HMDB01325	**0.56**	0.08	1.03	0.94
Amino Acid	Lysine Metabolism	5-hydroxylysine	HMDB00450	0.91	0.78	1.28	0.52
Amino Acid	Lysine Metabolism	saccharopine	HMDB00279	1.05	0.92	1.36	0.50
Amino Acid	Lysine Metabolism	2-aminoadipate	HMDB00510	0.73	0.29	0.84	0.56
Amino Acid	Lysine Metabolism	pipecolate	HMDB00070	0.92	0.81	1.1	0.79
Amino Acid	Lysine Metabolism	6-oxopiperidine-2-carboxylate	HMDB61705	0.82	0.32	1.09	0.67
Amino Acid	Lysine Metabolism	cadaverine	HMDB02322	0.86	0.70	0.75	0.48

Amino Acid	Lysine Metabolism	N-acetyl-cadaverine	HMDB02284	0.76	0.50	0.78	0.55
Amino Acid	Lysine Metabolism	5-aminovalerate	HMDB03355	0.74	0.35	0.69	0.28
Amino Acid	Lysine Metabolism	N-trimethyl 5-aminovalerate		0.88	0.78	1.02	0.96
Amino Acid	Phenylalanine Metabolism	phenylalanine	HMDB00159	0.88	0.27	1.13	0.28
Amino Acid	Phenylalanine Metabolism	N-acetylphenylalanine	HMDB00512	1.06	0.85	1.11	0.76
Amino Acid	Phenylalanine Metabolism	phenylpyruvate	HMDB00205	0.59	0.03	1.18	0.53
Amino Acid	Phenylalanine Metabolism	phenyllactate (PLA)	HMDB00779	0.44	0.02	1.49	0.29
Amino Acid	Phenylalanine Metabolism	phenethylamine	HMDB02017	1.04	0.94	0.66	0.42
Amino Acid	Phenylalanine Metabolism	phenylacetate	HMDB00209	1.47	0.48	1.11	0.85
Amino Acid	Phenylalanine Metabolism	4-hydroxyphenylacetate	HMDB00020	0.95	0.93	1.65	0.37
Amino Acid	Phenylalanine Metabolism	3-hydroxyphenylacetate	HMDB00440	0.87	0.65	1	0.99
Amino Acid	Phenylalanine Metabolism	valerylphenylalanine		1.15	0.84	1.52	0.58
Amino Acid	Tyrosine Metabolism	tyrosine	HMDB00158	0.94	0.75	1.36	0.15
Amino Acid	Tyrosine Metabolism	N-acetyltyrosine	HMDB00866	0.88	0.62	1.55	0.12
Amino Acid	Tyrosine Metabolism	tyramine	HMDB00306	0.6	0.36	1.22	0.73
Amino Acid	Tyrosine Metabolism	4-hydroxyphenylpyruvate	HMDB00707	0.55	0.02	0.92	0.75
Amino Acid	Tyrosine Metabolism	3-(4-hydroxyphenyl)lactate	HMDB00755	0.55	0.17	2.06	0.11
Amino Acid	Tyrosine Metabolism	phenol sulfate	HMDB60015	0.66	0.47	1.38	0.59
Amino Acid	Tyrosine Metabolism	dopamine	HMDB00073	0.63	0.34	1.21	0.70
Amino Acid	Tyrosine Metabolism	vanillactate	HMDB00913	0.67	0.27	1.31	0.48
Amino Acid	Tyrosine Metabolism	vanillylmandelate (VMA)	HMDB00291	0.71	0.38	1.38	0.43
Amino Acid	Tyrosine Metabolism	3-methoxytyrosine	HMDB01434	0.85	0.60	1.08	0.80
Amino Acid	Tyrosine Metabolism	3-methoxytyramine sulfate		1.04	0.79	1.14	0.42
Amino Acid	Tyrosine Metabolism	(R)-salsolinol	HMDB05199	0.75	0.52	1.39	0.49
Amino Acid	Tyrosine Metabolism	o-Tyrosine	HMDB06050	0.71	0.21	1.32	0.33
Amino Acid	Tyrosine Metabolism	gentisate	HMDB00152	0.91	0.83	1.81	0.23
Amino Acid	Tyrosine Metabolism	2-hydroxyphenylacetate	HMDB00669	1.12	0.80	0.64	0.33
Amino Acid	Tyrosine Metabolism	dopamine 3-O-sulfate	HMDB06275	0.92	0.87	1.59	0.37
Amino Acid	Tyrosine Metabolism	tyramine O-sulfate	HMDB06409	1.08	0.90	0.4	0.15
Amino Acid	Tyrosine Metabolism	N-formylphenylalanine		0.83	0.41	1.47	0.12
Amino Acid	Tyrosine Metabolism	vanillic alcohol sulfate		0.7	0.26	2.01	0.04
Amino Acid	Tryptophan Metabolism	tryptophan	HMDB00929	0.78	0.14	1	1.00
Amino Acid	Tryptophan Metabolism	N-acetyltryptophan	HMDB13713	1.02	0.94	1.01	0.96
Amino Acid	Tryptophan Metabolism	C-glycosyltryptophan		0.78	0.58	0.95	0.90

Amino Acid	Tryptophan Metabolism	tryptophan betaine	HMDB61115	1.24	0.57	1.39	0.39
Amino Acid	Tryptophan Metabolism	kynurenine	HMDB00684	0.84	0.39	0.91	0.68
Amino Acid	Tryptophan Metabolism	N-acetylkynurenine (2)		1.05	0.80	0.98	0.91
Amino Acid	Tryptophan Metabolism	kynurenate	HMDB00715	**0.48**	0.01	1.13	0.66
Amino Acid	Tryptophan Metabolism	N-formylanthranilic acid	HMDB04089	1.2	0.54	**0.51**	0.03
Amino Acid	Tryptophan Metabolism	xanthurenate	HMDB00881	**0.47**	0.07	1.6	0.28
Amino Acid	Tryptophan Metabolism	picolinate	HMDB02243	0.96	0.86	0.93	0.78
Amino Acid	Tryptophan Metabolism	serotonin	HMDB00259	0.78	0.29	1.05	0.84
Amino Acid	Tryptophan Metabolism	tryptamine	HMDB00303	1.46	0.57	2.4	0.21
Amino Acid	Tryptophan Metabolism	indolepyruvate	HMDB60484	0.97	0.66	**1.13**	0.09
Amino Acid	Tryptophan Metabolism	indolelactate	HMDB00671	0.51	0.21	1.5	0.46
Amino Acid	Tryptophan Metabolism	indoleacetate	HMDB00197	0.7	0.31	1.28	0.50
Amino Acid	Tryptophan Metabolism	indolepropionate	HMDB02302	**4.67**	0.02	1.33	0.67
Amino Acid	Tryptophan Metabolism	indolepropionylglycine		0.98	0.93	1.13	0.62
Amino Acid	Tryptophan Metabolism	indoleacetylglutamine	HMDB13240	1.03	0.96	0.78	0.61
Amino Acid	Tryptophan Metabolism	skatol	HMDB00466	0.84	0.28	1.03	0.86
Amino Acid	Tryptophan Metabolism	indole	HMDB00738	1.17	0.69	1.08	0.86
Amino Acid	Tryptophan Metabolism	indole-3-carboxylic acid	HMDB03320	0.71	0.39	**0.5**	0.09
Amino Acid	Tryptophan Metabolism	3-indoxyl sulfate	HMDB00682	0.8	0.74	0.57	0.45
Amino Acid	Tryptophan Metabolism	5-bromotryptophan		1	1.00	0.94	0.50
Amino Acid	Leucine, Isoleucine and Valine Metabolism	leucine	HMDB00687	0.9	0.36	1.11	0.37
Amino Acid	Leucine, Isoleucine and Valine Metabolism	N-acetylleucine	HMDB11756	0.86	0.65	1.05	0.89
Amino Acid	Leucine, Isoleucine and Valine Metabolism	4-methyl-2-oxopentanoate	HMDB00695	**0.63**	0.09	1.22	0.47
Amino Acid	Leucine, Isoleucine and Valine Metabolism	alpha-hydroxyisocaproate	HMDB00746	**0.45**	0.03	1.05	0.89
Amino Acid	Leucine, Isoleucine and Valine Metabolism	isovalerate (i5:0)	HMDB00718	1.33	0.60	1.45	0.51
Amino Acid	Leucine, Isoleucine and Valine Metabolism	isovalerylglycine	HMDB00678	0.85	0.63	0.99	0.97
Amino Acid	Leucine, Isoleucine and Valine Metabolism	isovalerylcarnitine (C5)	HMDB00688	1.05	0.84	1.14	0.61
Amino Acid	Leucine, Isoleucine and Valine Metabolism	isovalerylglutamine		0.62	0.15	1.09	0.81
Amino Acid	Leucine, Isoleucine and Valine Metabolism	isovalerylhistidine		1.01	0.98	1.16	0.76

Amino Acid	Leucine, Isoleucine and Valine Metabolism	isovaleryltryptophan		0.85	0.72	1.45	0.44
Amino Acid	Leucine, Isoleucine and Valine Metabolism	beta-hydroxyisovalerate	HMDB00754	0.91	0.80	1.01	0.98
Amino Acid	Leucine, Isoleucine and Valine Metabolism	3-methylglutaconate	HMDB00522	0.85	0.46	0.87	0.55
Amino Acid	Leucine, Isoleucine and Valine Metabolism	5-methylnorleucine		0.86	0.36	1.24	0.21
Amino Acid	Leucine, Isoleucine and Valine Metabolism	isoleucine	HMDB00172	0.88	0.29	1.05	0.72
Amino Acid	Leucine, Isoleucine and Valine Metabolism	allo-isoleucine		0.67	0.27	1.43	0.34
Amino Acid	Leucine, Isoleucine and Valine Metabolism	N-acetylisoleucine	HMDB61684	0.78	0.44	1.07	0.83
Amino Acid	Leucine, Isoleucine and Valine Metabolism	3-methyl-2-oxovalerate	HMDB03736	0.64	0.12	1.29	0.39
Amino Acid	Leucine, Isoleucine and Valine Metabolism	alpha-hydroxyisovalerate	HMDB00407	**0.47**	0.03	1.08	0.83
Amino Acid	Leucine, Isoleucine and Valine Metabolism	2-methylbutyrylcarnitine (C5)	HMDB00378	0.8	0.55	1.4	0.39
Amino Acid	Leucine, Isoleucine and Valine Metabolism	2-methylbutyrylglycine	HMDB00339	0.81	0.41	1.1	0.72
Amino Acid	Leucine, Isoleucine and Valine Metabolism	ethylmalonate	HMDB00622	0.77	0.37	0.74	0.34
Amino Acid	Leucine, Isoleucine and Valine Metabolism	methylsuccinate	HMDB01844	0.72	0.34	1.08	0.83
Amino Acid	Leucine, Isoleucine and Valine Metabolism	2,3-dimethylsuccinate		1.08	0.86	1.17	0.70
Amino Acid	Leucine, Isoleucine and Valine Metabolism	valine	HMDB00883	**0.74**	0.10	1.17	0.39
Amino Acid	Leucine, Isoleucine and Valine Metabolism	N-acetylvaline	HMDB11757	0.68	0.20	1.19	0.58
Amino Acid	Leucine, Isoleucine and Valine Metabolism	3-methyl-2-oxobutyrate	HMDB00019	**0.52**	0.03	1.4	0.28
Amino Acid	Leucine, Isoleucine and Valine Metabolism	2-hydroxy-3-methylvalerate	HMDB00317	**0.46**	0.02	1.14	0.71
Amino Acid	Leucine, Isoleucine and Valine Metabolism	isobutyrylcarnitine (C4)	HMDB00736	0.91	0.81	**2.08**	0.07
Amino Acid	Leucine, Isoleucine and Valine Metabolism	isobutyrylglycine	HMDB00730	0.67	0.24	0.62	0.19
Amino Acid	Leucine, Isoleucine and Valine Metabolism	3-hydroxyisobutyrate	HMDB00336	1.96	0.13	1.87	0.18
Amino Acid	Leucine, Isoleucine and Valine Metabolism	2,3-dihydroxy-2-methylbutyrate	HMDB29576	2.71	0.21	1.25	0.79
Amino Acid	Methionine, Cysteine, SAM and	methionine	HMDB00696	0.79	0.47	**1.96**	0.05

	Taurine Metabolism						
Amino Acid	Methionine, Cysteine, SAM and Taurine Metabolism	N-acetylmethionine	HMDB11745	0.89	0.82	**3.24**	0.02
Amino Acid	Methionine, Cysteine, SAM and Taurine Metabolism	N-formylmethionine	HMDB01015	0.56	0.20	**2.84**	0.03
Amino Acid	Methionine, Cysteine, SAM and Taurine Metabolism	methionine sulfone		0.64	0.12	1.09	0.78
Amino Acid	Methionine, Cysteine, SAM and Taurine Metabolism	methionine sulfoxide	HMDB02005	1.04	0.82	0.82	0.28
Amino Acid	Methionine, Cysteine, SAM and Taurine Metabolism	N-acetylmethionine sulfoxide		1.07	0.78	0.86	0.55
Amino Acid	Methionine, Cysteine, SAM and Taurine Metabolism	4-methylthio-2-oxobutanoate	HMDB01553	0.61	0.11	1.23	0.52
Amino Acid	Methionine, Cysteine, SAM and Taurine Metabolism	alpha-ketobutyrate	HMDB00005	**0.53**	0.07	0.9	0.78
Amino Acid	Methionine, Cysteine, SAM and Taurine Metabolism	cysteine	HMDB00574	**0.66**	0.05	1.29	0.24
Amino Acid	Methionine, Cysteine, SAM and Taurine Metabolism	N-acetylcysteine	HMDB01890	0.76	0.28	1.21	0.47
Amino Acid	Methionine, Cysteine, SAM and Taurine Metabolism	S-methylcysteine	HMDB02108	**0.49**	0.05	1.39	0.38
Amino Acid	Methionine, Cysteine, SAM and Taurine Metabolism	cysteine s-sulfate	HMDB00731	**0.61**	0.08	1.18	0.57
Amino Acid	Methionine, Cysteine, SAM and Taurine Metabolism	cystine	HMDB00192	1.06	0.84	1.33	0.37
Amino Acid	Methionine, Cysteine, SAM and Taurine Metabolism	cysteine sulfinic acid	HMDB00996	0.68	0.15	1.28	0.37
Amino Acid	Methionine, Cysteine, SAM and Taurine Metabolism	hypotaurine	HMDB00965	**0.52**	0.04	0.9	0.75
Amino Acid	Methionine, Cysteine, SAM and Taurine Metabolism	taurine	HMDB00251	0.57	0.18	1.16	0.74
Amino Acid	Methionine, Cysteine, SAM and Taurine Metabolism	N-acetyltaurine		0.62	0.23	0.92	0.83
Amino Acid	Methionine, Cysteine, SAM and	3-sulfo-L-alanine	HMDB02757	1.06	0.81	1.05	0.83

	Taurine Metabolism						
Amino Acid	Urea cycle; Arginine and Proline Metabolism	arginine	HMDB00517	0.92	0.79	1.22	0.58
Amino Acid	Urea cycle; Arginine and Proline Metabolism	argininosuccinate	HMDB00052	1.19	0.52	0.92	0.76
Amino Acid	Urea cycle; Arginine and Proline Metabolism	ornithine	HMDB03374	0.82	0.48	1.13	0.68
Amino Acid	Urea cycle; Arginine and Proline Metabolism	norvaline	HMDB13716	0.84	0.78	0.65	0.53
Amino Acid	Urea cycle; Arginine and Proline Metabolism	2-oxoarginine*	HMDB04225	0.65	0.35	0.95	0.91
Amino Acid	Urea cycle; Arginine and Proline Metabolism	citrulline	HMDB00904	0.88	0.65	0.88	0.67
Amino Acid	Urea cycle; Arginine and Proline Metabolism	homoarginine	HMDB00670	0.74	0.47	1.53	0.32
Amino Acid	Urea cycle; Arginine and Proline Metabolism	homocitrulline	HMDB00679	0.84	0.64	0.97	0.94
Amino Acid	Urea cycle; Arginine and Proline Metabolism	proline	HMDB00162	**0.72**	0.08	1.23	0.30
Amino Acid	Urea cycle; Arginine and Proline Metabolism	dimethylarginine (SDMA + ADMA)	HMDB01539	**0.53**	0.01	0.92	0.74
Amino Acid	Urea cycle; Arginine and Proline Metabolism	N-acetylarginine	HMDB04620	1.19	0.62	0.65	0.25
Amino Acid	Urea cycle; Arginine and Proline Metabolism	N-acetylcitrulline	HMDB00856	0.94	0.84	**0.57**	0.09
Amino Acid	Urea cycle; Arginine and Proline Metabolism	N-acetylproline		0.65	0.15	1.09	0.78
Amino Acid	Urea cycle; Arginine and Proline Metabolism	N-delta-acetylornithine		0.81	0.38	0.9	0.69
Amino Acid	Urea cycle; Arginine and Proline Metabolism	N-alpha-acetylornithine	HMDB03357	0.81	0.48	1.15	0.65
Amino Acid	Urea cycle; Arginine and Proline Metabolism	N2,N5-diacetylornithine		0.93	0.81	0.9	0.75
Amino Acid	Urea cycle; Arginine and Proline Metabolism	trans-4-hydroxyproline	HMDB00725	0.73	0.37	1.13	0.74
Amino Acid	Urea cycle; Arginine and Proline Metabolism	pro-hydroxy-pro	HMDB06695	0.6	0.14	0.84	0.64
Amino Acid	Urea cycle; Arginine and Proline Metabolism	N-methylproline		1.08	0.89	1.57	0.42
Amino Acid	Urea cycle; Arginine and Proline Metabolism	1-propanoylproline		1		1	
Amino Acid	Urea cycle; Arginine and Proline Metabolism	N-monomethylarginine	HMDB29416	0.61	0.1188	1.14	0.6917
Amino Acid	Urea cycle; Arginine and Proline Metabolism	argininate*	HMDB03148	0.77	0.4317	0.91	0.7845

Amino Acid	Creatine Metabolism	guanidinoacetate	HMDB00128	0.79	0.5288	0.54	0.1254
Amino Acid	Creatine Metabolism	creatine	HMDB00064	0.74	0.6977	1.72	0.4937
Amino Acid	Creatine Metabolism	creatinine	HMDB00562	0.31	0.1835	1.12	0.9044
Amino Acid	Creatine Metabolism	N-methylhydantoin	HMDB03646	0.92	0.8558	0.71	0.4788
Amino Acid	Creatine Metabolism	N-carbamoylsarcosine	HMDB12265	1.3	0.5825	**0.38**	0.0515
Amino Acid	Polyamine Metabolism	agmatine	HMDB01432	0.45	0.2150	0.68	0.5571
Amino Acid	Polyamine Metabolism	putrescine	HMDB01414	0.75	0.4331	0.86	0.6775
Amino Acid	Polyamine Metabolism	spermidine	HMDB01257	0.9	0.8593	0.67	0.4912
Amino Acid	Polyamine Metabolism	acisoga		0.62	0.1477	0.58	0.1154
Amino Acid	Polyamine Metabolism	spermine	HMDB01256	1		1	
Amino Acid	Polyamine Metabolism	N(1)-acetylspermine	HMDB01186	0.73	0.5383	0.95	0.9281
Amino Acid	Polyamine Metabolism	N1,N12-diacetylspermine	HMDB02172	0.76	0.5298	1.1	0.8310
Amino Acid	Polyamine Metabolism	5-methylthioadenosine (MTA)	HMDB01173	1.01	0.9678	0.85	0.6179
Amino Acid	Polyamine Metabolism	N-acetylputrescine	HMDB02064	**0.51**	0.0718	0.82	0.6059
Amino Acid	Polyamine Metabolism	4-acetamidobutanoate	HMDB03681	0.75	0.2275	1.03	0.8946
Amino Acid	Polyamine Metabolism	(N(1) + N(8))-acetylspermidine		0.62	0.1683	1.05	0.8857
Amino Acid	Guanidino and Acetamido Metabolism	1-methylguanidine	HMDB01522	0.71	0.1919	1.48	0.1603
Amino Acid	Guanidino and Acetamido Metabolism	4-guanidinobutanoate	HMDB03464	0.83	0.4702	1.43	0.1771
Amino Acid	Guanidino and Acetamido Metabolism	guanidinosuccinate	HMDB03157	0.88	0.4943	1.06	0.7449
Amino Acid	Glutathione Metabolism	cysteine-glutathione disulfide	HMDB00656	1.04	0.7529	1.01	0.9102
Amino Acid	Glutathione Metabolism	5-oxoproline	HMDB00267	**0.5**	0.0085	1.27	0.3819
Amino Acid	Glutathione Metabolism	2-aminobutyrate	HMDB00650	0.54	0.1996	0.46	0.1331
Amino Acid	Glutathione Metabolism	2-hydroxybutyrate/2-hydroxyisobutyrate		**0.45**	0.0153	0.73	0.3554
Amino Acid	Glutathione Metabolism	4-amino-2-hydroxybutyrate		0.91	0.8055	0.86	0.7278
Peptide	Gamma-glutamyl Amino Acid	gamma-glutamylalanine	HMDB29142	0.7	0.3539	1.39	0.4058
Peptide	Gamma-glutamyl Amino Acid	gamma-glutamylglutamate	HMDB11737	1.63	0.3485	1.05	0.9258
Peptide	Gamma-glutamyl Amino Acid	gamma-glutamylglutamine	HMDB11738	**0.49**	0.0278	1.05	0.8844
Peptide	Gamma-glutamyl Amino Acid	gamma-glutamylglycine	HMDB11667	0.55	0.1578	**2.21**	0.0724
Peptide	Gamma-glutamyl Amino Acid	gamma-glutamylhistidine		0.76	0.5057	0.96	0.9228
Peptide	Gamma-glutamyl Amino Acid	gamma-glutamylisoleucine*	HMDB11170	0.91	0.7904	1.54	0.2570
Peptide	Gamma-glutamyl Amino Acid	gamma-glutamylleucine	HMDB11171	0.86	0.6597	1.57	0.1996
Peptide	Gamma-glutamyl Amino Acid	gamma-glutamyl-alpha-lysine		0.98	0.9304	1.38	0.2266

Peptide	Gamma-glutamyl Amino Acid	gamma-glutamyl-epsilon-lysine	HMDB03869	**0.49**	0.0137	0.84	0.5553
Peptide	Gamma-glutamyl Amino Acid	gamma-glutamylmethionine	HMDB29155	1.04	0.9377	2.11	0.1285
Peptide	Gamma-glutamyl Amino Acid	gamma-glutamylphenylalanine	HMDB00594	0.7	0.3271	1.6	0.2115
Peptide	Gamma-glutamyl Amino Acid	gamma-glutamylthreonine	HMDB29159	0.95	0.8991	1.1	0.8194
Peptide	Gamma-glutamyl Amino Acid	gamma-glutamyltryptophan	HMDB29160	0.82	0.6082	1.4	0.3963
Peptide	Gamma-glutamyl Amino Acid	gamma-glutamyltyrosine	HMDB11741	0.7	0.3668	1.59	0.2464
Peptide	Gamma-glutamyl Amino Acid	gamma-glutamylvaline	HMDB11172	0.67	0.4551	2.08	0.1882
Peptide	Gamma-glutamyl Amino Acid	gamma-glutamyl-2-aminobutyrate		1.28	0.5706	1	0.9981
Peptide	Dipeptide	alanylleucine	HMDB28691	1.03	0.9480	0.9	0.7961
Peptide	Dipeptide	glycylisoleucine	HMDB28844	0.97	0.9235	0.74	0.3017
Peptide	Dipeptide	glycylleucine	HMDB00759	0.97	0.9184	0.77	0.3515
Peptide	Dipeptide	glycylvaline	HMDB28854	0.89	0.6428	0.8	0.3933
Peptide	Dipeptide	isoleucylglycine	HMDB28907	0.93	0.8767	0.87	0.7558
Peptide	Dipeptide	leucylalanine	HMDB28922	1.85	0.2104	1.1	0.8464
Peptide	Dipeptide	leucylglycine	HMDB28929	1.23	0.6105	0.81	0.6274
Peptide	Dipeptide	lysylleucine	HMDB28955	0.71	0.4994	1.42	0.5058
Peptide	Dipeptide	phenylalanylalanine		1.22	0.5802	0.81	0.5787
Peptide	Dipeptide	phenylalanylglycine	HMDB28995	0.98	0.9603	0.85	0.7296
Peptide	Dipeptide	prolylglycine	HMDB11178	0.52	0.1240	1.12	0.7908
Peptide	Dipeptide	threonylphenylalanine	HMDB29068	1.04	0.9246	0.97	0.9504
Peptide	Dipeptide	tryptophylglycine	HMDB29083	1.03	0.9535	**0.42**	0.0957
Peptide	Dipeptide	tyrosylglycine	HMDB29105	0.93	0.9027	0.89	0.8454
Peptide	Dipeptide	valylglutamine	HMDB29125	0.88	0.7982	0.75	0.5783
Peptide	Dipeptide	valylglycine	HMDB29127	0.89	0.8232	0.96	0.9490
Peptide	Dipeptide	valylleucine	HMDB29131	1.36	0.5069	1.04	0.9342
Peptide	Dipeptide	leucylglutamine*	HMDB28927	1.22	0.6496	0.88	0.7917
Peptide	Polypeptide	alanyl-glutamyl-meso-diaminopimelate		0.95	0.9094	0.49	0.1544
Peptide	Acetylated Peptides	phenylacetylalanine		1.09	0.8203	0.89	0.7699
Peptide	Acetylated Peptides	phenylacetylhistidine		0.89	0.6945	0.87	0.6500
Peptide	Acetylated Peptides	phenylacetylglutamate	HMDB59772	1.25	0.6201	0.97	0.9415
Peptide	Acetylated Peptides	phenylacetylglutamine	HMDB06344	0.55	0.4249	0.34	0.1717
Peptide	Acetylated Peptides	phenylacetylglycine	HMDB00821	0.75	0.5903	1.11	0.8502
Peptide	Acetylated Peptides	4-hydroxyphenylacetylglycine		0.63	0.2054	1.59	0.2175
Peptide	Acetylated Peptides	phenylacetylmethionine		0.99	0.9875	1.42	0.3911

Peptide	Acetylated Peptides	phenylacetylphenylalanine		1.08	0.8611	1	0.9992
Carbohydrate	Glycolysis, Gluconeogenesis, and Pyruvate Metabolism	1,5-anhydroglucitol (1,5-AG)	HMDB02712	0.91	0.5487	0.83	0.2867
Carbohydrate	Glycolysis, Gluconeogenesis, and Pyruvate Metabolism	glucose	HMDB00122	0.92	0.8142	1.64	0.1717
Carbohydrate	Glycolysis, Gluconeogenesis, and Pyruvate Metabolism	pyruvate	HMDB00243	**0.46**	0.0237	1.26	0.5202
Carbohydrate	Glycolysis, Gluconeogenesis, and Pyruvate Metabolism	lactate	HMDB00190	0.48	0.1483	1.04	0.9411
Carbohydrate	Glycolysis, Gluconeogenesis, and Pyruvate Metabolism	glycerate	HMDB00139	0.84	0.5264	0.89	0.6648
Carbohydrate	Glycolysis, Gluconeogenesis, and Pyruvate Metabolism	glucosylglycerol		0.52	0.2177	**0.39**	0.0941
Carbohydrate	Pentose Metabolism	ribose	HMDB00283	1.12	0.7537	**1.9**	0.0810
Carbohydrate	Pentose Metabolism	ribitol	HMDB00508	0.9	0.8277	2.15	0.1384
Carbohydrate	Pentose Metabolism	ribonate	HMDB00867	0.63	0.4202	**0.36**	0.0882
Carbohydrate	Pentose Metabolism	xylose	HMDB00098	1.8	0.3714	2.26	0.2409
Carbohydrate	Pentose Metabolism	arabinose	HMDB00646	1.66	0.3040	0.82	0.6996
Carbohydrate	Pentose Metabolism	fucose	HMDB00174	0.71	0.3592	0.85	0.6630
Carbohydrate	Pentose Metabolism	arabitol/xylitol		1.05	0.9153	1.01	0.9767
Carbohydrate	Pentose Metabolism	arabonate/xylonate		1.03	0.9490	1.24	0.6334
Carbohydrate	Pentose Metabolism	sedoheptulose	HMDB03219	0.99	0.9805	0.48	0.2365
Carbohydrate	Glycogen Metabolism	maltotetraose	HMDB01296	0.75	0.5822	1.62	0.3657
Carbohydrate	Glycogen Metabolism	maltotriose	HMDB01262	1.17	0.7439	0.7	0.4738
Carbohydrate	Glycogen Metabolism	maltose	HMDB00163	1.11	0.8300	0.56	0.2670
Carbohydrate	Disaccharides and Oligosaccharides	lactose	HMDB00186	1.09	0.8770	1.99	0.2124
Carbohydrate	Disaccharides and Oligosaccharides	lacto-N-fucopentaose I		0.97	0.8666	0.99	0.9603
Carbohydrate	Disaccharides and Oligosaccharides	lacto-N-fucopentaose II		0.99	0.9528	1.05	0.8591
Carbohydrate	Disaccharides and Oligosaccharides	lacto-N-tetraose	HMDB06566	1.09	0.7599	1.06	0.8255
Carbohydrate	Disaccharides and Oligosaccharides	lacto-N-neotetraose		0.63	0.2855	1.36	0.4907
Carbohydrate	Disaccharides and Oligosaccharides	3-sialyllactose	HMDB00825	**0.8**	0.0484	1	1.0000
Carbohydrate	Disaccharides and Oligosaccharides	6'-sialyllactose	HMDB06569	0.94	0.7974	0.99	0.9809
Carbohydrate	Disaccharides and Oligosaccharides	2-fucosyllactose	HMDB02098	0.8	0.6640	2.17	0.1331
Carbohydrate	Disaccharides and Oligosaccharides	3-fucosyllactose	HMDB02094	1.03	0.9483	1.68	0.3171

Carbohydrate	Disaccharides and Oligosaccharides	Lewis a trisaccharide		1.36	0.0443	0.99	0.9520
Carbohydrate	Disaccharides and Oligosaccharides	Lewis X trisaccharide	HMDB06568	1	0.9313	1	1.0000
Carbohydrate	Disaccharides and Oligosaccharides	sucrose	HMDB00258	1.52	0.2481	0.67	0.2822
Carbohydrate	Fructose, Mannose and Galactose Metabolism	fructose	HMDB00660	1.06	0.9115	1.02	0.9748
Carbohydrate	Fructose, Mannose and Galactose Metabolism	mannitol/sorbitol	HMDB00247	0.94	0.8918	1.57	0.3818
Carbohydrate	Fructose, Mannose and Galactose Metabolism	mannose	HMDB00169	0.83	0.6772	2.04	0.1259
Carbohydrate	Fructose, Mannose and Galactose Metabolism	galactose	HMDB00143	0.8	0.4492	0.84	0.5545
Carbohydrate	Fructose, Mannose and Galactose Metabolism	galactitol (dulcitol)	HMDB00107	0.59	0.4508	1.12	0.8747
Carbohydrate	Fructose, Mannose and Galactose Metabolism	galactonate	HMDB00565	0.85	0.7715	1.15	0.8173
Carbohydrate	Aminosugar Metabolism	glucosamine	HMDB01514	0.68	0.2076	1.21	0.5521
Carbohydrate	Aminosugar Metabolism	glucosamine 6-sulfate	HMDB00592	0.61	0.5461	2.1	0.3786
Carbohydrate	Aminosugar Metabolism	glucuronate	HMDB00127	0.84	0.7156	0.82	0.6856
Carbohydrate	Aminosugar Metabolism	N-acetylglucosamine 6-sulfate	HMDB00841	0.66	0.4806	2.53	0.1313
Carbohydrate	Aminosugar Metabolism	N-acetylneuraminate	HMDB00230	0.69	0.3908	1.07	0.8769
Carbohydrate	Aminosugar Metabolism	N-acetyl-beta-glucosaminylamine	HMDB01104	0.88	0.8047	0.83	0.7201
Carbohydrate	Aminosugar Metabolism	N-acetylmuramate	HMDB60493	1.17	0.6353	1.6	0.1651
Carbohydrate	Aminosugar Metabolism	6-sialyl-N-acetyllactosamine	HMDB06584	1.05	0.8548	1.57	0.1345
Carbohydrate	Aminosugar Metabolism	N-acetylglucosaminylasparagine	HMDB00489	0.65	0.2608	1.17	0.6864
Carbohydrate	Aminosugar Metabolism	erythronate*	HMDB00613	0.75	0.4044	1.3	0.4606
Carbohydrate	Aminosugar Metabolism	N-acetylglucosamine/N-acetylgalactosamine	HMDB00215	0.9	0.7447	1.15	0.6902
Carbohydrate	Advanced Glycation End-product	N6-carboxymethyllysine		0.68	0.1001	1.36	0.2126
Energy	TCA Cycle	citrate	HMDB00094	0.56	0.3396	0.46	0.2271
Energy	TCA Cycle	aconitate [cis or trans]		1.44	0.5431	1.03	0.9567
Energy	TCA Cycle	isocitric lactone		0.53	0.3503	1.55	0.5414
Energy	TCA Cycle	alpha-ketoglutarate	HMDB00208	0.65	0.1785	1.96	0.0484
Energy	TCA Cycle	succinylcarnitine (C4-DC)	HMDB61717	0.68	0.3075	0.99	0.9777
Energy	TCA Cycle	succinate	HMDB00254	1.18	0.6810	0.87	0.7303
Energy	TCA Cycle	fumarate	HMDB00134	1.04	0.9307	1.06	0.8993
Energy	TCA Cycle	malate	HMDB00156	1.19	0.5626	0.88	0.6846
Energy	TCA Cycle	tricarballylate	HMDB31193	0.76	0.4707	0.75	0.4546

Energy	TCA Cycle	citraconate/glutaconate		1.06	0.7325	1.12	0.5480
Energy	TCA Cycle	2-methylcitrate/homocitrate		1.12	0.7784	0.8	0.6043
Energy	Oxidative Phosphorylation	phosphate	HMDB01429	2.04	0.2073	1.2	0.7520
Lipid	Fatty Acid Synthesis	malonylcarnitine	HMDB02095	**0.62**	0.0871	1	0.9969
Lipid	Fatty Acid Synthesis	malonate	HMDB00691	1.19	0.5826	1.01	0.9820
Lipid	Short Chain Fatty Acid	valerate (5:0)	HMDB00892	1.19	0.7583	1.95	0.2713
Lipid	Medium Chain Fatty Acid	caproate (6:0)	HMDB00535	0.85	0.7455	1.21	0.7182
Lipid	Medium Chain Fatty Acid	heptanoate (7:0)	HMDB00666	0.64	0.2881	0.87	0.7561
Lipid	Medium Chain Fatty Acid	caprylate (8:0)	HMDB00482	1.06	0.7535	0.94	0.7415
Lipid	Medium Chain Fatty Acid	caprate (10:0)	HMDB00511	0.87	0.5483	1.03	0.9026
Lipid	Medium Chain Fatty Acid	undecanoate (11:0)	HMDB00947	0.95	0.7658	0.9	0.5437
Lipid	Medium Chain Fatty Acid	laurate (12:0)	HMDB00638	0.65	0.2049	0.78	0.4782
Lipid	Medium Chain Fatty Acid	5-dodecenoate (12:1n7)	HMDB00529	1	0.9978	0.87	0.6846
Lipid	Long Chain Fatty Acid	myristate (14:0)	HMDB00806	0.75	0.3197	0.86	0.6189
Lipid	Long Chain Fatty Acid	myristoleate (14:1n5)	HMDB02000	1.03	0.8923	1.1	0.7136
Lipid	Long Chain Fatty Acid	pentadecanoate (15:0)	HMDB00826	1.15	0.6403	1.06	0.8358
Lipid	Long Chain Fatty Acid	palmitate (16:0)	HMDB00220	0.88	0.5893	1.17	0.5041
Lipid	Long Chain Fatty Acid	palmitoleate (16:1n7)	HMDB03229	0.94	0.8218	0.97	0.9154
Lipid	Long Chain Fatty Acid	margarate (17:0)	HMDB02259	0.87	0.6316	1.19	0.5690
Lipid	Long Chain Fatty Acid	10-heptadecenoate (17:1n7)	HMDB60038	0.89	0.7284	0.87	0.6666
Lipid	Long Chain Fatty Acid	trans-heptadecenoate (tr 17:1)*		0.98	0.9629	1.17	0.6612
Lipid	Long Chain Fatty Acid	stearate (18:0)	HMDB00827	0.94	0.8217	1.13	0.6366
Lipid	Long Chain Fatty Acid	oleate/vaccenate (18:1)		0.89	0.6700	1.34	0.3059
Lipid	Long Chain Fatty Acid	nonadecanoate (19:0)	HMDB00772	0.9	0.7441	1.55	0.1783
Lipid	Long Chain Fatty Acid	10-nonadecenoate (19:1n9)	HMDB13622	0.65	0.1860	0.94	0.8616
Lipid	Long Chain Fatty Acid	trans-nonadecenoate (tr 19:1)*		0.97	0.9194	1.06	0.8608
Lipid	Long Chain Fatty Acid	arachidate (20:0)	HMDB02212	0.98	0.9479	**1.72**	0.0772
Lipid	Long Chain Fatty Acid	eicosenoate (20:1)	HMDB02231	0.73	0.2877	1.38	0.2895
Lipid	Long Chain Fatty Acid	erucate (22:1n9)	HMDB02068	0.8	0.5187	1.26	0.5072
Lipid	Polyunsaturated Fatty Acid (n3 and n6)	heneicosapentaenoate (21:5n3)		0.96	0.8612	0.84	0.4439
Lipid	Polyunsaturated Fatty Acid (n3 and n6)	hexadecadienoate (16:2n6)	HMDB00477	0.85	0.5093	1.01	0.9713
Lipid	Polyunsaturated Fatty Acid (n3 and n6)	hexadecatrienoate (16:3n3)		0.98	0.9430	0.81	0.5486

Lipid	Polyunsaturated Fatty Acid (n3 and n6)	stearidonate (18:4n3)	HMDB06547	**0.43**	0.0511	1.37	0.4840
Lipid	Polyunsaturated Fatty Acid (n3 and n6)	eicosapentaenoate (EPA; 20:5n3)	HMDB01999	0.62	0.1691	0.88	0.7232
Lipid	Polyunsaturated Fatty Acid (n3 and n6)	docosapentaenoate (n3 DPA; 22:5n3)	HMDB06528	0.91	0.7939	0.95	0.9003
Lipid	Polyunsaturated Fatty Acid (n3 and n6)	docosahexaenoate (DHA; 22:6n3)	HMDB02183	**0.49**	0.0635	0.96	0.9172
Lipid	Polyunsaturated Fatty Acid (n3 and n6)	docosatrienoate (22:3n3)	HMDB02823	0.77	0.5258	0.94	0.8822
Lipid	Polyunsaturated Fatty Acid (n3 and n6)	omega-3 arachidonate (20:4n3)	HMDB02177	1.04	0.7929	1	1.0000
Lipid	Polyunsaturated Fatty Acid (n3 and n6)	nisinate (24:6n3)	HMDB02007	0.83	0.6598	0.78	0.5691
Lipid	Polyunsaturated Fatty Acid (n3 and n6)	linoleate (18:2n6)	HMDB00673	1.12	0.7118	**1.69**	0.0921
Lipid	Polyunsaturated Fatty Acid (n3 and n6)	linolenate [alpha or gamma; (18:3n3 or 6)]	HMDB03073	1.28	0.4446	1.69	0.1224
Lipid	Polyunsaturated Fatty Acid (n3 and n6)	dihomo-linolenate (20:3n3 or n6)	HMDB02925	1.01	0.9706	1.01	0.9831
Lipid	Polyunsaturated Fatty Acid (n3 and n6)	arachidonate (20:4n6)	HMDB01043	0.7	0.2967	0.92	0.8166
Lipid	Polyunsaturated Fatty Acid (n3 and n6)	adrenate (22:4n6)	HMDB02226	1	0.9901	0.78	0.5377
Lipid	Polyunsaturated Fatty Acid (n3 and n6)	docosapentaenoate (n6 DPA; 22:5n6)	HMDB01976	0.81	0.6475	0.91	0.8450
Lipid	Polyunsaturated Fatty Acid (n3 and n6)	docosadienoate (22:2n6)	HMDB61714	0.74	0.4620	0.87	0.7358
Lipid	Polyunsaturated Fatty Acid (n3 and n6)	dihomo-linoleate (20:2n6)	HMDB05060	0.69	0.3007	0.93	0.8487
Lipid	Polyunsaturated Fatty Acid (n3 and n6)	mead acid (20:3n9)	HMDB10378	0.45	0.2616	1	0.9950
Lipid	Polyunsaturated Fatty Acid (n3 and n6)	docosatrienoate (22:3n6)*		0.88	0.7963	0.98	0.9635
Lipid	Fatty Acid Hydroxyl Fatty Acid	PAHSA (16:0/OH-18:0)		0.61	0.1742	0.96	0.9202
Lipid	Fatty Acid Hydroxyl Fatty Acid	LAHSA (18:2/OH-18:0)*		0.94	0.8699	1.17	0.7061
Lipid	Fatty Acid Hydroxyl Fatty Acid	OAHSA (18:1/OH-18:0)		0.81	0.5961	1.1	0.8296
Lipid	Fatty Acid, Branched	isocaproate (i6:0)	HMDB00689	0.94	0.9162	1.71	0.3463
Lipid	Fatty Acid, Branched	13-methylmyristate (i15:0)		0.92	0.8472	0.94	0.8866
Lipid	Fatty Acid, Branched	15-methylpalmitate (i17:0)		0.92	0.7691	1.15	0.6476
Lipid	Fatty Acid, Branched	15-methylmargarate (a18:0)		1.31	0.4471	1.15	0.7031

Lipid	Fatty Acid, Branched	17-methylstearate (i19:0)	HMDB37397	0.88	0.6744	1.25	0.4533
Lipid	Fatty Acid, Branched	pristanate	HMDB00795	1.32	0.6127	1.47	0.4875
Lipid	Fatty Acid, Dicarboxylate	dimethylmalonic acid	HMDB02001	0.88	0.6576	1.45	0.2145
Lipid	Fatty Acid, Dicarboxylate	glutarate (C5-DC)	HMDB00661	1.02	0.9634	0.75	0.4547
Lipid	Fatty Acid, Dicarboxylate	3-methylglutarate/2-methylglutarate	HMDB00752	0.86	0.6382	0.93	0.8216
Lipid	Fatty Acid, Dicarboxylate	2-hydroxyglutarate	HMDB00606	0.67	0.2314	1.69	0.1328
Lipid	Fatty Acid, Dicarboxylate	adipate (C6-DC)	HMDB00448	0.7	0.1357	1.18	0.5019
Lipid	Fatty Acid, Dicarboxylate	2-hydroxyadipate	HMDB00321	0.87	0.6449	1.12	0.7298
Lipid	Fatty Acid, Dicarboxylate	3-methyladipate	HMDB00555	0.64	0.2408	0.63	0.2441
Lipid	Fatty Acid, Dicarboxylate	maleate	HMDB00176	0.84	0.3419	1.14	0.4991
Lipid	Fatty Acid, Dicarboxylate	pimelate (C7-DC)	HMDB00857	0.93	0.7811	**2.13**	0.0078
Lipid	Fatty Acid, Dicarboxylate	suberate (C8-DC)	HMDB00893	0.95	0.7889	1	0.9837
Lipid	Fatty Acid, Dicarboxylate	azelate (C9-DC)	HMDB00784	1	0.9925	1.18	0.3261
Lipid	Fatty Acid, Dicarboxylate	sebacate (C10-DC)	HMDB00792	1.01	0.9484	0.94	0.7680
Lipid	Fatty Acid, Dicarboxylate	undecanedioate (C11-DC)	HMDB00888	0.93	0.6061	0.99	0.9272
Lipid	Fatty Acid, Dicarboxylate	dodecanedioate (C12-DC)	HMDB00623	1.02	0.9012	1.08	0.6024
Lipid	Fatty Acid, Dicarboxylate	tetradecanedioate (C14-DC)	HMDB00872	1.24	0.1242	0.98	0.8885
Lipid	Fatty Acid, Dicarboxylate	hexadecanedioate (C16-DC)	HMDB00672	1.32	0.1153	0.91	0.6133
Lipid	Fatty Acid, Amide	oleamide	HMDB02117	1.28	0.5222	0.62	0.2324
Lipid	Fatty Acid, Amide	linoleamide (18:2n6)		1.57	0.3321	0.67	0.4009
Lipid	Fatty Acid, Amino	2-aminoheptanoate		**0.55**	0.0859	1.03	0.9379
Lipid	Fatty Acid Metabolism (also BCAA Metabolism)	butyrylglycine	HMDB00808	0.99	0.9822	2.3	0.1120
Lipid	Fatty Acid Metabolism (also BCAA Metabolism)	propionylcarnitine (C3)	HMDB00824	0.64	0.3322	1.4	0.4834
Lipid	Fatty Acid Metabolism (also BCAA Metabolism)	propionylglycine	HMDB00783	0.85	0.7333	1.12	0.8216
Lipid	Fatty Acid Metabolism (also BCAA Metabolism)	methylmalonate (MMA)	HMDB00202	0.92	0.8832	1.41	0.5425
Lipid	Fatty Acid Metabolism (Acyl Glutamine)	hexanoylglutamine		0.64	0.3880	0.44	0.1206
Lipid	Fatty Acid Metabolism(Acyl Glycine)	valerylglycine	HMDB00927	0.63	0.3034	1.73	0.2463
Lipid	Fatty Acid Metabolism(Acyl Glycine)	hexanoylglycine	HMDB00701	0.81	0.6930	1.33	0.6114
Lipid	Fatty Acid Metabolism(Acyl Glycine)	heptanoyl glycine		1.18	0.6975	1.27	0.5871
Lipid	Fatty Acid Metabolism(Acyl Glycine)	N-octanoylglycine	HMDB00832	1	1.0000	0.88	0.2990

Lipid	Fatty Acid Metabolism(Acyl Glycine)	N-palmitoylglycine	HMDB13034	1.03	0.9342	1.15	0.6711
Lipid	Fatty Acid Metabolism(Acyl Glycine)	N-oleoylglycine	HMDB13631	1.5	0.4704	1.16	0.7939
Lipid	Fatty Acid Metabolism(Acyl Glycine)	N-linoleoylglycine		0.85	0.6966	1.98	0.1290
Lipid	Fatty Acid Metabolism(Acyl Carnitine)	acetylcarnitine (C2)	HMDB00201	1.79	0.4574	0.79	0.7780
Lipid	Fatty Acid Metabolism(Acyl Carnitine)	3-hydroxybutyrylcarnitine (1)	HMDB13127	1.08	0.7806	1.25	0.4618
Lipid	Fatty Acid Metabolism(Acyl Carnitine)	3-hydroxybutyrylcarnitine (2)	HMDB13127	0.78	0.5784	1.95	0.1543
Lipid	Fatty Acid Metabolism(Acyl Carnitine)	hexanoylcarnitine (C6)	HMDB00705	0.76	0.3067	1.32	0.3367
Lipid	Fatty Acid Metabolism(Acyl Carnitine)	octanoylcarnitine (C8)	HMDB00791	0.82	0.6618	1.79	0.2048
Lipid	Fatty Acid Metabolism(Acyl Carnitine)	decanoylcarnitine (C10)	HMDB00651	0.85	0.7380	0.86	0.7769
Lipid	Fatty Acid Metabolism(Acyl Carnitine)	cis-4-decenoylcarnitine (C10:1)		1.18	0.7960	1.62	0.4526
Lipid	Fatty Acid Metabolism(Acyl Carnitine)	laurylcarnitine (C12)	HMDB02250	0.84	0.7964	0.9	0.8777
Lipid	Fatty Acid Metabolism(Acyl Carnitine)	myristoylcarnitine (C14)	HMDB05066	1.02	0.9666	0.61	0.4021
Lipid	Fatty Acid Metabolism(Acyl Carnitine)	palmitoylcarnitine (C16)	HMDB00222	0.84	0.7123	**0.45**	0.0985
Lipid	Fatty Acid Metabolism(Acyl Carnitine)	palmitoleoylcarnitine (C16:1)*		1	0.9981	1.14	0.7913
Lipid	Fatty Acid Metabolism(Acyl Carnitine)	stearoylcarnitine (C18)	HMDB00848	0.75	0.2644	0.7	0.1879
Lipid	Fatty Acid Metabolism(Acyl Carnitine)	linoleoylcarnitine (C18:2)*	HMDB06469	1.18	0.7219	0.6	0.2937
Lipid	Fatty Acid Metabolism(Acyl Carnitine)	linolenoylcarnitine (C18:3)*		0.99	0.9648	0.71	0.2768
Lipid	Fatty Acid Metabolism(Acyl Carnitine)	oleoylcarnitine (C18:1)	HMDB05065	0.88	0.7615	0.48	0.1032
Lipid	Fatty Acid Metabolism(Acyl Carnitine)	myristoleoylcarnitine (C14:1)*		0.9	0.8642	2.06	0.2538
Lipid	Fatty Acid Metabolism(Acyl Carnitine)	suberoylcarnitine (C8-DC)		0.88	0.7835	1.65	0.3025
Lipid	Fatty Acid Metabolism(Acyl Carnitine)	adipoylcarnitine (C6-DC)	HMDB61677	0.61	0.1821	1.5	0.2980
Lipid	Fatty Acid Metabolism(Acyl Carnitine)	arachidoylcarnitine (C20)*	HMDB06460	0.88	0.6527	0.93	0.8083
Lipid	Fatty Acid Metabolism(Acyl Carnitine)	arachidonoylcarnitine (C20:4)		0.69	0.1752	0.75	0.3075

Lipid	Fatty Acid Metabolism(Acyl Carnitine)	behenoylcarnitine (C22)*		0.81	0.4088	1.27	0.3748
Lipid	Fatty Acid Metabolism(Acyl Carnitine)	dihomo-linolenoylcarnitine (20:3n3 or 6)*		0.81	0.4679	0.72	0.2645
Lipid	Fatty Acid Metabolism(Acyl Carnitine)	dihomo-linoleoylcarnitine (C20:2)*		0.89	0.6805	0.79	0.4223
Lipid	Fatty Acid Metabolism(Acyl Carnitine)	eicosenoylcarnitine (C20:1)*		0.82	0.4790	0.85	0.5950
Lipid	Fatty Acid Metabolism(Acyl Carnitine)	erucoylcarnitine (C22:1)*		0.74	0.3224	0.88	0.6794
Lipid	Fatty Acid Metabolism(Acyl Carnitine)	docosahexaenoylcarnitine (C22:6)*		0.7	0.3946	0.89	0.7902
Lipid	Fatty Acid Metabolism(Acyl Carnitine)	lignoceroylcarnitine (C24)*		0.83	0.4163	1.19	0.4605
Lipid	Fatty Acid Metabolism(Acyl Carnitine)	margaroylcarnitine (C17)*	HMDB06210	0.82	0.5110	0.89	0.6912
Lipid	Fatty Acid Metabolism(Acyl Carnitine)	nervonoylcarnitine (C24:1)*		0.92	0.7505	0.93	0.8089
Lipid	Fatty Acid Metabolism(Acyl Carnitine)	cerotoylcarnitine (C26)*	HMDB06347	1.01	0.9696	0.76	0.2926
Lipid	Fatty Acid Metabolism(Acyl Carnitine)	ximenoylcarnitine (C26:1)*		0.93	0.7411	0.85	0.4903
Lipid	Fatty Acid Metabolism(Acyl Carnitine)	pentadecanoylcarnitine (C15)*		0.84	0.6347	0.92	0.8269
Lipid	Carnitine Metabolism	deoxycarnitine	HMDB01161	0.7	0.1769	0.67	0.1374
Lipid	Carnitine Metabolism	carnitine	HMDB00062	0.95	0.8271	1	0.9957
Lipid	Ketone Bodies	3-hydroxybutyrate (BHBA)	HMDB00357	1.75	0.1445	0.83	0.6402
Lipid	Neurotransmitter	acetylcholine	HMDB00895	0.67	0.5419	2.66	0.1516
Lipid	Fatty Acid Metabolism (Acyl Choline)	palmitoylcholine		1.5	0.2667	1.38	0.3921
Lipid	Fatty Acid Metabolism (Acyl Choline)	oleoylcholine		**2.07**	0.0634	**2.17**	0.0568
Lipid	Fatty Acid Metabolism (Acyl Choline)	palmitoloelycholine		1.17	0.1301	**0.74**	0.0070
Lipid	Fatty Acid Metabolism (Acyl Choline)	linoleoylcholine*		**1.85**	0.0459	1.5	0.2118
Lipid	Fatty Acid Metabolism (Acyl Choline)	stearoylcholine*		1.18	0.2793	0.92	0.5906
Lipid	Fatty Acid, Monohydroxy	4-hydroxybutyrate (GHB)	HMDB00710	1.31	0.3641	1.32	0.3721
Lipid	Fatty Acid, Monohydroxy	2-hydroxydecanoate		0.92	0.6194	1	0.9799
Lipid	Fatty Acid, Monohydroxy	2-hydroxypalmitate	HMDB31057	1.06	0.8682	0.88	0.7384
Lipid	Fatty Acid, Monohydroxy	2-hydroxystearate		1.19	0.5186	0.9	0.6972
Lipid	Fatty Acid, Monohydroxy	3-hydroxypropanoate	HMDB00700	1	1.0000	0.91	0.5915

Lipid	Fatty Acid, Monohydroxy	3-hydroxysuberate	HMDB00325	0.81	0.3547	0.83	0.4350
Lipid	Fatty Acid, Monohydroxy	3-hydroxyhexanoate		1.28	0.4908	0.9	0.7714
Lipid	Fatty Acid, Monohydroxy	3-hydroxyoctanoate	HMDB01954	0.97	0.8562	1.01	0.9580
Lipid	Fatty Acid, Monohydroxy	3-hydroxydecanoate	HMDB02203	0.96	0.8622	1.05	0.8125
Lipid	Fatty Acid, Monohydroxy	3-hydroxysebacate	HMDB00350	0.91	0.7490	0.94	0.8516
Lipid	Fatty Acid, Monohydroxy	3-hydroxylaurate	HMDB00387	0.94	0.7528	1.14	0.5134
Lipid	Fatty Acid, Monohydroxy	3-hydroxymyristate		0.94	0.7735	0.96	0.8341
Lipid	Fatty Acid, Monohydroxy	3-hydroxypalmitate	HMDB10734	1.19	0.6388	1.01	0.9723
Lipid	Fatty Acid, Monohydroxy	5-hydroxyhexanoate	HMDB00525	**0.55**	0.0523	0.92	0.8074
Lipid	Fatty Acid, Monohydroxy	5-hydroxydecanoate	HMDB40329	0.95	0.7920	1.09	0.6620
Lipid	Fatty Acid, Monohydroxy	8-hydroxyoctanoate	HMDB61914	0.75	0.1576	**1.61**	0.0224
Lipid	Fatty Acid, Monohydroxy	13-HODE + 9-HODE		1.32	0.4913	1.36	0.4661
Lipid	Fatty Acid, Monohydroxy	14-HDoHE/17-HDoHE		0.87	0.7837	1.85	0.2342
Lipid	Fatty Acid, Monohydroxy	10-hydroxystearate	HMDB37396	0.82	0.4929	1.41	0.2483
Lipid	Fatty Acid, Monohydroxy	3-hydroxystearate		1.36	0.3525	1.24	0.5362
Lipid	Fatty Acid, Monohydroxy	2-hydroxylaurate		1	0.9882	0.77	0.1940
Lipid	Fatty Acid, Monohydroxy	3-hydroxyvalerate	HMDB00531	2.11	0.1131	1.33	0.5630
Lipid	Fatty Acid, Dihydroxy	12,13-DiHOME	HMDB04705	0.89	0.7839	**2.78**	0.0246
Lipid	Fatty Acid, Dihydroxy	9,10-DiHOME	HMDB04704	0.9	0.7816	**3.75**	0.0012
Lipid	Endocannabinoid	oleoyl ethanolamide	HMDB02088	0.84	0.5659	**1.68**	0.0881
Lipid	Endocannabinoid	myristoyl ethanolamide		0.88	0.6254	0.92	0.7693
Lipid	Endocannabinoid	palmitoyl ethanolamide	HMDB02100	0.89	0.6443	1.08	0.7486
Lipid	Endocannabinoid	stearoyl ethanolamide	HMDB13078	0.84	0.4626	1.08	0.7485
Lipid	Endocannabinoid	dihomo-linolenoyl ethanolamide	HMDB13625	1.1	0.7301	0.81	0.4638
Lipid	Endocannabinoid	arachidonoyl ethanolamide	HMDB04080	0.96	0.9331	0.46	0.1109
Lipid	Endocannabinoid	N-oleoyltaurine		0.63	0.2938	1.41	0.4566
Lipid	Endocannabinoid	N-stearoyltaurine		1.16	0.6978	0.98	0.9637
Lipid	Endocannabinoid	N-palmitoyltaurine		0.84	0.7077	1.09	0.8682
Lipid	Endocannabinoid	linoleoyl ethanolamide	HMDB12252	0.86	0.6820	1.22	0.6049
Lipid	Endocannabinoid	arachidoyl ethanolamide (20:0)*		1.03	0.9219	1.29	0.3824
Lipid	Endocannabinoid	behenoyl ethanolamide (22:0)*		1.15	0.6814	1.01	0.9767
Lipid	Endocannabinoid	lignoceroyl ethanolamide (24:0)*		1.24	0.5184	0.88	0.7195
Lipid	Endocannabinoid	palmitoleoyl ethanolamide*	HMDB13648	0.71	0.1952	1	0.9959
Lipid	Endocannabinoid	margaroyl ethanolamide*		0.9	0.6750	1.07	0.8087

Lipid	Endocannabinoid	N-oleoylserine		0.74	0.3353	**1.8**	0.0733
Lipid	Endocannabinoid	N-palmitoylserine		1.79	0.2014	0.65	0.3592
Lipid	Inositol Metabolism	myo-inositol	HMDB00211	0.81	0.6592	**2.69**	0.0526
Lipid	Inositol Metabolism	chiro-inositol	HMDB34220	0.66	0.4460	2.4	0.1245
Lipid	Inositol Metabolism	pinitol	HMDB34219	0.58	0.3614	0.82	0.7475
Lipid	Inositol Metabolism	inositol 1-phosphate (I1P)	HMDB00213	**2.15**	0.0554	0.84	0.6651
Lipid	Phospholipid Metabolism	choline	HMDB00097	**0.64**	0.0858	1.02	0.9402
Lipid	Phospholipid Metabolism	choline phosphate	HMDB01565	1.03	0.9378	1.04	0.9389
Lipid	Phospholipid Metabolism	glycerophosphorylcholine (GPC)	HMDB00086	0.72	0.6045	1.08	0.9117
Lipid	Phospholipid Metabolism	glycerophosphoethanolamine	HMDB00114	1.3	0.6092	0.87	0.8008
Lipid	Phospholipid Metabolism	glycerophosphoserine*		1.22	0.5868	1.23	0.6048
Lipid	Phospholipid Metabolism	glycerophosphoinositol*		1.08	0.8440	0.76	0.5102
Lipid	Phospholipid Metabolism	trimethylamine N-oxide	HMDB00925	1.11	0.7502	0.78	0.4701
Lipid	Phosphatidylcholine (PC)	1,2-dipalmitoyl-GPC (16:0/16:0)	HMDB00564	0.64	0.4054	0.73	0.5839
Lipid	Phosphatidylcholine (PC)	1-palmitoyl-2-stearoyl-GPC (16:0/18:0)	HMDB07970	1.3	0.4483	0.94	0.8672
Lipid	Phosphatidylcholine (PC)	1-palmitoyl-2-oleoyl-GPC (16:0/18:1)	HMDB07972	0.62	0.2562	0.89	0.7825
Lipid	Phosphatidylcholine (PC)	1-palmitoyl-2-linoleoyl-GPC (16:0/18:2)	HMDB07973	0.75	0.4039	1.04	0.9223
Lipid	Phosphatidylcholine (PC)	1-stearoyl-2-oleoyl-GPC (18:0/18:1)	HMDB08038	1	0.9993	1.12	0.6255
Lipid	Lysophospholipid	1-palmitoyl-GPC (16:0)	HMDB10382	0.63	0.4555	0.73	0.6209
Lipid	Lysophospholipid	2-palmitoyl-GPC (16:0)*	HMDB61702	0.93	0.8383	0.78	0.5354
Lipid	Lysophospholipid	1-stearoyl-GPC (18:0)	HMDB10384	0.62	0.4491	0.9	0.8662
Lipid	Lysophospholipid	1-oleoyl-GPC (18:1)	HMDB02815	1.03	0.9559	1	0.9996
Lipid	Lysophospholipid	1-linoleoyl-GPC (18:2)	HMDB10386	1.07	0.8771	1.17	0.7419
Lipid	Lysophospholipid	1-palmitoyl-GPE (16:0)	HMDB11503	1.75	0.1966	1.02	0.9689
Lipid	Lysophospholipid	1-stearoyl-GPE (18:0)	HMDB11130	0.92	0.8812	1.01	0.9846
Lipid	Lysophospholipid	2-stearoyl-GPE (18:0)*	HMDB11129	1.1	0.8268	1.12	0.8074
Lipid	Lysophospholipid	1-oleoyl-GPE (18:1)	HMDB11506	0.85	0.6745	0.98	0.9524
Lipid	Lysophospholipid	1-linoleoyl-GPE (18:2)*	HMDB11507	1.26	0.3214	0.98	0.9309
Lipid	Lysophospholipid	1-palmitoyl-GPS (16:0)*		1.41	0.2138	1.09	0.7760
Lipid	Lysophospholipid	1-stearoyl-GPS (18:0)*		0.99	0.9836	0.88	0.8183
Lipid	Lysophospholipid	1-palmitoyl-GPG (16:0)*		0.89	0.7767	0.82	0.6487
Lipid	Lysophospholipid	1-stearoyl-GPG (18:0)		1.1	0.8316	1	0.9933
Lipid	Lysophospholipid	1-oleoyl-GPG (18:1)*		1.21	0.5622	0.98	0.9417
Lipid	Lysophospholipid	1-linoleoyl-GPG (18:2)*		0.98	0.9236	1.08	0.7553

Lipid	Lysophospholipid	1-palmitoyl-GPI (16:0)	HMDB61695	1.41	0.4974	0.65	0.4037
Lipid	Lysophospholipid	1-stearoyl-GPI (18:0)	HMDB61696	1.09	0.8863	0.67	0.5095
Lipid	Lysophospholipid	1-oleoyl-GPI (18:1)*		0.91	0.7468	0.99	0.9736
Lipid	Lysophospholipid	1-linoleoyl-GPI (18:2)*		**1.36**	0.0724	0.99	0.9419
Lipid	Glycolipid Metabolism	galactosylglycerol*	HMDB06790	1.42	0.5564	0.77	0.6726
Lipid	Glycolipid Metabolism	1-palmitoyl-2-linoleoyl-digalactosylglycerol (16:0/18:2)*		0.72	0.3390	1.46	0.2829
Lipid	Glycolipid Metabolism	1-palmitoyl-2-linoleoyl-galactosylglycerol (16:0/18:2)*		1.07	0.8597	1.43	0.3687
Lipid	Glycolipid Metabolism	1-palmitoyl-2-linolenoyl-galactosylglycerol (16:0/18:3)*		1.28	0.2591	0.99	0.9737
Lipid	Glycolipid Metabolism	1,2-dilinoleoyl-digalactosylglycerol (18:2/18:2)*		0.77	0.4201	0.94	0.8551
Lipid	Glycolipid Metabolism	1,2-dilinoleoyl-galactosylglycerol (18:2/18:2)*		1.43	0.2438	1.27	0.4606
Lipid	Glycolipid Metabolism	1-linoleoyl-2-linolenoyl-galactosylglycerol (18:2/18:3)*		0.93	0.7558	1.09	0.7050
Lipid	Glycolipid Metabolism	1-linoleoyl-2-linolenoyl-digalactosylglycerol (18:2/18:3)*		0.97	0.8943	1.01	0.9561
Lipid	Glycolipid Metabolism	1,2-dilinolenoyl-galactosylglycerol (18:3/18:3)*		0.91	0.7955	0.7	0.3216
Lipid	Lysoplasmalogen	1-(1-enyl-palmitoyl)-GPC (P-16:0)*	HMDB10407	0.8	0.3343	1.03	0.8877
Lipid	Lysoplasmalogen	1-(1-enyl-palmitoyl)-GPE (P-16:0)*		0.95	0.8687	1.13	0.6952
Lipid	Lysoplasmalogen	1-(1-enyl-oleoyl)-GPE (P-18:1)*		1.15	0.6979	1.28	0.5233
Lipid	Lysoplasmalogen	1-(1-enyl-stearoyl)-GPE (P-18:0)*		0.83	0.4964	1.14	0.6212
Lipid	Glycerolipid Metabolism	glycerol	HMDB00131	1.08	0.8403	0.96	0.9258
Lipid	Glycerolipid Metabolism	glycerol 3-phosphate	HMDB00126	1.04	0.9245	0.8	0.5743
Lipid	Glycerolipid Metabolism	glycerophosphoglycerol		0.97	0.9462	0.91	0.8620
Lipid	Monoacylglycerol	1-myristoylglycerol (14:0)	HMDB11561	1.08	0.8363	**0.51**	0.0894
Lipid	Monoacylglycerol	1-pentadecanoylglycerol (15:0)		1.54	0.1293	1.02	0.9447
Lipid	Monoacylglycerol	1-palmitoylglycerol (16:0)	HMDB31074	1.21	0.5590	1.21	0.5765
Lipid	Monoacylglycerol	1-palmitoleoylglycerol (16:1)*	HMDB11565	1.02	0.9362	0.79	0.3335
Lipid	Monoacylglycerol	1-oleoylglycerol (18:1)	HMDB11567	1.33	0.5450	1.64	0.3102
Lipid	Monoacylglycerol	1-linoleoylglycerol (18:2)		1.6	0.3964	2.35	0.1443
Lipid	Monoacylglycerol	1-linolenoylglycerol (18:3)	HMDB11569	**2.36**	0.0457	1.01	0.9871
Lipid	Monoacylglycerol	2-palmitoylglycerol (16:0)	HMDB11533	1.02	0.9524	1.22	0.6155
Lipid	Monoacylglycerol	2-oleoylglycerol (18:1)	HMDB11537	1.01	0.9848	0.96	0.9377
Lipid	Monoacylglycerol	2-linoleoylglycerol (18:2)	HMDB11538	1.01	0.9779	1.75	0.2884

Lipid	Diacylglycerol	palmitoyl-oleoyl-glycerol (16:0/18:1) [2]*	HMDB07102	**1.3**	0.0912	0.87	0.4012
Lipid	Diacylglycerol	palmitoyl-linoleoyl-glycerol (16:0/18:2) [1]*	HMDB07103	1.19	0.4401	1.12	0.6269
Lipid	Diacylglycerol	palmitoyl-linoleoyl-glycerol (16:0/18:2) [2]*	HMDB07103	1.62	0.1274	1.19	0.6012
Lipid	Diacylglycerol	oleoyl-oleoyl-glycerol (18:1/18:1) [2]*	HMDB07218	**1.55**	0.0901	0.71	0.2113
Lipid	Diacylglycerol	oleoyl-linoleoyl-glycerol (18:1/18:2) [1]	HMDB07219	1.54	0.3074	**2.21**	0.0778
Lipid	Diacylglycerol	oleoyl-linoleoyl-glycerol (18:1/18:2) [2]	HMDB07219	**2.43**	0.0932	2.1	0.1811
Lipid	Diacylglycerol	linoleoyl-linoleoyl-glycerol (18:2/18:2) [1]*	HMDB07248	2.42	0.1319	2.12	0.2236
Lipid	Diacylglycerol	linoleoyl-linoleoyl-glycerol (18:2/18:2) [2]*	HMDB07248	1.9	0.1856	1.35	0.5510
Lipid	Diacylglycerol	linoleoyl-linolenoyl-glycerol (18:2/18:3) [1]*	HMDB07249	**2.04**	0.0285	1.18	0.6201
Lipid	Diacylglycerol	linoleoyl-linolenoyl-glycerol (18:2/18:3) [2]*	HMDB07250	**2.33**	0.0665	1.31	0.5719
Lipid	Diacylglycerol	linolenoyl-linolenoyl-glycerol (18:3/18:3) [1]*	HMDB07278	1.31	0.1037	0.93	0.7007
Lipid	Diacylglycerol	linolenoyl-linolenoyl-glycerol (18:3/18:3) [2]*	HMDB07278	**2.29**	0.0404	0.76	0.5121
Lipid	Diacylglycerol	oleoyl-arachidonoyl-glycerol (18:1/20:4) [2]*	HMDB07228	1.21	0.1904	0.82	0.1850
Lipid	Diacylglycerol	linoleoyl-arachidonoyl-glycerol (18:2/20:4) [1]*	HMDB07257	1.27	0.1361	**0.73**	0.0596
Lipid	Diacylglycerol	linoleoyl-arachidonoyl-glycerol (18:2/20:4) [2]*	HMDB07257	1.22	0.4137	0.73	0.2139
Lipid	Diacylglycerol	linoleoyl-docosahexaenoyl-glycerol (18:2/22:6) [1]*		1.03	0.8045	**0.66**	0.0006
Lipid	Diacylglycerol	linoleoyl-docosahexaenoyl-glycerol (18:2/22:6) [2]*	HMDB07266	0.98	0.9291	**0.59**	0.0165
Lipid	Sphingolipid Metabolism	sphinganine	HMDB00269	0.92	0.7653	0.84	0.5424
Lipid	Sphingolipid Metabolism	3-ketosphinganine	HMDB01480	1.9	0.1885	0.63	0.3693
Lipid	Sphingolipid Metabolism	N-palmitoyl-sphinganine (d18:0/16:0)	HMDB11760	1.04	0.8507	0.87	0.5506
Lipid	Sphingolipid Metabolism	N-behenoyl-sphingadienine (d18:2/22:0)*		0.39	0.1251	1.57	0.4801
Lipid	Sphingolipid Metabolism	galactosylsphingosine	HMDB00648	0.59	0.2617	1.3	0.5801
Lipid	Sphingolipid Metabolism	N-butyroyl-sphingosine (d18:1/4:0)		1.16	0.7318	1.85	0.1743
Lipid	Sphingolipid Metabolism	myristoyl dihydrosphingomyelin (d18:0/14:0)*	HMDB12085	0.73	0.4668	0.64	0.3248
Lipid	Sphingolipid Metabolism	palmitoyl dihydrosphingomyelin (d18:0/16:0)*		0.67	0.2319	0.9	0.7480
Lipid	Sphingolipid Metabolism	behenoyl dihydrosphingomyelin (d18:0/22:0)*	HMDB12091	0.52	0.1492	1.21	0.6869
Lipid	Sphingolipid Metabolism	palmitoyl sphingomyelin (d18:1/16:0)		0.63	0.3262	0.77	0.5759
Lipid	Sphingolipid Metabolism	stearoyl sphingomyelin (d18:1/18:0)	HMDB01348	0.45	0.1522	0.84	0.7606
Lipid	Sphingolipid Metabolism	behenoyl sphingomyelin (d18:1/22:0)*	HMDB12103	0.4	0.1171	0.71	0.5659
Lipid	Sphingolipid Metabolism	tricosanoyl sphingomyelin (d18:1/23:0)*	HMDB12105	0.58	0.2786	1.21	0.7108
Lipid	Sphingolipid Metabolism	lignoceroyl sphingomyelin (d18:1/24:0)		0.65	0.4197	0.83	0.7365
Lipid	Sphingolipid Metabolism	sphingomyelin (d18:1/14:0, d16:1/16:0)*	HMDB12097	0.61	0.3129	0.83	0.7019

Lipid	Sphingolipid Metabolism	sphingomyelin (d17:1/16:0, d18:1/15:0, d16:1/17:0)*		0.82	0.6381	**2.17**	0.0779
Lipid	Sphingolipid Metabolism	sphingomyelin (d18:2/16:0, d18:1/16:1)*		0.77	0.4471	1.01	0.9693
Lipid	Sphingolipid Metabolism	sphingomyelin (d18:1/17:0, d17:1/18:0, d19:1/16:0)		0.77	0.5009	1.61	0.2247
Lipid	Sphingolipid Metabolism	sphingomyelin (d18:1/18:1, d18:2/18:0)	HMDB12101	0.66	0.1617	1.07	0.8182
Lipid	Sphingolipid Metabolism	sphingomyelin (d18:1/20:0, d16:1/22:0)*	HMDB12102	0.55	0.2814	0.76	0.6295
Lipid	Sphingolipid Metabolism	sphingomyelin (d18:1/20:1, d18:2/20:0)*		**0.62**	0.0930	0.77	0.3640
Lipid	Sphingolipid Metabolism	sphingomyelin (d18:1/21:0, d17:1/22:0, d16:1/23:0)*		0.59	0.1078	0.86	0.6522
Lipid	Sphingolipid Metabolism	sphingomyelin (d18:1/22:1, d18:2/22:0, d16:1/24:1)*	HMDB12104	0.76	0.5474	1.02	0.9694
Lipid	Sphingolipid Metabolism	sphingomyelin (d18:2/23:0, d18:1/23:1, d17:1/24:1)*		0.73	0.1276	0.94	0.7579
Lipid	Sphingolipid Metabolism	sphingomyelin (d18:1/24:1, d18:2/24:0)*	HMDB12107	0.49	0.2338	0.86	0.7977
Lipid	Sphingolipid Metabolism	sphingomyelin (d18:2/24:1, d18:1/24:2)*		0.87	0.7665	0.92	0.8624
Lipid	Sphingolipid Metabolism	sphingosine	HMDB00252	0.81	0.3437	1	0.9882
Lipid	Sphingolipid Metabolism	N-acetylsphingosine	HMDB04950	1.11	0.7987	**2.44**	0.0387
Lipid	Sphingolipid Metabolism	phytosphingosine	HMDB04610	0.78	0.2630	**1.51**	0.0650
Lipid	Sphingolipid Metabolism	sphingomyelin (d18:0/20:0, d16:0/22:0)*		0.7	0.2969	0.74	0.3770
Lipid	Sphingolipid Metabolism	sphingomyelin (d18:0/18:0, d19:0/17:0)*	HMDB12087	**0.48**	0.0751	0.87	0.7381
Lipid	Sphingolipid Metabolism	sphingomyelin (d18:1/19:0, d19:1/18:0)*		0.73	0.1570	0.9	0.6403
Lipid	Sphingolipid Metabolism	heptadecasphingosine (d17:1)		0.76	0.4221	1.07	0.8416
Lipid	Sphingolipid Metabolism	hexadecasphingosine (d16:1)*		0.86	0.6481	1.08	0.8135
Lipid	Sphingolipid Metabolism	N-palmitoyl-heptadecasphingosine (d17:1/16:0)*		1.02	0.9474	0.88	0.6397
Lipid	Sphingolipid Metabolism	N-stearoyl-sphinganine (d18:0/18:0)*		0.95	0.8354	0.89	0.6923
Lipid	Sphingolipid Metabolism	lactosyl-N-behenoyl-sphingosine (d18:1/22:0)*		0.91	0.8299	0.7	0.4428
Lipid	Sphingolipid Metabolism	hexadecasphinganine (d16:0)*		1.16	0.7215	0.7	0.4117
Lipid	Sphingolipid Metabolism	lactosyl-N-arachidoyl-sphingosine (d18:1/20:0)*		0.77	0.5216	0.81	0.5996
Lipid	Sphingolipid Metabolism	N-(2-hydroxypalmitoyl)-sphingosine (d18:1/16:0(2OH))		0.98	0.9540	1.11	0.7528
Lipid	Sphingolipid Metabolism	N-oleoyl-sphingosine (d18:1/18:1)*	HMDB04948	0.66	0.2016	1.07	0.8468
Lipid	Sphingolipid Metabolism	eicosanoylsphingosine (d20:1)*		0.87	0.5485	0.98	0.9359
Lipid	Ceramides	N-palmitoyl-sphingosine (d18:1/16:0)	HMDB04949	1	0.9973	0.82	0.3655
Lipid	Ceramides	N-stearoyl-sphingosine (d18:1/18:0)*	HMDB04950	0.79	0.3811	0.89	0.6705
Lipid	Ceramides	ceramide (d16:1/24:1, d18:1/22:1)*		1.03	0.9520	0.54	0.2239
Lipid	Ceramides	ceramide (d18:1/14:0, d16:1/16:0)*		1.03	0.9195	0.76	0.2937
Lipid	Ceramides	ceramide (d18:1/17:0, d17:1/18:0)*		1	0.9929	0.89	0.6459
Lipid	Ceramides	ceramide (d18:2/24:1, d18:1/24:2)*		0.93	0.8008	0.82	0.5114

Lipid	Ceramides	glycosyl-N-palmitoyl-sphingosine (d18:1/16:0)		0.88	0.6698	0.95	0.8727
Lipid	Ceramides	glycosyl-N-stearoyl-sphingosine (d18:1/18:0)		0.58	0.1520	1.26	0.5561
Lipid	Ceramides	glycosyl-N-behenoyl-sphingadienine (d18:2/22:0)*		0.6	0.2354	1.18	0.7004
Lipid	Ceramides	glycosyl-N-(2-hydroxynervonoyl)-sphingosine (d18:1/24:1(2OH))*		0.91	0.7439	1.48	0.1962
Lipid	Ceramides	lactosyl-N-palmitoyl-sphingosine (d18:1/16:0)		0.9	0.7968	0.64	0.2856
Lipid	Ceramides	lactosyl-N-stearoyl-sphingosine (d18:1/18:0)*	HMDB11591	0.58	0.2668	0.73	0.5342
Lipid	Ceramides	lactosyl-N-nervonoyl-sphingosine (d18:1/24:1)*		0.92	0.8640	0.5	0.1555
Lipid	Ceramides	glycosyl ceramide (d16:1/24:1, d18:1/22:1)*		0.76	0.4292	0.72	0.3617
Lipid	Ceramides	glycosyl ceramide (d18:1/20:0, d16:1/22:0)*		0.65	0.2589	1.27	0.5299
Lipid	Ceramides	glycosyl ceramide (d18:2/24:1, d18:1/24:2)*		0.75	0.4919	0.92	0.8346
Lipid	Mevalonate Metabolism	3-hydroxy-3-methylglutarate	HMDB00355	0.74	0.4206	**2.77**	0.0098
Lipid	Mevalonate Metabolism	mevalonate	HMDB00227	1.46	0.3525	0.54	0.1372
Lipid	Mevalonate Metabolism	mevalonolactone	HMDB06024	1.08	0.7964	0.65	0.1611
Lipid	Sterol	lanosterol	HMDB01251	0.74	0.3366	1.19	0.5842
Lipid	Sterol	desmosterol	HMDB02719	**0.48**	0.0780	1	0.9945
Lipid	Sterol	cholesterol	HMDB00067	0.73	0.2720	1.03	0.9326
Lipid	Sterol	3beta-hydroxy-5-cholestenoate		1.13	0.7122	1.05	0.8872
Lipid	Sterol	coprostanol	HMDB00577	1.08	0.7510	0.76	0.2509
Lipid	Sterol	4-cholesten-3-one	HMDB00921	0.71	0.3356	0.79	0.5203
Lipid	Sterol	beta-sitosterol	HMDB00852	0.77	0.3699	**2.89**	0.0006
Lipid	Sterol	stigmasterol	HMDB00937	1.14	0.6191	**2.01**	0.0159
Lipid	Sterol	campesterol	HMDB02869	0.86	0.6689	**2.1**	0.0415
Lipid	Sterol	7-hydroxycholesterol (alpha or beta)	HMDB06119	0.81	0.4562	0.85	0.5864
Lipid	Pregnenolone Steroids	pregnenolone sulfate	HMDB00774	0.8	0.5656	0.86	0.7158
Lipid	Pregnenolone Steroids	17alpha-hydroxypregnenolone 3-sulfate	HMDB00416	1.15	0.6368	0.67	0.1965
Lipid	Pregnenolone Steroids	21-hydroxypregnenolone monosulfate (1)		0.78	0.1765	0.76	0.1549
Lipid	Pregnenolone Steroids	21-hydroxypregnenolone disulfate		0.79	0.6159	1.12	0.8150
Lipid	Pregnenolone Steroids	21-hydroxypregnanolone disulfate		0.91	0.8199	1.47	0.3710
Lipid	Pregnenolone Steroids	pregnenediol disulfate (C21H34O8S2)*		0.75	0.6166	0.88	0.8293
Lipid	Pregnenolone Steroids	pregnenediol sulfate (C21H34O5S)*		0.96	0.9322	0.87	0.7832
Lipid	Progestin Steroids	5alpha-pregnan-3beta-ol,20-one sulfate		1.36	0.2800	0.91	0.7620
Lipid	Progestin Steroids	5alpha-pregnan-3beta,20beta-diol monosulfate (1)		0.56	0.1447	0.59	0.1764

Lipid	Progestin Steroids	5alpha-pregnan-3beta,20alpha-diol monosulfate (2)		0.89	0.6558	0.79	0.3978
Lipid	Progestin Steroids	5alpha-pregnan-3beta,20alpha-diol disulfate		0.67	0.2903	0.83	0.6302
Lipid	Progestin Steroids	5alpha-pregnan-diol disulfate		0.61	0.2676	0.57	0.2174
Lipid	Progestin Steroids	pregnanolone/allopregnanolone sulfate		**0.38**	0.0640	**0.36**	0.0564
Lipid	Corticosteroids	tetrahydrocorticosterone	HMDB00268	0.65	0.1965	0.71	0.3195
Lipid	Corticosteroids	cortisone 21-sulfate	HMDB02802	0.74	0.1949	0.81	0.3731
Lipid	Corticosteroids	cortolone	HMDB03128	0.81	0.4222	0.96	0.8866
Lipid	Androgenic Steroids	11-ketoetiocholanolone sulfate		**0.57**	0.0625	0.61	0.1135
Lipid	Androgenic Steroids	dehydroisoandrosterone sulfate (DHEA-S)	HMDB01032	0.88	0.7183	0.83	0.5930
Lipid	Androgenic Steroids	16a-hydroxy DHEA 3-sulfate		0.93	0.8511	0.83	0.6574
Lipid	Androgenic Steroids	androsterone sulfate	HMDB02759	0.72	0.2540	0.75	0.3111
Lipid	Androgenic Steroids	5alpha-androstan-3alpha,17alpha-diol monosulfate		0.92	0.7385	0.77	0.2800
Lipid	Androgenic Steroids	5alpha-androstan-3alpha,17alpha-diol disulfate		**0.56**	0.0199	**0.63**	0.0625
Lipid	Androgenic Steroids	androstenediol (3beta,17beta) monosulfate (1)	HMDB03818	0.95	0.8934	0.88	0.7215
Lipid	Androgenic Steroids	androstenediol (3beta,17beta) disulfate (1)	HMDB03818	0.83	0.5938	0.78	0.4899
Lipid	Androgenic Steroids	androstenediol (3beta,17beta) disulfate (2)	HMDB03818	0.71	0.2185	**0.54**	0.0324
Lipid	Androgenic Steroids	androstenediol (3alpha, 17alpha) monosulfate (2)		0.74	0.1809	0.91	0.6905
Lipid	Androgenic Steroids	5alpha-androstan-3alpha,17beta-diol monosulfate (1)		0.78	0.1059	1.06	0.7224
Lipid	Androgenic Steroids	andro steroid monosulfate C19H28O6S (1)*	HMDB02759	0.92	0.8125	0.63	0.1893
Lipid	Primary Bile Acid Metabolism	cholate	HMDB00619	0.66	0.3826	2.1	0.1315
Lipid	Primary Bile Acid Metabolism	glycocholate	HMDB00138	**0.39**	0.0483	1.37	0.5237
Lipid	Primary Bile Acid Metabolism	taurocholate	HMDB00036	0.46	0.1607	1.03	0.9555
Lipid	Primary Bile Acid Metabolism	chenodeoxycholate	HMDB00518	0.66	0.4203	2.37	0.1106
Lipid	Primary Bile Acid Metabolism	glycochenodeoxycholate	HMDB00637	**0.43**	0.0434	0.64	0.3052
Lipid	Primary Bile Acid Metabolism	taurochenodeoxycholate	HMDB00951	**0.39**	0.0723	1.15	0.7978
Lipid	Primary Bile Acid Metabolism	cholate sulfate		**3.53**	0.0826	0.3	0.1151
Lipid	Primary Bile Acid Metabolism	glycochenodeoxycholate glucuronide (2)		**0.37**	0.0195	0.78	0.5717
Lipid	Primary Bile Acid Metabolism	glycochenodeoxycholate sulfate		0.57	0.2283	**0.35**	0.0284
Lipid	Primary Bile Acid Metabolism	glycocholate sulfate		0.79	0.3746	1.09	0.7545
Lipid	Secondary Bile Acid Metabolism	deoxycholate	HMDB00626	0.54	0.2274	0.58	0.2907
Lipid	Secondary Bile Acid Metabolism	deoxycholic acid sulfate		0.86	0.7467	0.97	0.9500
Lipid	Secondary Bile Acid Metabolism	glycodeoxycholate	HMDB00631	0.89	0.6476	0.99	0.9742

Lipid	Secondary Bile Acid Metabolism	lithocholate	HMDB00761	0.38	0.2770	0.37	0.2654
Lipid	Secondary Bile Acid Metabolism	taurolithocholate	HMDB00722	0.8	0.1088	0.95	0.7055
Lipid	Secondary Bile Acid Metabolism	taurolithocholate 3-sulfate	HMDB02580	0.53	0.1568	1.18	0.7193
Lipid	Secondary Bile Acid Metabolism	ursodeoxycholate	HMDB00946	1.04	0.9335	2.12	0.1197
Lipid	Secondary Bile Acid Metabolism	isoursodeoxycholate	HMDB00686	0.97	0.9420	1.36	0.5204
Lipid	Secondary Bile Acid Metabolism	glycoursodeoxycholate	HMDB00708	0.63	0.3240	0.76	0.5662
Lipid	Secondary Bile Acid Metabolism	tauroursodeoxycholate	HMDB00874	0.76	0.5337	1.01	0.9888
Lipid	Secondary Bile Acid Metabolism	dehydrolithocholate	HMDB00502	0.73	0.6045	0.58	0.3740
Lipid	Secondary Bile Acid Metabolism	7,12-diketolithocholate		0.82	0.7124	0.95	0.9337
Lipid	Secondary Bile Acid Metabolism	6-oxolithocholate		0.72	0.6198	0.77	0.6937
Lipid	Secondary Bile Acid Metabolism	7-ketolithocholate	HMDB00467	0.88	0.7689	1.41	0.4550
Lipid	Secondary Bile Acid Metabolism	hyocholate	HMDB00760	0.68	0.2826	1.59	0.2063
Lipid	Secondary Bile Acid Metabolism	dehydrocholic acid		0.96	0.9185	0.63	0.3045
Lipid	Secondary Bile Acid Metabolism	3-dehydrocholate	HMDB00502	0.49	0.1949	**2.84**	0.0656
Lipid	Secondary Bile Acid Metabolism	12-dehydrocholate	HMDB00400	**0.39**	0.0508	2.25	0.1121
Lipid	Secondary Bile Acid Metabolism	glycocholenate sulfate*		0.79	0.6988	1.28	0.7024
Lipid	Secondary Bile Acid Metabolism	taurocholenate sulfate		0.62	0.4856	2.2	0.2639
Lipid	Secondary Bile Acid Metabolism	7-ketodeoxycholate	HMDB00391	0.81	0.6536	1.5	0.4026
Lipid	Secondary Bile Acid Metabolism	7alpha-hydroxycholestenone	HMDB01993	**0.64**	0.0411	0.88	0.5716
Lipid	Secondary Bile Acid Metabolism	3b-hydroxy-5-cholenoic acid	HMDB00308	1.11	0.7139	0.78	0.4018
Lipid	Secondary Bile Acid Metabolism	ursodeoxycholate sulfate (1)		0.98	0.9763	0.37	0.1587
Lipid	Secondary Bile Acid Metabolism	ursocholate		1.17	0.7867	2.3	0.1652
Nucleotide	Purine Metabolism, (Hypo)Xanthine/Inosine containing	inosine	HMDB00195	0.94	0.9058	0.84	0.7682
Nucleotide	Purine Metabolism, (Hypo)Xanthine/Inosine containing	hypoxanthine	HMDB00157	0.73	0.2458	1.22	0.4738
Nucleotide	Purine Metabolism, (Hypo)Xanthine/Inosine containing	xanthine	HMDB00292	0.9	0.5782	0.94	0.7652
Nucleotide	Purine Metabolism, (Hypo)Xanthine/Inosine containing	xanthosine	HMDB00299	0.68	0.3536	1.46	0.3641
Nucleotide	Purine Metabolism, (Hypo)Xanthine/Inosine containing	2'-deoxyinosine	HMDB00071	0.53	0.3082	2.76	0.1210
Nucleotide	Purine Metabolism, (Hypo)Xanthine/Inosine containing	urate	HMDB00289	0.78	0.6366	1.2	0.7409
Nucleotide	Purine Metabolism, (Hypo)Xanthine/Inosine containing	allantoin	HMDB00462	0.64	0.2900	1.49	0.3523
Nucleotide	Purine Metabolism, (Hypo)Xanthine/Inosine containing	allantoic acid	HMDB01209	0.66	0.2929	1.07	0.8709

Nucleotide	Purine Metabolism, Adenine containing	adenosine	HMDB00050	0.98	0.9572	1.51	0.2550
Nucleotide	Purine Metabolism, Adenine containing	adenine	HMDB00034	0.86	0.6977	1.68	0.2080
Nucleotide	Purine Metabolism, Adenine containing	1-methyladenine	HMDB11599	1.05	0.8425	0.81	0.4123
Nucleotide	Purine Metabolism, Adenine containing	N6-dimethylallyladenine		0.94	0.8249	**0.28**	0.0000
Nucleotide	Purine Metabolism, Adenine containing	N6-carbamoylthreonyladenosine	HMDB41623	1.07	0.7292	1	1.0000
Nucleotide	Purine Metabolism, Adenine containing	2'-deoxyadenosine	HMDB00101	1.19	0.7249	2.21	0.1266
Nucleotide	Purine Metabolism, Guanine containing	guanosine-2',3'-cyclic monophosphate	HMDB11629	1.03	0.8840	**1.51**	0.0655
Nucleotide	Purine Metabolism, Guanine containing	guanosine	HMDB00133	0.81	0.7435	**3.02**	0.0886
Nucleotide	Purine Metabolism, Guanine containing	guanine	HMDB00132	**0.47**	0.0876	**3.22**	0.0112
Nucleotide	Purine Metabolism, Guanine containing	1-methylguanine	HMDB03282	**0.54**	0.0748	1.51	0.2487
Nucleotide	Purine Metabolism, Guanine containing	7-methylguanine	HMDB00897	0.9	0.7969	1.55	0.2797
Nucleotide	Purine Metabolism, Guanine containing	2'-O-methylguanosine		0.84	0.2864	0.97	0.8539
Nucleotide	Purine Metabolism, Guanine containing	N2-methylguanosine	HMDB05862	0.82	0.2140	1	1.0000
Nucleotide	Purine Metabolism, Guanine containing	8-hydroxyguanine	HMDB02032	0.7	0.5426	1.75	0.3501
Nucleotide	Purine Metabolism, Guanine containing	8-hydroxy-2'-deoxyguanosine	HMDB03333	0.96	0.9033	0.99	0.9820
Nucleotide	Purine Metabolism, Guanine containing	2'-deoxyguanosine	HMDB00085	0.64	0.2531	1.25	0.5809
Nucleotide	Pyrimidine Metabolism, Orotate containing	N-carbamoylaspartate	HMDB00828	1.14	0.5814	**0.59**	0.0326
Nucleotide	Pyrimidine Metabolism, Orotate containing	dihydroorotate	HMDB03349	1.89	0.2513	1.4	0.5595
Nucleotide	Pyrimidine Metabolism, Orotate containing	orotate	HMDB00226	1.27	0.4799	0.93	0.8333
Nucleotide	Pyrimidine Metabolism, Orotate containing	orotidine	HMDB00788	0.55	0.4108	2.98	0.1450
Nucleotide	Pyrimidine Metabolism, Uracil containing	uridine-2',3'-cyclic monophosphate	HMDB11640	1.27	0.3081	**1.75**	0.0243
Nucleotide	Pyrimidine Metabolism, Uracil containing	uridine	HMDB00296	1.13	0.7916	1.93	0.1647
Nucleotide	Pyrimidine Metabolism, Uracil containing	uracil	HMDB00300	0.96	0.8453	0.9	0.6448
Nucleotide	Pyrimidine Metabolism, Uracil containing	pseudouridine	HMDB00767	0.81	0.4740	0.87	0.6483
Nucleotide	Pyrimidine Metabolism, Uracil containing	2'-O-methyluridine		0.69	0.1875	0.9	0.7055

Nucleotide	Pyrimidine Metabolism, Uracil containing	5-methyluridine (ribothymidine)	HMDB00884	0.84	0.6936	1.07	0.8784
Nucleotide	Pyrimidine Metabolism, Uracil containing	5,6-dihydrouracil	HMDB00076	1.05	0.9167	1.39	0.5116
Nucleotide	Pyrimidine Metabolism, Uracil containing	2'-deoxyuridine	HMDB00012	1	0.9899	0.75	0.4019
Nucleotide	Pyrimidine Metabolism, Uracil containing	4-ureidobutyrate		0.6	0.2206	1.07	0.8824
Nucleotide	Pyrimidine Metabolism, Uracil containing	3-ureidoisobutyrate	HMDB02031	0.98	0.9574	0.54	0.1939
Nucleotide	Pyrimidine Metabolism, Uracil containing	3-ureidopropionate	HMDB00026	1.14	0.7789	0.64	0.3526
Nucleotide	Pyrimidine Metabolism, Uracil containing	beta-alanine	HMDB00056	1.26	0.5585	0.94	0.8729
Nucleotide	Pyrimidine Metabolism, Uracil containing	N-acetyl-beta-alanine		0.77	0.5149	1.38	0.4493
Nucleotide	Pyrimidine Metabolism, Cytidine containing	cytidine 2',3'-cyclic monophosphate	HMDB11691	1.43	0.4104	1.93	0.1594
Nucleotide	Pyrimidine Metabolism, Cytidine containing	cytidine	HMDB00089	0.85	0.6812	1.06	0.8888
Nucleotide	Pyrimidine Metabolism, Cytidine containing	cytosine	HMDB00630	0.76	0.5880	1.38	0.5243
Nucleotide	Pyrimidine Metabolism, Cytidine containing	2'-deoxycytidine	HMDB00014	0.71	0.4040	0.86	0.7239
Nucleotide	Pyrimidine Metabolism, Thymine containing	thymidine	HMDB00273	0.96	0.9088	1.13	0.7010
Nucleotide	Pyrimidine Metabolism, Thymine containing	thymine	HMDB00262	0.9	0.6576	0.93	0.7613
Nucleotide	Pyrimidine Metabolism, Thymine containing	5,6-dihydrothymine	HMDB00079	0.99	0.9825	1.08	0.8696
Nucleotide	Pyrimidine Metabolism, Thymine containing	3-aminoisobutyrate	HMDB03911	1.23	0.6961	**0.27**	0.0175
Nucleotide	Purine and Pyrimidine Metabolism	methylphosphate	HMDB61711	1.52	0.3528	1.41	0.4602
Cofactors and Vitamins	Nicotinate and Nicotinamide Metabolism	quinolinate	HMDB00232	0.88	0.7272	**0.44**	0.0313
Cofactors and Vitamins	Nicotinate and Nicotinamide Metabolism	nicotinate	HMDB01488	1.15	0.3958	**1.6**	0.0053
Cofactors and Vitamins	Nicotinate and Nicotinamide Metabolism	nicotinate ribonucleoside	HMDB06809	1.22	0.5553	1.19	0.6008
Cofactors and Vitamins	Nicotinate and Nicotinamide Metabolism	nicotinamide	HMDB01406	0.96	0.8468	0.88	0.5517

Cofactors and Vitamins	Nicotinate and Nicotinamide Metabolism	nicotinamide riboside	HMDB00855	1.22	0.7072	0.59	0.3186
Cofactors and Vitamins	Nicotinate and Nicotinamide Metabolism	1-methylnicotinamide	HMDB00699	1	0.9885	1.09	0.8317
Cofactors and Vitamins	Nicotinate and Nicotinamide Metabolism	6-hydroxynicotinate	HMDB02658	0.81	0.6362	1.98	0.1440
Cofactors and Vitamins	Nicotinate and Nicotinamide Metabolism	trigonelline (N'-methylnicotinate)	HMDB00875	0.86	0.6859	0.61	0.1842
Cofactors and Vitamins	Nicotinate and Nicotinamide Metabolism	N1-Methyl-2-pyridone-5-carboxamide	HMDB04193	0.84	0.6932	0.8	0.6201
Cofactors and Vitamins	Riboflavin Metabolism	riboflavin (Vitamin B2)	HMDB00244	1.12	0.6689	1.3	0.3478
Cofactors and Vitamins	Pantothenate and CoA Metabolism	pantothenate	HMDB00210	1.02	0.9262	1.06	0.7748
Cofactors and Vitamins	Ascorbate and Aldarate Metabolism	threonate	HMDB00943	0.58	0.1748	1.41	0.4148
Cofactors and Vitamins	Ascorbate and Aldarate Metabolism	oxalate (ethanedioate)	HMDB02329	1.59	0.1511	1.2	0.5830
Cofactors and Vitamins	Ascorbate and Aldarate Metabolism	gulonate*	HMDB03290	1.28	0.7074	**0.3**	0.0747
Cofactors and Vitamins	Tocopherol Metabolism	alpha-tocopherol	HMDB01893	0.67	0.2817	1.25	0.5562
Cofactors and Vitamins	Tocopherol Metabolism	alpha-tocopherol acetate	HMDB34227	1.1	0.7661	0.95	0.8804
Cofactors and Vitamins	Tocopherol Metabolism	delta-tocopherol	HMDB02902	0.66	0.3030	1.74	0.1867
Cofactors and Vitamins	Tocopherol Metabolism	alpha-tocotrienol	HMDB06327	0.69	0.3922	**3.01**	0.0168
Cofactors and Vitamins	Tocopherol Metabolism	gamma-tocotrienol	HMDB12958	0.67	0.1909	**2.53**	0.0044
Cofactors and Vitamins	Tocopherol Metabolism	gamma-CEHC	HMDB01931	0.84	0.5742	1.45	0.2479
Cofactors and Vitamins	Tocopherol Metabolism	gamma-CEHC glucuronide*		**0.41**	0.0035	0.96	0.8862
Cofactors and Vitamins	Tocopherol Metabolism	alpha-CEHC sulfate		0.85	0.7430	1.16	0.7833
Cofactors and Vitamins	Tocopherol Metabolism	alpha-CEHC	HMDB01518	1.17	0.4215	0.95	0.7928
Cofactors and Vitamins	Tocopherol Metabolism	gamma-tocopherol/beta-tocopherol		0.6	0.1370	1.58	0.1987
Cofactors and Vitamins	Biotin Metabolism	biotin	HMDB00030	1.15	0.6334	1.17	0.6031
Cofactors and Vitamins	Biotin Metabolism	biocytin		0.92	0.7880	1.12	0.7250
Cofactors and Vitamins	Tetrahydrobiopterin Metabolism	biopterin	HMDB00468	0.98	0.9630	0.6	0.2419

Cofactors and Vitamins	Pterin Metabolism	isoxanthopterin	HMDB00704	0.64	0.3937	0.95	0.9262
Cofactors and Vitamins	Pterin Metabolism	pterin	HMDB00802	0.92	0.7667	0.82	0.5110
Cofactors and Vitamins	Hemoglobin and Porphyrin Metabolism	protoporphyrin IX	HMDB00241	2.11	0.1471	1.2	0.7309
Cofactors and Vitamins	Hemoglobin and Porphyrin Metabolism	bilirubin (Z,Z)	HMDB00054	1.19	0.7312	1.26	0.6548
Cofactors and Vitamins	Hemoglobin and Porphyrin Metabolism	bilirubin (E,E)*		0.87	0.7219	1.36	0.4458
Cofactors and Vitamins	Hemoglobin and Porphyrin Metabolism	bilirubin (E,Z or Z,E)*	HMDB00488	1.07	0.8111	1.63	0.1077
Cofactors and Vitamins	Hemoglobin and Porphyrin Metabolism	biliverdin	HMDB01008	0.9	0.7848	0.9	0.7772
Cofactors and Vitamins	Hemoglobin and Porphyrin Metabolism	I-urobilinogen	HMDB04157	0.96	0.9348	1.07	0.8931
Cofactors and Vitamins	Hemoglobin and Porphyrin Metabolism	D-urobilin	HMDB04161	1.28	0.6174	1.02	0.9689
Cofactors and Vitamins	Hemoglobin and Porphyrin Metabolism	L-urobilin	HMDB04159	0.86	0.7429	0.73	0.5097
Cofactors and Vitamins	Hemoglobin and Porphyrin Metabolism	mesobilirubin		1.23	0.6486	1.58	0.3229
Cofactors and Vitamins	Thiamine Metabolism	thiamin (Vitamin B1)	HMDB00235	1.17	0.7732	0.76	0.6255
Cofactors and Vitamins	Thiamine Metabolism	5-(2-Hydroxyethyl)-4-methylthiazole		1.14	0.8126	1.16	0.8009
Cofactors and Vitamins	Thiamine Metabolism	hydroxymethylpyrimidine		0.83	0.6784	1.18	0.7248
Cofactors and Vitamins	Vitamin A Metabolism	retinol (Vitamin A)	HMDB00305	0.86	0.4848	0.95	0.8181
Cofactors and Vitamins	Vitamin A Metabolism	carotene diol (1)		0.67	0.3085	0.84	0.6577
Cofactors and Vitamins	Vitamin A Metabolism	carotene diol (2)		0.79	0.4863	0.69	0.2912
Cofactors and Vitamins	Vitamin A Metabolism	carotene diol (3)		1.02	0.9420	0.82	0.4935
Cofactors and Vitamins	Vitamin A Metabolism	beta-cryptoxanthin	HMDB33844	0.99	0.9720	1.09	0.7715
Cofactors and Vitamins	Vitamin A Metabolism	retinal	HMDB01358	0.93	0.5920	1	0.9823
Cofactors and Vitamins	Vitamin B6 Metabolism	pyridoxine (Vitamin B6)	HMDB02075	1.58	0.3051	**4.65**	0.0011
Cofactors and Vitamins	Vitamin B6 Metabolism	pyridoxamine	HMDB01431	1.06	0.8465	0.97	0.9033
Cofactors and Vitamins	Vitamin B6 Metabolism	pyridoxal	HMDB01545	0.84	0.5038	0.92	0.7545

Cofactors and Vitamins	Vitamin B6 Metabolism	pyridoxate	HMDB00017	0.86	0.5441	2.34	0.0014
Xenobiotics	Benzoate Metabolism	hippurate	HMDB00714	0.4	0.1330	1.23	0.7491
Xenobiotics	Benzoate Metabolism	2-hydroxyhippurate (salicylurate)	HMDB00840	0.57	0.1420	1.23	0.6105
Xenobiotics	Benzoate Metabolism	3-hydroxyhippurate	HMDB06116	**0.48**	0.0940	1.25	0.6159
Xenobiotics	Benzoate Metabolism	4-hydroxyhippurate	HMDB13678	**0.35**	0.0570	1.36	0.5851
Xenobiotics	Benzoate Metabolism	4-hydroxymandelate	HMDB00822	0.61	0.2176	1.31	0.5274
Xenobiotics	Benzoate Metabolism	benzoate	HMDB01870	0.89	0.6220	0.96	0.8821
Xenobiotics	Benzoate Metabolism	4-hydroxybenzoate	HMDB00500	0.88	0.6260	1.8	0.0272
Xenobiotics	Benzoate Metabolism	3-hydroxybenzoate	HMDB02466	1.24	0.5385	1.08	0.8367
Xenobiotics	Benzoate Metabolism	2,4,6-trihydroxybenzoate	HMDB29649	0.92	0.8619	1.43	0.4928
Xenobiotics	Benzoate Metabolism	3,4-dihydroxybenzoate	HMDB01856	0.83	0.6844	1.96	0.1685
Xenobiotics	Benzoate Metabolism	catechol sulfate	HMDB59724	1.2	0.7046	1.64	0.3322
Xenobiotics	Benzoate Metabolism	4-methylcatechol sulfate		1.13	0.7115	1.3	0.4690
Xenobiotics	Benzoate Metabolism	p-hydroxybenzaldehyde	HMDB11718	0.79	0.6014	1.95	0.1528
Xenobiotics	Benzoate Metabolism	methyl-4-hydroxybenzoate	HMDB32572	0.57	0.0299	0.9	0.7061
Xenobiotics	Benzoate Metabolism	4-ethylphenylsulfate		1	1.0000	1.19	0.1087
Xenobiotics	Benzoate Metabolism	4-vinylphenol sulfate	HMDB04072	0.97	0.8993	1.11	0.6532
Xenobiotics	Benzoate Metabolism	3-methoxycatechol sulfate (1)		0.98	0.9277	1.34	0.2239
Xenobiotics	Benzoate Metabolism	methyl-4-hydroxybenzoate sulfate		0.92	0.7914	1.43	0.2899
Xenobiotics	Benzoate Metabolism	propyl 4-hydroxybenzoate	HMDB32574	0.95	0.1981	0.96	0.3164
Xenobiotics	Benzoate Metabolism	propyl 4-hydroxybenzoate sulfate		1.23	0.2524	1	0.9802
Xenobiotics	Benzoate Metabolism	p-cresol	HMDB01858	0.96	0.9482	1.36	0.6217
Xenobiotics	Benzoate Metabolism	p-cresol sulfate	HMDB11635	0.73	0.6344	0.67	0.5550
Xenobiotics	Benzoate Metabolism	o-cresol sulfate		1	0.9679	1.05	0.5902
Xenobiotics	Benzoate Metabolism	phenylpropionylglycine	HMDB00860	1.6	0.2274	1.09	0.8282
Xenobiotics	Benzoate Metabolism	2-methylbutyrylphenylalanine		0.72	0.4425	1.52	0.3361
Xenobiotics	Benzoate Metabolism	3-(3-hydroxyphenyl)propionate sulfate		0.9	0.8253	1.55	0.3685
Xenobiotics	Benzoate Metabolism	3-(3-hydroxyphenyl)propionate	HMDB00375	1.03	0.9652	1.28	0.7287
Xenobiotics	Benzoate Metabolism	3-(4-hydroxyphenyl)propionate	HMDB02199	1.08	0.8898	6.94	0.0007
Xenobiotics	Benzoate Metabolism	3-phenylpropionate (hydrocinnamate)	HMDB00764	2.01	0.2463	0.91	0.8777
Xenobiotics	Xanthine Metabolism	paraxanthine	HMDB01860	1.27	0.4562	1.17	0.6330
Xenobiotics	Xanthine Metabolism	theobromine	HMDB02825	1	0.9980	0.68	0.3008
Xenobiotics	Xanthine Metabolism	theophylline	HMDB01889	0.86	0.6266	0.44	0.0091

Xenobiotics	Xanthine Metabolism	1-methylurate	HMDB03099	1.06	0.8976	1.05	0.9228
Xenobiotics	Xanthine Metabolism	7-methylurate	HMDB11107	0.62	0.2774	0.92	0.8609
Xenobiotics	Xanthine Metabolism	1,3-dimethylurate	HMDB01857	0.74	0.4749	1.11	0.8098
Xenobiotics	Xanthine Metabolism	1,7-dimethylurate	HMDB11103	0.74	0.5596	1.47	0.4542
Xenobiotics	Xanthine Metabolism	3,7-dimethylurate	HMDB01982	1.04	0.9384	1.08	0.8762
Xenobiotics	Xanthine Metabolism	1,3,7-trimethylurate	HMDB02123	0.79	0.5763	1.06	0.8940
Xenobiotics	Xanthine Metabolism	1-methylxanthine	HMDB10738	0.98	0.9426	0.86	0.6562
Xenobiotics	Xanthine Metabolism	3-methylxanthine	HMDB01886	0.93	0.8408	**0.55**	0.0916
Xenobiotics	Xanthine Metabolism	5-acetylamino-6-amino-3-methyluracil	HMDB04400	0.79	0.6607	1.01	0.9838
Xenobiotics	Xanthine Metabolism	5-acetylamino-6-formylamino-3-methyluracil	HMDB11105	1.05	0.9049	1.04	0.9215
Xenobiotics	Xanthine Metabolism	caffeic acid sulfate	HMDB41708	0.91	0.8037	1.12	0.7600
Xenobiotics	Food Component/Plant	maltol	HMDB30776	0.9	0.5747	0.95	0.8153
Xenobiotics	Food Component/Plant	piperidine	HMDB34301	1.41	0.3320	0.86	0.6818
Xenobiotics	Food Component/Plant	2-piperidinone	HMDB11749	0.94	0.8768	0.96	0.9299
Xenobiotics	Food Component/Plant	kaempferol	HMDB05801	1.27	0.6236	1.52	0.4146
Xenobiotics	Food Component/Plant	kaempferol-3-rhamnoside		1		1	
Xenobiotics	Food Component/Plant	dihydrokaempferol	HMDB30847	0.92	0.8667	1.09	0.8751
Xenobiotics	Food Component/Plant	quercitrin	HMDB33751	1.09	0.8248	1.17	0.6879
Xenobiotics	Food Component/Plant	indoleacetylaspartate	HMDB38666	1.1	0.7543	**2.14**	0.0134
Xenobiotics	Food Component/Plant	erythrose	HMDB02649	0.55	0.2569	0.98	0.9725
Xenobiotics	Food Component/Plant	sucralose	HMDB31554	2.51	0.1693	0.79	0.7311
Xenobiotics	Food Component/Plant	genistein	HMDB03217	**0.34**	0.0769	0.81	0.7389
Xenobiotics	Food Component/Plant	3-dehydroshikimate		0.82	0.6965	0.89	0.8272
Xenobiotics	Food Component/Plant	catechin	HMDB02780	0.89	0.7170	0.96	0.9077
Xenobiotics	Food Component/Plant	epicatechin	HMDB01871	1.12	0.6533	0.89	0.6721
Xenobiotics	Food Component/Plant	1-methyl-beta-carboline-3-carboxylic acid		0.85	0.6275	1.06	0.8564
Xenobiotics	Food Component/Plant	diaminopimelate	HMDB01370	1.28	0.5148	1.24	0.5802
Xenobiotics	Food Component/Plant	1-kestose	HMDB11729	0.57	0.2077	1.43	0.4366
Xenobiotics	Food Component/Plant	apigenin	HMDB02124	0.7	0.4986	1.16	0.7830
Xenobiotics	Food Component/Plant	luteolin	HMDB05800	0.55	0.2694	1.07	0.9083
Xenobiotics	Food Component/Plant	levulinate (4-oxovalerate)	HMDB00720	1.14	0.6335	0.71	0.2171
Xenobiotics	Food Component/Plant	vanillate	HMDB00484	0.82	0.5775	**2.27**	0.0246
Xenobiotics	Food Component/Plant	2,3-dihydroxyisovalerate	HMDB12141	0.65	0.2899	0.7	0.3880
Xenobiotics	Food Component/Plant	2,8-quinolinediol		0.94	0.8968	1.17	0.7464

Xenobiotics	Food Component/Plant	2,8-quinolinediol sulfate		1.44	0.3805	**2.14**	0.0846
Xenobiotics	Food Component/Plant	20-hydroxyecdysone	HMDB30180	0.96	0.8670	0.86	0.5683
Xenobiotics	Food Component/Plant	2-isopropylmalate	HMDB00402	1.2	0.7211	0.88	0.8076
Xenobiotics	Food Component/Plant	2-oxindole-3-acetate	HMDB35514	1.25	0.6189	1.83	0.1879
Xenobiotics	Food Component/Plant	3,5-dihydroxybenzoic acid	HMDB13677	**1.5**	0.0546	1.06	0.7754
Xenobiotics	Food Component/Plant	4-hydroxybenzyl alcohol	HMDB11724	1.06	0.8650	1.68	0.1742
Xenobiotics	Food Component/Plant	gluconate	HMDB00625	1.16	0.8017	2.44	0.1445
Xenobiotics	Food Component/Plant	2-acetolactate	HMDB06833	1.01	0.9810	1.09	0.7308
Xenobiotics	Food Component/Plant	apiin		**0.8**	0.0667	0.88	0.3489
Xenobiotics	Food Component/Plant	beta-guanidinopropanoate	HMDB13222	0.73	0.3985	1.15	0.7159
Xenobiotics	Food Component/Plant	caffeate	HMDB01964	0.46	0.2835	2.64	0.1980
Xenobiotics	Food Component/Plant	chrysoeriol	HMDB30667	0.69	0.4838	1.54	0.4264
Xenobiotics	Food Component/Plant	coumaroylquinate (1)		0.89	0.7935	1.54	0.3380
Xenobiotics	Food Component/Plant	coumaroylquinate (2)		1.02	0.9191	0.97	0.8920
Xenobiotics	Food Component/Plant	coumaroylquinate (3)		1	0.9930	1.28	0.2698
Xenobiotics	Food Component/Plant	coumaroylquinate (4)		0.92	0.5933	1.01	0.9683
Xenobiotics	Food Component/Plant	coumaroylquinate (5)		1.06	0.7593	1	0.9976
Xenobiotics	Food Component/Plant	cryptochlorogenic acid		0.69	0.2018	0.72	0.2820
Xenobiotics	Food Component/Plant	daidzein	HMDB03312	0.41	0.1369	0.53	0.3065
Xenobiotics	Food Component/Plant	deoxymugineic acid		0.6	0.2733	**4.5**	0.0021
Xenobiotics	Food Component/Plant	digalacturonic acid	HMDB39721	1.34	0.5550	0.62	0.3537
Xenobiotics	Food Component/Plant	dihydroferulic acid		0.63	0.4522	**6.99**	0.0032
Xenobiotics	Food Component/Plant	dihydroquercetin		0.95	0.8679	0.85	0.6083
Xenobiotics	Food Component/Plant	enterolactone		1.91	0.1988	1.14	0.7973
Xenobiotics	Food Component/Plant	equol sulfate		0.82	0.3446	1.04	0.8524
Xenobiotics	Food Component/Plant	eriodictyol	HMDB05810	0.62	0.4002	1.31	0.6467
Xenobiotics	Food Component/Plant	erythritol	HMDB02994	0.49	0.1772	0.95	0.9294
Xenobiotics	Food Component/Plant	ferulate	HMDB00954	1.24	0.6403	**3.5**	0.0100
Xenobiotics	Food Component/Plant	ferulic acid 4-sulfate	HMDB29200	1.14	0.8494	**4.88**	0.0274
Xenobiotics	Food Component/Plant	ferulylglycine (1)		**0.4**	0.0256	**2.16**	0.0665
Xenobiotics	Food Component/Plant	fucitol		0.57	0.1015	1.32	0.4324
Xenobiotics	Food Component/Plant	glycitein	HMDB05781	0.54	0.1195	0.7	0.3797
Xenobiotics	Food Component/Plant	glycyrrhetinate	HMDB11628	1		1	
Xenobiotics	Food Component/Plant	hesperetin	HMDB05782	**0.28**	0.0647	0.65	0.5465

Xenobiotics	Food Component/Plant	indoleacrylate	HMDB00734	1.85	0.1317	0.78	0.5663
Xenobiotics	Food Component/Plant	indolin-2-one		1.17	0.7388	0.62	0.3251
Xenobiotics	Food Component/Plant	isovitexin		1.05	0.9119	1.27	0.5695
Xenobiotics	Food Component/Plant	methyl indole-3-acetate	HMDB29738	0.69	0.1778	1.26	0.4184
Xenobiotics	Food Component/Plant	N-(2-furoyl)glycine	HMDB00439	**0.62**	0.0932	1.01	0.9776
Xenobiotics	Food Component/Plant	naringenin	HMDB02670	0.64	0.4783	0.84	0.7920
Xenobiotics	Food Component/Plant	N-glycolylneuraminate	HMDB00833	0.77	0.2565	1.02	0.9366
Xenobiotics	Food Component/Plant	nicotianamine		0.37	0.1351	2.99	0.1092
Xenobiotics	Food Component/Plant	pheophorbide A		1.05	0.9323	0.46	0.2056
Xenobiotics	Food Component/Plant	phytanate	HMDB00801	1.01	0.9768	1.12	0.8120
Xenobiotics	Food Component/Plant	piperine	HMDB29377	1.11	0.7986	0.94	0.8930
Xenobiotics	Food Component/Plant	ponciretin		0.61	0.3313	1.88	0.2317
Xenobiotics	Food Component/Plant	quinate	HMDB03072	1.19	0.7376	1.12	0.8341
Xenobiotics	Food Component/Plant	rosmarinate	HMDB03572	**0.57**	0.0206	1.28	0.3008
Xenobiotics	Food Component/Plant	saccharin	HMDB29723	1.78	0.4042	1.16	0.8351
Xenobiotics	Food Component/Plant	acesulfame	HMDB33585	0.49	0.3540	1.97	0.3945
Xenobiotics	Food Component/Plant	secoisolariciresinol	HMDB13692	0.76	0.4516	1.51	0.2763
Xenobiotics	Food Component/Plant	sinapate	HMDB32616	0.91	0.8010	**1.9**	0.0882
Xenobiotics	Food Component/Plant	solanidine	HMDB03236	0.79	0.7392	0.89	0.8705
Xenobiotics	Food Component/Plant	soyasaponin I	HMDB34649	0.79	0.6918	2.18	0.2059
Xenobiotics	Food Component/Plant	soyasaponin II	HMDB34650	0.52	0.1238	0.65	0.3384
Xenobiotics	Food Component/Plant	soyasaponin III		0.87	0.7942	0.8	0.6928
Xenobiotics	Food Component/Plant	stachydrine	HMDB04827	0.65	0.3577	0.75	0.5442
Xenobiotics	Food Component/Plant	syringic acid	HMDB02085	0.64	0.3822	2.08	0.1708
Xenobiotics	Food Component/Plant	tartarate	HMDB00956	1.47	0.3922	0.95	0.9210
Xenobiotics	Food Component/Plant	tribuloside		1	1.0000	**1.13**	0.0616
Xenobiotics	Food Component/Plant	tyrosol	HMDB04284	1.01	0.9832	**1.98**	0.0412
Xenobiotics	Food Component/Plant	vitexin		0.48	0.1505	2.21	0.1271
Xenobiotics	Food Component/Plant	methyl glucopyranoside (alpha + beta)		0.73	0.3141	0.9	0.7565
Xenobiotics	Food Component/Plant	capsaicin	HMDB02227	0.98	0.9412	0.86	0.5188
Xenobiotics	Food Component/Plant	hesperidin	HMDB03265	0.65	0.4507	0.6	0.4026
Xenobiotics	Food Component/Plant	sinigrin	HMDB34070	1	0.9796	1	0.9744
Xenobiotics	Food Component/Plant	enterodiol		1.53	0.3754	1.1	0.8449
Xenobiotics	Food Component/Plant	limonin		0.69	0.1313	1.01	0.9653

Xenobiotics	Food Component/Plant	narirutin		0.98	0.9781	0.62	0.2312
Xenobiotics	Food Component/Plant	neoponcirin		0.99	0.9754	0.91	0.7390
Xenobiotics	Food Component/Plant	harmane	HMDB35196	1	1.0000	1.04	0.9076
Xenobiotics	Food Component/Plant	diosmetin	HMDB29676	0.29	0.0192	1.25	0.6872
Xenobiotics	Food Component/Plant	synephrine	HMDB04826	0.75	0.1673	1.01	0.9795
Xenobiotics	Food Component/Plant	nomilin		0.83	0.5771	0.9	0.7641
Xenobiotics	Food Component/Plant	pyrraline	HMDB33143	0.56	0.1115	1.56	0.2327
Xenobiotics	Food Component/Plant	umbelliferone sulfate		1.06	0.8178	1.27	0.3639
Xenobiotics	Food Component/Plant	daidzein sulfate (2)		0.39	0.0123	1.38	0.4129
Xenobiotics	Food Component/Plant	N-acetylpyrraline		0.93	0.7928	1.05	0.8678
Xenobiotics	Food Component/Plant	daidzein sulfate (1)		0.35	0.0023	1.04	0.9083
Xenobiotics	Food Component/Plant	2-keto-3-deoxy-gluconate	HMDB01353	0.76	0.5048	1	0.9966
Xenobiotics	Food Component/Plant	3-methylsulfinylpropyl-glucosinolate	HMDB38406	1		1	
Xenobiotics	Food Component/Plant	kaempferol 3-O-glucoside/galactoside		1		1	
Xenobiotics	Food Component/Plant	1,2-dilinolenoyl-digalactosylglycerol (18:3/18:3)		0.97	0.8932	0.64	0.1021
Xenobiotics	Food Component/Plant	1-palmitoyl-2-linolenoyl-digalactosylglycerol (16:0/18:3)		0.9	0.7860	0.67	0.3250
Xenobiotics	Food Component/Plant	dihydrocaffeate	HMDB00423	2.17	0.2086	0.97	0.9646
Xenobiotics	Food Component/Plant	4-hydroxycinnamate	HMDB02035	1.67	0.2786	2.01	0.1566
Xenobiotics	Food Component/Plant	DIMBOA		1.01	0.9574	1.13	0.5366
Xenobiotics	Food Component/Plant	Urolithin A	HMDB13695	1.33	0.2855	1.18	0.5365
Xenobiotics	Food Component/Plant	luteolin-8-C-glucoside		0.87	0.7626	2.04	0.1241
Xenobiotics	Food Component/Plant	malonylgenistin		0.51	0.0023	0.99	0.9670
Xenobiotics	Bacterial/Fungal	2-acetamidobutanoate		0.95	0.9003	0.92	0.8331
Xenobiotics	Bacterial/Fungal	glutamyl-meso-diaminopimelate		1.31	0.5884	0.45	0.1286
Xenobiotics	Bacterial/Fungal	N-acetylmuramyl-alanyl-isoglutamine		0.64	0.2656	1.77	0.1734
Xenobiotics	Bacterial/Fungal	N-methylpipecolate		0.66	0.1234	0.96	0.8696
Xenobiotics	Bacterial/Fungal	Urolithin B	HMDB13696	0.96	0.8379	0.97	0.9008
Xenobiotics	Drug - Analgesics, Anesthetics	4-acetamidophenol	HMDB01859	0.61	0.4231	0.6	0.4287
Xenobiotics	Drug - Analgesics, Anesthetics	3-(N-acetyl-L-cystein-S-yl) acetaminophen		0.68	0.5408	0.83	0.7780
Xenobiotics	Drug - Analgesics, Anesthetics	4-acetaminophen sulfate	HMDB59911	0.87	0.8338	0.57	0.4359
Xenobiotics	Drug - Analgesics, Anesthetics	4-acetamidophenylglucuronide	HMDB10316	0.99	0.0484	1	1.0000
Xenobiotics	Drug - Analgesics, Anesthetics	2-hydroxyacetaminophen sulfate*		0.84	0.6788	1.01	0.9829
Xenobiotics	Drug - Analgesics, Anesthetics	2-methoxyacetaminophen sulfate*		0.75	0.5363	0.85	0.7320

Xenobiotics	Drug - Analgesics, Anesthetics	2-methoxyacetaminophen glucuronide*		**0.77**	0.0049	1	1.0000
Xenobiotics	Drug - Analgesics, Anesthetics	3-(cystein-S-yl)acetaminophen*		0.91	0.7472	0.96	0.8792
Xenobiotics	Drug - Analgesics, Anesthetics	2-acetamidophenol sulfate		0.93	0.7815	1.25	0.4052
Xenobiotics	Drug - Analgesics, Anesthetics	4-aminophenol sulfate (2)		1.14	0.3558	0.95	0.7482
Xenobiotics	Drug - Analgesics, Anesthetics	carboxyibuprofen	HMDB60564	1	1.0000	0.97	0.1620
Xenobiotics	Drug - Analgesics, Anesthetics	diclofenac	HMDB14724	1		1	
Xenobiotics	Drug - Antibiotic	azithromycin	HMDB14352	0.55	0.1555	1	1.0000
Xenobiotics	Drug - Antibiotic	amoxicillin	HMDB15193	0.8	0.2421	0.96	0.8232
Xenobiotics	Drug - Antibiotic	sulfamethoxazole	HMDB15150	**0.99**	0.0803	0.99	0.3564
Xenobiotics	Drug - Antibiotic	metronidazole	HMDB15052	1	0.9518	1	0.8696
Xenobiotics	Drug - Antibiotic	clotrimazole	HMDB01922	0.88	0.6602	0.94	0.8433
Xenobiotics	Drug - Antibiotic	fluconazole	HMDB14342	1		1	
Xenobiotics	Drug - Antibiotic	3-hydroxyquinine	HMDB01091	1	0.9788	1	0.9186
Xenobiotics	Drug - Antibiotic	quinine		1	1.0000	0.99	0.8757
Xenobiotics	Drug - Gastrointestinal	promethazine	HMDB15202	1	1.0000	0.84	0.2494
Xenobiotics	Drug - Gastrointestinal	ranitidine	HMDB01930	1		1	
Xenobiotics	Drug - Respiratory	diphenhydramine	HMDB01927	1	0.9313	1	1.0000
Xenobiotics	Drug - Respiratory	loratadine	HMDB05000	1.05	0.5450	1	1.0000
Xenobiotics	Drug - Topical Agents	salicylate	HMDB01895	**1.78**	0.0970	**4.67**	0.0000
Xenobiotics	Drug - Topical Agents	hydroquinone sulfate	HMDB02434	1.03	0.9321	**1.94**	0.0898
Xenobiotics	Drug - Other	S-carboxymethyl-L-cysteine	HMDB29415	**0.46**	0.0857	0.98	0.9662
Xenobiotics	Chemical	1,3-propanediol		1.09	0.8425	1.77	0.2279
Xenobiotics	Chemical	3-hydroxypyridine		1.54	0.1970	1.05	0.8788
Xenobiotics	Chemical	sulfate*	HMDB01448	0.89	0.6110	0.89	0.6485
Xenobiotics	Chemical	O-sulfo-L-tyrosine		0.43	0.1445	1.15	0.8168
Xenobiotics	Chemical	2-aminophenol sulfate	HMDB61116	0.87	0.6264	1	1.0000
Xenobiotics	Chemical	1,4-butanediol		1	1.0000	1.08	0.7333
Xenobiotics	Chemical	S-(3-hydroxypropyl)mercapturic acid (HPMA)		0.87	0.7359	1.28	0.5669
Xenobiotics	Chemical	5-aminoimidazole-4-carboxamide	HMDB03192	0.52	0.1253	0.91	0.8232
Xenobiotics	Chemical	dexpanthenol	HMDB04231	0.9	0.5031	1	1.0000
Xenobiotics	Chemical	ectoine		0.9	0.7470	0.85	0.6109
Xenobiotics	Chemical	HEPES		1	0.9419	1	0.9732
Xenobiotics	Chemical	N-propionylmethionine		1.09	0.8631	**3.6**	0.0111

Xenobiotics	Chemical	azeloylcarnitine (C9-DC)		0.84	0.4583	0.97	0.8935
Xenobiotics	Chemical	succinimide		0.84	0.3722	0.84	0.3862
Xenobiotics	Chemical	triethanolamine	HMDB32538	0.7	0.2556	0.95	0.8765
Xenobiotics	Chemical	trizma acetate		1		1	
Xenobiotics	Chemical	2,4-dihydroxyhydrocinnamate		0.96	0.8770	1	0.9923
Xenobiotics	Chemical	4-hydroxychlorothalonil		1.35	0.2771	1	0.9876
Xenobiotics	Chemical	4-thiouracil		0.92	0.7698	1.21	0.4962
Xenobiotics	Chemical	1,2,3-benzenetriol sulfate (2)		0.73	0.4073	**2.01**	0.0820
Xenobiotics	Chemical	1,2,3-benzenetriol sulfate (1)		0.91	0.6512	1.21	0.3785
Xenobiotics	Chemical	4-acetamidobenzoate		0.97	0.9339	1.21	0.5847
Xenobiotics	Chemical	thioproline		0.59	0.2682	1.53	0.3952
N/A	N/A	amodiaquine	HMDB14751	1	0.9262	1	0.9533
N/A	N/A	X - 02249 - retired for 3-Cmpfp**		0.68	0.2019	1.63	0.1223
N/A	N/A	X - 09789		0.85	0.6316	**2.63**	0.0056
N/A	N/A	X - 10457		0.83	0.4117	1.17	0.4845
N/A	N/A	X - 10458		1	0.9591	0.97	0.5751
N/A	N/A	X - 11261		0.88	0.6734	1.15	0.6702
N/A	N/A	X - 11308		0.66	0.2841	0.95	0.8969
N/A	N/A	X - 11357		0.75	0.5199	1.04	0.9281
N/A	N/A	X - 11407		0.8	0.2742	**2.03**	0.0016
N/A	N/A	X - 11429 - retired for 5,6-dihydrouridine		0.59	0.2394	1.81	0.2054
N/A	N/A	X - 11440		0.91	0.8549	0.93	0.8832
N/A	N/A	X - 11441		0.89	0.7432	0.75	0.4288
N/A	N/A	X - 11442		1	0.9931	0.74	0.4539
N/A	N/A	X - 11444		**0.61**	0.0260	1.16	0.5270
N/A	N/A	X - 11452 - retired for sulfate of piperine metabolite C16H19NO3 (2)*		0.54	0.1618	1.25	0.6269
N/A	N/A	X - 11470		**0.65**	0.0426	1.11	0.6496
N/A	N/A	X - 11491		**0.39**	0.0859	1.02	0.9670
N/A	N/A	X - 11522		0.82	0.5580	0.83	0.6015
N/A	N/A	X - 11530		1.04	0.9272	0.78	0.6179
N/A	N/A	X - 11538 - retired for octadecenedioate (C18:1-DC)		0.99	0.9659	1.66	0.1209
N/A	N/A	X - 11540 - retired for 5-dodecenoylcarnitine		0.89	0.8305	2.16	0.1906
N/A	N/A	X - 11564		0.72	0.3368	0.84	0.6176

N/A	N/A	X - 11612		0.77	0.5860	1	1.0000
N/A	N/A	X - 11632		0.95	0.8590	1.11	0.6956
N/A	N/A	X - 11640		1.65	0.1854	1.57	0.2476
N/A	N/A	X - 11648		0.94	0.9022	0.75	0.5854
N/A	N/A	X - 11787		0.86	0.5765	1.3	0.3348
N/A	N/A	X - 11795		0.95	0.9117	0.9	0.8360
N/A	N/A	X - 11838		1.94	0.1077	0.78	0.5692
N/A	N/A	X - 11905 - retired for hexadecenedioate (C16:1-DC)*		1.31	0.1936	1.19	0.4101
N/A	N/A	X - 11979		0.78	0.1856	1	1.0000
N/A	N/A	X - 12007		0.76	0.5094	1.22	0.6536
N/A	N/A	X - 12013		1.01	0.9834	1.49	0.4063
N/A	N/A	X - 12015		1.42	0.4815	1.3	0.6089
N/A	N/A	X - 12026		0.8	0.4982	1.4	0.3335
N/A	N/A	X - 12027		1.09	0.7638	**1.93**	0.0341
N/A	N/A	X - 12093		0.85	0.6892	0.93	0.8525
N/A	N/A	X - 12096		0.77	0.4924	1.28	0.5190
N/A	N/A	X - 12100		**0.66**	0.0729	1.07	0.7722
N/A	N/A	X - 12101		0.73	0.4638	0.76	0.5319
N/A	N/A	X - 12117		**0.57**	0.0542	0.91	0.7511
N/A	N/A	X - 12125		1.34	0.3882	0.77	0.4716
N/A	N/A	X - 12127		0.54	0.2367	1.21	0.7226
N/A	N/A	X - 12199		**1.66**	0.0373	0.69	0.1538
N/A	N/A	X - 12206		0.51	0.1658	1.14	0.7910
N/A	N/A	X - 12214		1.22	0.6166	**2.29**	0.0452
N/A	N/A	X - 12216		0.69	0.5277	2.02	0.2538
N/A	N/A	X - 12230		0.94	0.4542	1.1	0.2951
N/A	N/A	X - 12231 - retired for sulfate of piperine metabolite C16H19NO3 (3)*		0.74	0.2179	0.95	0.8475
N/A	N/A	X - 12261		0.8	0.4064	**1.76**	0.0406
N/A	N/A	X - 12263		0.56	0.1169	**2.24**	0.0406
N/A	N/A	X - 12398		0.69	0.5023	1.26	0.6829
N/A	N/A	X - 12410		0.74	0.6510	1.95	0.3403
N/A	N/A	X - 12411		1.05	0.9014	1.24	0.6289
N/A	N/A	X - 12442		0.75	0.2680	0.94	0.8170

N/A	N/A	X - 12456		1.05	0.8815	0.66	0.1976
N/A	N/A	X - 12462		1.05	0.8960	0.55	0.1346
N/A	N/A	X - 12472		0.66	0.4420	0.86	0.7882
N/A	N/A	X - 12511 - retired for N-acetyl-2-aminoctanoate		1.04	0.8763	0.85	0.5667
N/A	N/A	X - 12565		2.28	0.1893	1.12	0.8641
N/A	N/A	X - 12680		**0.46**	0.0305	1.01	0.9751
N/A	N/A	X - 12688 - retired for N-acetyl-isoputrenine*		0.73	0.2086	0.84	0.5009
N/A	N/A	X - 12695 - retired for alpha-keto-glutaramate**		0.61	0.1805	1.09	0.8250
N/A	N/A	X - 12721		0.98	0.9082	1.12	0.5003
N/A	N/A	X - 12733		0.68	0.2983	1.38	0.4012
N/A	N/A	X - 12739		0.6	0.1513	0.69	0.3132
N/A	N/A	X - 12740		1.34	0.5365	0.71	0.4818
N/A	N/A	X - 12753		**0.69**	0.0596	**0.64**	0.0360
N/A	N/A	X - 12813		1.39	0.5568	0.98	0.9774
N/A	N/A	X - 12814 - retired for glucuronide of C12H22O4 (1)		0.99	0.9392	1.22	0.3282
N/A	N/A	X - 12815		1.06	0.7569	1.06	0.7581
N/A	N/A	X - 12818		0.79	0.5143	0.76	0.4510
N/A	N/A	X - 12821		0.88	0.8380	1.61	0.4461
N/A	N/A	X - 12822		0.64	0.4222	1.17	0.7952
N/A	N/A	X - 12828		1.37	0.3924	**2.43**	0.0227
N/A	N/A	X - 12831 - retired for glucuronide of C14H26O4 (1)*		0.93	0.5892	0.98	0.8804
N/A	N/A	X - 12839		0.89	0.6468	0.82	0.4835
N/A	N/A	X - 12844		**0.62**	0.0171	0.98	0.9299
N/A	N/A	X - 12849		0.79	0.3067	1.15	0.5433
N/A	N/A	X - 12879		0.97	0.9579	1.66	0.4320
N/A	N/A	X - 12906		1.42	0.4200	1.59	0.3118
N/A	N/A	X - 13007		1.23	0.4304	**1.62**	0.0789
N/A	N/A	X - 13431		1.06	0.9019	**2.66**	0.0615
N/A	N/A	X - 13507		0.63	0.1269	1.36	0.3219
N/A	N/A	X - 13553		0.94	0.8884	2.04	0.1538
N/A	N/A	X - 13723		0.76	0.3371	**2.13**	0.0140
N/A	N/A	X - 13729		0.79	0.5451	0.79	0.5597
N/A	N/A	X - 13737		0.78	0.2476	1	0.9889

N/A	N/A	X - 13834 - retired for nonenedioate (C9:1-DC)*		1.03	0.8903	**0.5**	0.0009
N/A	N/A	X - 13835		1.04	0.9119	1.42	0.3639
N/A	N/A	X - 13844		**0.51**	0.0781	1.53	0.2791
N/A	N/A	X - 14056		0.51	0.1318	1.91	0.1594
N/A	N/A	X - 14095 - retired for pyr-ala*		0.8	0.5220	0.89	0.7585
N/A	N/A	X - 14096 - retired for pyr-thr*		0.56	0.1274	1.07	0.8567
N/A	N/A	X - 14099 - retired for pyr-his*		0.88	0.7561	1.04	0.9278
N/A	N/A	X - 14113 - retired for pyr-arg*		0.93	0.8453	1.63	0.2256
N/A	N/A	X - 14141 - retired for alpha-glu-leu*		1.53	0.2085	0.72	0.3617
N/A	N/A	X - 14196 - retired for pyr-tyr*		0.78	0.4523	1.09	0.7947
N/A	N/A	X - 14224		0.9	0.4552	1.17	0.2807
N/A	N/A	X - 14254		0.94	0.9141	1.08	0.8985
N/A	N/A	X - 14263		0.85	0.6264	0.87	0.6919
N/A	N/A	X - 14264		1.14	0.8075	0.86	0.7911
N/A	N/A	X - 14302 - retired for pyr-ile*		0.77	0.4565	1.14	0.7166
N/A	N/A	X - 14314 - retired for pyr-leu*		0.76	0.2651	1.17	0.5500
N/A	N/A	X - 14320		1.13	0.6199	0.84	0.5055
N/A	N/A	X - 14337		0.85	0.7686	1.41	0.5522
N/A	N/A	X - 14364 - retired for pyr-phe*		0.63	0.2294	0.53	0.1220
N/A	N/A	X - 14383		1.54	0.3751	1.2	0.7190
N/A	N/A	X - 14392		1.29	0.5764	1.67	0.2864
N/A	N/A	X - 14416		1.08	0.8799	1.39	0.5283
N/A	N/A	X - 14454		1.95	0.2201	1.02	0.9691
N/A	N/A	X - 14473		1.01	0.9798	0.72	0.1918
N/A	N/A	X - 14502		0.73	0.6550	2.2	0.2797
N/A	N/A	X - 14538		0.83	0.6578	0.95	0.9031
N/A	N/A	X - 14658		**0.29**	0.0300	**0.36**	0.0810
N/A	N/A	X - 14662		**0.61**	0.0087	0.93	0.7175
N/A	N/A	X - 14697 - retired for alpha-glu-ile*		1.09	0.8011	0.81	0.5619
N/A	N/A	X - 14838		0.67	0.3861	1.26	0.6277
N/A	N/A	X - 14900		1.94	0.1609	0.85	0.7327
N/A	N/A	X - 14904		1.33	0.2947	1.55	0.1208
N/A	N/A	X - 15136		0.79	0.4155	0.92	0.7730
N/A	N/A	X - 15150		1.35	0.3021	**0.6**	0.0921

N/A	N/A	X - 15245		0.77	0.5350	1.61	0.2739
N/A	N/A	X - 15461		1.04	0.9165	0.75	0.4493
N/A	N/A	X - 15486		0.69	0.3735	0.9	0.8057
N/A	N/A	X - 15492		0.66	0.0250	0.95	0.8006
N/A	N/A	X - 15497		0.4	0.1049	1.24	0.7098
N/A	N/A	X - 15608		1.21	0.5474	1	0.9994
N/A	N/A	X - 15646 - retired for gamma-CEHC sulfate*		0.97	0.9594	1.69	0.3422
N/A	N/A	X - 15666		0.85	0.4616	0.84	0.4686
N/A	N/A	X - 15843		0.83	0.5957	0.82	0.5761
N/A	N/A	X - 15853		0.93	0.8746	1.14	0.7829
N/A	N/A	X - 15854		1.29	0.6308	1.29	0.6463
N/A	N/A	X - 16060		0.95	0.8672	1.13	0.7222
N/A	N/A	X - 16071		0.56	0.1128	0.96	0.9101
N/A	N/A	X - 16343		0.96	0.9075	0.77	0.5163
N/A	N/A	X - 16391		1.09	0.8337	1.35	0.4679
N/A	N/A	X - 16580		0.72	0.3445	0.57	0.1210
N/A	N/A	X - 16654		0.39	0.1331	0.61	0.4489
N/A	N/A	X - 16946		0.87	0.6199	0.81	0.4640
N/A	N/A	X - 16947 - retired for glucuronide of C10H18O2 (7)*		0.96	0.6733	0.98	0.8544
N/A	N/A	X - 17009		1.13	0.7736	1.14	0.7739
N/A	N/A	X - 17010		1.19	0.4967	1.17	0.5454
N/A	N/A	X - 17162		0.94	0.8931	0.72	0.5169
N/A	N/A	X - 17299		0.56	0.4040	0.95	0.9387
N/A	N/A	X - 17325		0.79	0.5045	1.09	0.8125
N/A	N/A	X - 17327		0.83	0.6099	0.96	0.9108
N/A	N/A	X - 17335		0.75	0.2720	0.62	0.0866
N/A	N/A	X - 17348		2.34	0.0483	1.49	0.3815
N/A	N/A	X - 17349		0.63	0.4161	0.9	0.8596
N/A	N/A	X - 17353		1.05	0.8643	1.09	0.7536
N/A	N/A	X - 17359		0.65	0.0004	0.91	0.4531
N/A	N/A	X - 17438		0.9	0.7595	2.21	0.0213
N/A	N/A	X - 17469		1.12	0.7864	1.24	0.6099
N/A	N/A	X - 17653		0.85	0.6541	0.94	0.8739

N/A	N/A	X - 17655		1	1.0000	**0.63**	0.0477
N/A	N/A	X - 17674		0.84	0.7002	1.16	0.7455
N/A	N/A	X - 17686		0.81	0.4324	1.27	0.3840
N/A	N/A	X - 17705 - retired for N-acetylglucosamine conjugate of C24H40O4 bile acid**		0.63	0.2211	0.99	0.9691
N/A	N/A	X - 17709		0.61	0.1914	1.88	0.1061
N/A	N/A	X - 17749		0.8	0.5283	1.17	0.6660
N/A	N/A	X - 17808		0.74	0.4741	**2.11**	0.0983
N/A	N/A	X - 17838		2.02	0.1577	1.56	0.3925
N/A	N/A	X - 17842		0.9	0.7750	1.33	0.4726
N/A	N/A	X - 17852		0.89	0.8244	1.99	0.1987
N/A	N/A	X - 17869		0.76	0.4842	1.09	0.8428
N/A	N/A	X - 17876		1.07	0.8663	1.58	0.2598
N/A	N/A	X - 17877		0.95	0.9067	1.68	0.2915
N/A	N/A	X - 17895		0.77	0.4596	1.74	0.1307
N/A	N/A	X - 17910		0.96	0.9150	1.21	0.6617
N/A	N/A	X - 17919		0.86	0.7440	1.27	0.6135
N/A	N/A	X - 17960		1.53	0.3704	1.93	0.1861
N/A	N/A	X - 17969		1.13	0.8004	1.67	0.3037
N/A	N/A	X - 17978		1.21	0.7195	1.08	0.8840
N/A	N/A	X - 17983		1.18	0.7308	1.03	0.9557
N/A	N/A	X - 17984		1.23	0.5369	1.06	0.8651
N/A	N/A	X - 18059		0.95	0.7891	1.22	0.3287
N/A	N/A	X - 18162		1.2	0.6325	0.85	0.6896
N/A	N/A	X - 18165		0.88	0.7590	0.81	0.6285
N/A	N/A	X - 18167		0.81	0.7113	1.16	0.8005
N/A	N/A	X - 18278		1.72	0.3327	1.38	0.5807
N/A	N/A	X - 18345		0.95	0.9119	1.26	0.6217
N/A	N/A	X - 18349		0.92	0.7736	0.82	0.5144
N/A	N/A	X - 18410		0.5	0.1896	0.83	0.7307
N/A	N/A	X - 18888		0.73	0.4269	0.69	0.3823
N/A	N/A	X - 18889		0.44	0.1349	1.22	0.7322
N/A	N/A	X - 18935		0.9	0.7769	1.22	0.6089
N/A	N/A	X - 18938		1.04	0.8696	1.44	0.1177

N/A	N/A	X - 19220		0.55	0.1264	0.95	0.8989
N/A	N/A	X - 19232		0.83	0.4885	0.98	0.9295
N/A	N/A	X - 19299		0.85	0.6587	0.81	0.5687
N/A	N/A	X - 19369		0.97	0.9377	1.64	0.1614
N/A	N/A	X - 19434		**0.43**	0.0438	0.94	0.8916
N/A	N/A	X - 19452		**0.41**	0.0140	0.7	0.3587
N/A	N/A	X - 19561		0.58	0.2255	1.86	0.1872
N/A	N/A	X - 19746		0.56	0.2275	1.32	0.5849
N/A	N/A	X - 19751		0.58	0.2310	1.16	0.7536
N/A	N/A	X - 19763		0.83	0.6450	1.31	0.5251
N/A	N/A	X - 19914		1	1.0000	1.04	0.8921
N/A	N/A	X - 19917		1		1	
N/A	N/A	X - 19920		1.28	0.1022	1.11	0.5154
N/A	N/A	X - 19921		0.64	0.1987	1.49	0.2649
N/A	N/A	X - 19923		0.85	0.3865	1.02	0.9148
N/A	N/A	X - 19924		1.18	0.5333	1.09	0.7540
N/A	N/A	X - 19928		0.69	0.4377	1.43	0.4801
N/A	N/A	X - 19929		0.71	0.2551	1.29	0.4235
N/A	N/A	X - 19931		0.79	0.5843	1.22	0.6710
N/A	N/A	X - 19932 - retired for pyr-trp*		0.71	0.3745	0.97	0.9347
N/A	N/A	X - 19934		1.06	0.7580	1.07	0.7279
N/A	N/A	X - 19940		0.71	0.3453	1.06	0.8768
N/A	N/A	X - 20100		1.23	0.6478	1.44	0.4345
N/A	N/A	X - 20172		1.28	0.6485	1.23	0.7079
N/A	N/A	X - 20185		1.11	0.8128	1.29	0.5680
N/A	N/A	X - 20197		1.33	0.5683	1.4	0.5146
N/A	N/A	X - 20624		0.93	0.8756	1.29	0.5964
N/A	N/A	X - 20747		1.62	0.3410	1.02	0.9655
N/A	N/A	X - 20756		0.56	0.1261	1.26	0.5559
N/A	N/A	X - 20768		0.71	0.2243	1.05	0.8619
N/A	N/A	X - 21283		0.95	0.8882	1.29	0.5421
N/A	N/A	X - 21285		**0.55**	0.0115	0.69	0.1221
N/A	N/A	X - 21295		0.52	0.2689	**3.02**	0.0760
N/A	N/A	X - 21319		1.36	0.3090	1.36	0.3409

N/A	N/A	X - 21327		1.02	0.9554	**5.52**	0.0000
N/A	N/A	X - 21343 - retired for dodecadienoate (12:2)*		1.09	0.8419	0.8	0.6187
N/A	N/A	X - 21353		0.77	0.2892	0.98	0.9277
N/A	N/A	X - 21410		0.92	0.8287	0.69	0.3318
N/A	N/A	X - 21441		0.81	0.1065	0.99	0.9542
N/A	N/A	X - 21448		0.85	0.5731	0.89	0.6964
N/A	N/A	X - 21467		0.63	0.3074	1.2	0.7062
N/A	N/A	X - 21470		0.85	0.6916	**0.49**	0.0990
N/A	N/A	X - 21471		0.83	0.6066	1.01	0.9858
N/A	N/A	X - 21668 - retired for glyco-beta-muricholate**		**0.54**	0.0692	1.03	0.9344
N/A	N/A	X - 21729		1.1	0.8513	1.61	0.3661
N/A	N/A	X - 21785		0.68	0.5688	1.03	0.9621
N/A	N/A	X - 21787		1.15	0.6711	1.44	0.2678
N/A	N/A	X - 21788		0.91	0.8724	0.82	0.7383
N/A	N/A	X - 21796		0.97	0.8393	0.89	0.5157
N/A	N/A	X - 21821		0.75	0.4065	1.04	0.9166
N/A	N/A	X - 21826 - retired for glutamine conjugate of C8H12O2 (2)*		0.69	0.3117	0.82	0.6094
N/A	N/A	X - 21851		0.82	0.1452	0.93	0.6358
N/A	N/A	X - 21959		1.18	0.5593	1.62	0.1005
N/A	N/A	X - 21963		1.03	0.8922	1	0.9925
N/A	N/A	X - 22030		1.75	0.1365	1.67	0.1944
N/A	N/A	X - 22035 - retired for pyr-pro*		**0.4**	0.0221	1.01	0.9736
N/A	N/A	X - 22062		1.27	0.4404	1.21	0.5516
N/A	N/A	X - 22085		0.65	0.4472	0.92	0.8946
N/A	N/A	X - 22099		**0.63**	0.0942	0.89	0.6743
N/A	N/A	X - 22102		0.46	0.1974	0.38	0.1366
N/A	N/A	X - 22142		0.51	0.1690	0.78	0.6254
N/A	N/A	X - 22162		0.7	0.3323	**0.42**	0.0261
N/A	N/A	X - 22520		1.05	0.8670	0.9	0.7258
N/A	N/A	X - 22800		0.94	0.8700	1.21	0.6232
N/A	N/A	X - 22810		0.91	0.7234	0.85	0.5296
N/A	N/A	X - 23159		0.67	0.1431	0.89	0.6844
N/A	N/A	X - 23160		0.86	0.6686	0.97	0.9292

N/A	N/A	X - 23163 - retired for gamma-CEHC taurine*		0.65	0.1645	1.22	0.5343
N/A	N/A	X - 23164 - retired for alpha-CEHC taurine*		0.68	0.1656	0.99	0.9670
N/A	N/A	X - 23173		0.83	0.6689	1.23	0.6607
N/A	N/A	X - 23188		1.38	0.2805	1.58	0.1403
N/A	N/A	X - 23191		0.89	0.7751	3.99	0.0012
N/A	N/A	X - 23193		0.81	0.1833	1.39	0.0413
N/A	N/A	X - 23196		1.23	0.4223	2.07	0.0087
N/A	N/A	X - 23201		0.96	0.9190	0.78	0.5465
N/A	N/A	X - 23223		1.02	0.9727	1.84	0.2594
N/A	N/A	X - 23228		0.93	0.7286	1.03	0.8801
N/A	N/A	X - 23236		0.91	0.7356	1.5	0.1373
N/A	N/A	X - 23238		0.68	0.1609	0.77	0.3572
N/A	N/A	X - 23259		1	1.0000	1.49	0.0180
N/A	N/A	X - 23267		0.72	0.4557	4.42	0.0015
N/A	N/A	X - 23277		0.9	0.6752	2.15	0.0035
N/A	N/A	X - 23287		0.92	0.8007	1.11	0.7364
N/A	N/A	X - 23314		1.03	0.9384	0.64	0.2075
N/A	N/A	X - 23323		1.14	0.7629	2.22	0.0762
N/A	N/A	X - 23324		1.17	0.6762	1.59	0.2340
N/A	N/A	X - 23360		0.99	0.9587	1.28	0.0719
N/A	N/A	X - 23369		0.56	0.1425	0.8	0.5913
N/A	N/A	X - 23423		0.91	0.7456	0.93	0.8110
N/A	N/A	X - 23424		0.87	0.5441	0.88	0.5820
N/A	N/A	X - 23429		0.93	0.8538	1.06	0.8865
N/A	N/A	X - 23432		2.74	0.0475	0.89	0.8275
N/A	N/A	X - 23438		0.83	0.3770	0.97	0.8813
N/A	N/A	X - 23451		0.49	0.2067	2.67	0.0905
N/A	N/A	X - 23453		3.25	0.0096	2.02	0.1326
N/A	N/A	X - 23456		1.08	0.8781	1.38	0.5567
N/A	N/A	X - 23469		1.05	0.9137	1.58	0.3414
N/A	N/A	X - 23470		1.2	0.6473	2.34	0.0379
N/A	N/A	X - 23481		0.88	0.4814	1.08	0.6610
N/A	N/A	X - 23482		0.86	0.6274	1.83	0.0648
N/A	N/A	X - 23507		0.93	0.7490	1.18	0.4914

N/A	N/A	X - 23557		0.8	0.6430	1.32	0.5861
N/A	N/A	X - 23581		0.55	0.2937	2.19	0.1860
N/A	N/A	X - 23585		1.03	0.9517	0.9	0.8414
N/A	N/A	X - 23587		0.79	0.6581	0.9	0.8470
N/A	N/A	X - 23600		1.15	0.7536	1	0.9935
N/A	N/A	X - 23610		0.91	0.7263	0.84	0.5424
N/A	N/A	X - 23617		0.68	0.1647	1.24	0.4538
N/A	N/A	X - 23623		0.82	0.3763	1.14	0.5769
N/A	N/A	X - 23626		**0.57**	0.0924	1.18	0.6375
N/A	N/A	X - 23637		0.84	0.5972	1.25	0.5066
N/A	N/A	X - 23639		1.13	0.7483	1.24	0.5983
N/A	N/A	X - 23644		0.5	0.1664	0.97	0.9559
N/A	N/A	X - 23652		0.89	0.6565	1.18	0.5473
N/A	N/A	X - 23655		1.31	0.3958	1.05	0.8894
N/A	N/A	X - 23662		0.74	0.5629	0.76	0.6038
N/A	N/A	X - 23665		0.98	0.9606	1.05	0.8940
N/A	N/A	X - 23680		1.13	0.7230	0.86	0.6786
N/A	N/A	X - 23710		0.91	0.8216	0.93	0.8550
N/A	N/A	X - 23728		1	0.9993	**2.68**	0.0266
N/A	N/A	X - 23729		0.87	0.6462	1.06	0.8647
N/A	N/A	X - 23732		1.25	0.6047	**2.25**	0.0763
N/A	N/A	X - 23734		0.89	0.7210	1	0.9882
N/A	N/A	X - 23736		0.75	0.3788	0.93	0.8400
N/A	N/A	X - 23737		0.9	0.7393	1.46	0.2490
N/A	N/A	X - 23739		0.72	0.5322	1.7	0.3283
N/A	N/A	X - 23747 - retired for diacetylspermidine*		0.89	0.6873	0.97	0.9237
N/A	N/A	X - 23753		1.05	0.9008	**2.64**	0.0179
N/A	N/A	X - 23756 - retired for octadecenedioylcarnitne (C18:1-DC)*		1.62	0.2369	1.21	0.6408
N/A	N/A	X - 23764		1.21	0.5788	1.32	0.4344
N/A	N/A	X - 23767		0.6	0.3481	0.87	0.8039
N/A	N/A	X - 23776		0.57	0.3648	0.93	0.9054
N/A	N/A	X - 23782		0.72	0.3158	1.03	0.9251
N/A	N/A	X - 23908		0.97	0.9522	**0.3**	0.0154

N/A	N/A	X - 23919		1.06	0.8081	1.07	0.8022
N/A	N/A	X - 23925		1.54	0.2258	0.82	0.5811
N/A	N/A	X - 23939		0.94	0.1063	1	1.0000
N/A	N/A	X - 23942		0.73	0.2677	1.03	0.9154
N/A	N/A	X - 23974		0.94	0.8026	0.85	0.5430
N/A	N/A	X - 24027		0.88	0.3017	1.04	0.7242
N/A	N/A	X - 24077		0.46	0.1560	**3.62**	0.0238
N/A	N/A	X - 24084		0.91	0.8209	0.95	0.9042
N/A	N/A	X - 24089		**0.61**	0.0605	1.1	0.7371
N/A	N/A	X - 24137		1.05	0.8681	1.06	0.8404
N/A	N/A	X - 24143		1.06	0.8488	1.01	0.9828
N/A	N/A	X - 24177		0.75	0.6176	1.28	0.6854
N/A	N/A	X - 24207 - retired for pheophytin A		0.97	0.9534	1.41	0.5815
N/A	N/A	X - 24208		**0.51**	0.0733	1.32	0.4702
N/A	N/A	X - 24216		**0.37**	0.0284	**0.39**	0.0451
N/A	N/A	X - 24220		0.73	0.1973	0.9	0.6887
N/A	N/A	X - 24231		0.95	0.9085	1.57	0.3712
N/A	N/A	X - 24246		0.61	0.3182	0.7	0.4912
N/A	N/A	X - 24272		0.99	0.9228	1.22	0.1038
N/A	N/A	X - 24329		0.66	0.1723	1.09	0.7859
N/A	N/A	X - 24348		**0.34**	0.0108	1.38	0.4518
N/A	N/A	X - 24359		0.73	0.6364	1.12	0.8657
N/A	N/A	X - 24400		0.8	0.5223	0.96	0.9100
N/A	N/A	X - 24404 - retired for umbelliferone		0.66	0.2520	0.92	0.8226
N/A	N/A	X - 24408		0.81	0.5387	1.3	0.4659
N/A	N/A	X - 24410		1.32	0.5951	1.19	0.7505
N/A	N/A	X - 24412		0.89	0.6197	0.71	0.1571
N/A	N/A	X - 24413		0.74	0.6373	0.91	0.8949
N/A	N/A	X - 24425		0.88	0.7417	1.73	0.1717
N/A	N/A	X - 24431		0.95	0.9132	1.25	0.6272
N/A	N/A	X - 24452		**0.41**	0.0100	1.1	0.7874
N/A	N/A	X - 24455		0.85	0.3791	1.16	0.4595
N/A	N/A	X - 24456		0.86	0.4861	1.16	0.5218
N/A	N/A	X - 24465		**0.55**	0.0577	0.92	0.7922

N/A	N/A	X - 24474		1.03	0.9118	1.29	0.3961
N/A	N/A	X - 24542		**0.23**	0.0048	1.89	0.2344
N/A	N/A	X - 24544		0.82	0.6920	0.89	0.8216
N/A	N/A	X - 24545		0.57	0.1219	0.56	0.1159
N/A	N/A	X - 24546		0.9	0.7600	**0.54**	0.0872
N/A	N/A	X - 24550 - retired for PEG-glycoside, fragment 07*		0.81	0.4078	1.01	0.9625
N/A	N/A	X - 24551 - retired for PEG-glucuronide, fragment 04*		0.92	0.7088	0.99	0.9737
N/A	N/A	X - 24552 - retired for PEG-glycoside, fragment 05*		0.68	0.1605	1.08	0.7962
N/A	N/A	X - 24555 - retired for PEG-glycoside, fragment 04*		0.83	0.4712	1.07	0.8145
N/A	N/A	X - 24556		0.83	0.6155	1.79	0.1161
N/A	N/A	X - 24557 - retired for PEG-glucuronide, fragment 06*		0.99	0.9527	0.97	0.8730
N/A	N/A	X - 24558 - retired for PEG-glycoside, fragment 06*		0.76	0.3202	0.97	0.9065
N/A	N/A	X - 24559 - retired for PEG-glycoside, fragment 03*		0.82	0.3917	1.06	0.8167
N/A	N/A	X - 24560 - retired for PEG-glycoside, fragment 08*		1.02	0.9186	1	0.9953
N/A	N/A	X - 24568 - retired for N4-acetylsulfamethoxazole*		0.77	0.4473	**0.29**	0.0008
N/A	N/A	X - 24569 - retired for N4-acetyl-5-hydroxysulfamethoxazole*		0.84	0.2967	**0.64**	0.0164
N/A	N/A	X - 24574		0.72	0.4498	0.54	0.1728
N/A	N/A	X - 24586		**0.43**	0.0083	0.83	0.5857
N/A	N/A	X - 24588		**0.32**	0.0039	0.94	0.8926
N/A	N/A	X - 24590		0.78	0.3164	1.19	0.4895
N/A	N/A	X - 24608		0.77	0.4059	0.6	0.1143
N/A	N/A	X - 24609		**0.56**	0.0221	0.85	0.5530
N/A	N/A	X - 24626		0.75	0.2529	0.89	0.6641
N/A	N/A	X - 24630		0.94	0.4853	1	1.0000
N/A	N/A	X - 24640		1.28	0.5952	**2.88**	0.0281
N/A	N/A	X - 24641		0.72	0.5406	2.23	0.1447
N/A	N/A	X - 24643		1	1.0000	1	1.0000
N/A	N/A	X - 24655 - retired for genistein sulfate		**0.33**	0.0145	1.62	0.3039
N/A	N/A	X - 24658		0.88	0.7169	1.08	0.8304
N/A	N/A	X - 24659		**0.39**	0.0086	0.75	0.4493
N/A	N/A	X - 24660		0.46	0.1139	1.36	0.5446
N/A	N/A	X - 24669		0.45	0.1267	1.53	0.4300
N/A	N/A	X - 24670		**0.41**	0.0135	1.06	0.8713

N/A	N/A	X - 24682 - retired for pyr-pro*		**0.27**	0.0123	0.79	0.6686
N/A	N/A	X - 24683		0.64	0.2615	1.13	0.7672
N/A	N/A	X - 24686		0.56	0.1136	0.96	0.9067
N/A	N/A	X - 24693 - retired for histidine betaine (hercynine)*		1.05	0.9245	1.45	0.4650
N/A	N/A	X - 24697		1.05	0.8975	1.24	0.5681
N/A	N/A	X - 24704		**0.45**	0.0178	0.81	0.5540
N/A	N/A	X - 24711		**0.37**	0.0028	1.2	0.5789
N/A	N/A	X - 24712		**0.42**	0.0029	0.8	0.4736
N/A	N/A	X - 24715		0.77	0.3621	1.03	0.9279
N/A	N/A	X - 24723		1	0.9809	1	1.0000
N/A	N/A	X - 24728		0.92	0.8668	1	0.9968
N/A	N/A	X - 24729		1.46	0.1115	1.17	0.5269
N/A	N/A	X - 24738 - retired for 3-hydroxystachydrine*		0.54	0.2658	0.94	0.9085
N/A	N/A	X - 24753		0.79	0.5365	**2.07**	0.0617
N/A	N/A	X - 24762		0.47	0.1858	0.86	0.8069
N/A	N/A	X - 24766		**0.62**	0.0784	1.07	0.8034
N/A	N/A	X - 24767		1.13	0.7502	**1.95**	0.0918
N/A	N/A	X - 24809		0.94	0.8767	0.65	0.3038
N/A	N/A	X - 24813		1.16	0.5039	1.35	0.1990
N/A	N/A	X - 24829		0.85	0.6443	1.1	0.7957
N/A	N/A	X - 24831		**0.6**	0.0220	**1.47**	0.0963
N/A	N/A	X - 24832		0.68	0.1318	1.49	0.1256
N/A	N/A	X - 24840		0.65	0.2312	1.3	0.4772
N/A	N/A	X - 24853		1.13	0.6746	0.78	0.4212
N/A	N/A	X - 24854		**0.46**	0.0482	0.82	0.6188
N/A	N/A	X - 24855		0.98	0.9476	0.74	0.4258
N/A	N/A	X - 24931		0.79	0.4395	0.79	0.4669
N/A	N/A	X - 24932		0.48	0.2154	1.43	0.5654
N/A	N/A	X - 24947		**0.54**	0.0522	**0.57**	0.0785
N/A	N/A	X - 24948		1.02	0.9685	0.93	0.8765
N/A	N/A	X - 24974		0.64	0.1729	**0.48**	0.0308
N/A	N/A	X - 24977		0.33	0.1115	**0.3**	0.0975
N/A	N/A	X - 24978		1.13	0.7157	0.95	0.8925
N/A	N/A	X - 24983		0.74	0.2085	0.86	0.5350

N/A	N/A	X - 24989		1.1	0.7792	1.29	0.4745
N/A	N/A	X - 24991		1.17	0.7046	1.12	0.7854
N/A	N/A	X - 24996		**0.46**	0.0057	1.1	0.7491
N/A	N/A	X - 25005		0.76	0.3725	1.22	0.5271
N/A	N/A	X - 25007		1.13	0.7052	1.51	0.2355
N/A	N/A	X - 25009		0.61	0.1128	1.32	0.3946
N/A	N/A	X - 25010		0.74	0.2707	0.92	0.7594
N/A	N/A	X - 25035		**0.4**	0.0896	**3.71**	0.0221
N/A	N/A	X - 25053		**0.39**	0.0609	1.48	0.4605
N/A	N/A	X - 25076		1.33	0.5756	1.72	0.3106

91
